# Next‐Generation Sequencing vs. Clinical‐Pathological Assessment in Diagnosis of Multiple Lung Cancers: A Systematic Review and Meta‐Analysis

**DOI:** 10.1111/1759-7714.70039

**Published:** 2025-03-21

**Authors:** Ziyang Wang, Xiaoqiu Yuan, Yuntao Nie, Jun Wang, Guanchao Jiang, Kezhong Chen

**Affiliations:** ^1^ Department of Thoracic Surgery Peking University People's Hospital Beijing China; ^2^ Thoracic Oncology Institute Peking University People's Hospital Beijing China; ^3^ Research Unit of Intelligence Diagnosis and Treatment in Early Non‐small Cell, Lung Cancer Chinese Academy of Medical Sciences, Peking University, People's Hospital Beijing China; ^4^ China‐Japan Friendship Hospital Beijing China

**Keywords:** clinical‐pathological evaluation, meta‐analysis, multiple lung cancers, next‐generation sequencing, systematic review

## Abstract

Accurately distinguishing between multiple primary lung cancers (MPLC) and intrapulmonary metastasis (IPM) is crucial for tailoring treatment strategies and improving patient outcomes. While molecular methods offer significant advantages over traditional clinical‐pathological evaluations, they lack standardized diagnostic protocols and validated prognostic value. This study systematically compared the diagnostic and prognostic performance of molecular methods versus clinical‐pathological evaluations in diagnosing multiple lung cancers (MLCs), specifically focusing on the impact of next‐generation sequencing (NGS) parameters on diagnostic accuracy. A review of 41 studies encompassing 1266 patients revealed that two molecular methods, Mole1 (manually counting shared mutations) and Mole2 (bioinformatics‐assisted clonal probability calculation), both demonstrated superior diagnostic accuracy and prognostic discrimination capabilities. Molecular assessment, particularly Mole1, effectively stratified prognosis for MPLC and IPM, leading to significantly improved disease‐free survival (DFS: HR = 0.24, 95% CI: 0.15–0.39) and overall survival (OS: HR = 0.33, 95% CI: 0.18–0.58). Further analysis suggests that a minimal panel of 30–50 genes may be sufficient to effectively differentiate prognoses. Compared to Mole1, Mole2 demonstrated greater specificity and stability across various panels, achieving AUC values from 0.962 to 0.979. Clinical‐pathological evaluations proved unreliable, not only failing to distinguish prognosis effectively but also exhibiting a potential misdiagnosis rate of 35.5% and 33.6% compared to the reference diagnosis. To improve both cost‐effectiveness and diagnostic accuracy, bioinformatics‐assisted molecular diagnostics should be integrated into multidisciplinary assessments, especially for high‐risk cases where diagnostic errors are common.

## Graphical abstract

This study compares next‐generation sequencing (NGS) with clinical‐pathological methods for diagnosing multiple lung cancers (MLCs). Leveraging Bayesian latent class models (LCM) and prognostic stratification, bioinformatics‐assisted molecular approaches demonstrated superior specificity and stability, reducing misclassification versus traditional assessments. Integrated workflows combining histopathologic triage and targeted molecular analysis enhanced diagnostic precision in differentiating MLCs, guiding tailored therapeutic strategies while optimizing cost‐effectiveness

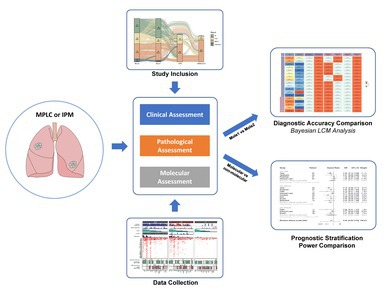

## 1 Introduction

Currently, lung cancer ranks as the most common malignancy and remains the leading cause of cancer‐related mortality worldwide [1]. With the widespread use of thin‐slice chest computed tomography (CT), multiple lung cancers (MLCs) have become a common clinical challenge [2, 3]. MLCs comprise multiple primary lung cancers (MPLC) and intrapulmonary metastasis (IPM). MPLC generally has a better prognosis, often treated with curative resection, whereas IPM typically requires additional therapies with poorer outcomes [4]. Misclassification of MLC subtypes can result in suboptimal treatment strategies and potentially severe therapeutic errors, underscoring the critical importance of accurate diagnosis.

The diagnosis of MLCs traditionally relies on clinical and pathological information, such as the Martini‐Melamed criteria [5], the American College of Chest Physicians (ACCP) guideline [6], and the International Association for the Study of Lung Cancer (IASLC) proposal [7], along with histopathological evaluations (HPE) like comprehensive histology assessment (CHA) [8]. However, the advent of next‐generation sequencing (NGS) has revolutionized this, offering a potentially superior approach to the differential diagnosis of MLCs [9]. Despite this, a consensus on the optimal method or established protocol for assessing clonal relationships in MLCs remains elusive. Currently, most studies rely on manual interpretation of NGS results (Mole1), often employing subjective evaluation frameworks and manual mutation counting. The studies on this approach lack efficiency and standardization. While some studies have explored bioinformatics tools for clonal probability analysis (Mole2), these are not yet widely adopted [9]. Beyond the need for comparison between two representative molecular assessment methods, two more key challenges remain to be addressed: optimizing sequencing parameters, particularly determining the ideal sequencing coverage, and developing a cost‐effective diagnostic that integrates molecular and clinical data. This study aims to address these challenges through a systematic and comprehensive evaluation of both molecular and clinical‐pathological diagnostic approaches for MLCs, with the goal of paving the way for a more accurate, efficient, and standardized diagnostic process.

## 2 Methods

Our systematic review followed a preregistered protocol on PROSPERO (CRD42024612366) and adhered to the Preferred Reporting Items for Systematic Reviews and Meta‐Analyzes (PRISMA) guidelines to comprehensively evaluate the diagnostic and prognostic performance of molecular and clinical‐pathological methods for MLCs.

### 2.1 Search Strategy and Eligibility Criteria

The final search was conducted on March 1, 2024. A systematic search of PubMed, Web of Science, Embase, and Scopus was conducted, covering literature from database inception to March 2024. Search terms combined keywords such as “diagnosis” “lung neoplasms” and “high‐throughput sequencing” Additional manual screening of reference lists supplemented the search. We included studies that assessed the diagnostic and prognostic performance of molecular and clinical‐pathological methods for MLCs, while simultaneously providing extractable genomic profiling data. Studies with fewer than 8 patients, review articles, and case reports were excluded. Full search strategies and eligibility criteria are provided in the supplementary file.

### 2.2 Data Extraction and Quality Assessment

**FIGURE 1:**
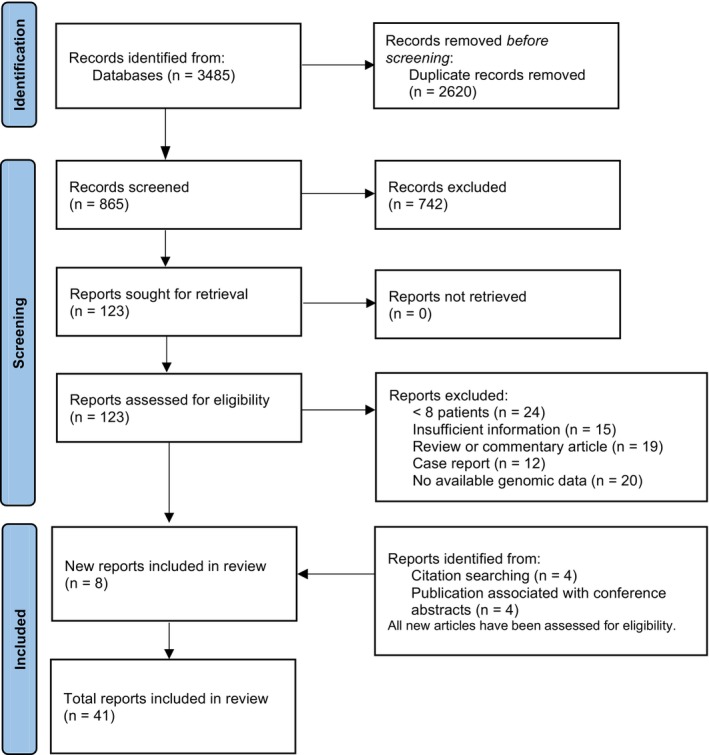
Flow diagram for study selection.

Two reviewers (Z.Y.W and X.Q.Y.) independently screened titles and abstracts, with eligible citations undergoing full‐text review. Data extraction included study design, sample size, patient demographics, diagnostic methods (e.g., panel size and criteria), and primary outcomes. Discrepancies were resolved through discussion with a third reviewer (Y.T.N). The selection process was summarized in a PRISMA flow diagram (Figure 1). Risk of bias was assessed independently by two authors (Z.Y.W and X.Q.Y.) using the QUADAS‐2 tool. Studies with high risk were included, with their limitations acknowledged.

### 2.3 Data Analysis

Diagnostic accuracy, assessed using sensitivity, specificity, integrated discrimination improvement (IDI), and area under the receiver operating characteristic curve (AUC), was determined via a Bayesian latent class model (LCM) (Tables S1 and S2). Prognostic stratification analysis employed hazard ratios (HRs) and 95% confidence intervals (CI) pooled using a random‐effects model. All statistical analyzes were conducted in R (version 4.3.1). Detailed descriptions of statistical modeling and mutation subsampling procedures are provided in the supplementary file.

## 3 Result

### 3.1 Study Selection and Quality Assessment

A comprehensive search across four major medical literature databases (PubMed, Web of Science, Embase, and Scopus) yielded 3485 potentially relevant studies (1618, 1210, 433, and 224, respectively). Following deduplication and careful screening, 41 articles met the inclusion criteria and were included in the quantitative analysis, encompassing 1266 patients (Figure 1) [10, 11, 12, 13, 14, 15, 16, 17, 18, 19, 20, 21, 22, 23, 24, 25, 26, 27, 28, 29, 30, 31, 32, 33, 34, 35, 36, 37, 38, 39, 40, 41, 42, 43, 44, 45, 46, 47, 48, 49, 50]. Literature quality was assessed using the QUADAS‐2 tool (Figure S1), revealing a high risk of bias in 22 of the 41 included articles. Our analysis identified three primary sources of this bias. Firstly, 54.5% (12/22) of high‐bias studies relied on non‐randomized case selection, including only MPLC cases confirmed through clinical‐pathological assessments, potentially introducing selection bias. Secondly, 45.5% (10/22) of high‐bias studies prioritized genomic characteristic analysis over evaluating the diagnostic performance of different molecular assessments. Lastly, 36.4% (8/22) of the studies exhibited verification bias due to outdated diagnostic reference standards. To mitigate the impact of high‐risk literature, we adopted a selective data extraction strategy, focusing on genomic data from these studies for subsequent molecular assessment and comparison of different molecular evaluation methods.

### 3.2 Characteristics of Included Studies

The characteristics of the patients and included studies are shown in Tables 1 and S3. Analysis of the extracted data revealed that 82.3% (1042/1266) were cases with double pulmonary nodules, and 36.8% (194/526) had a history of smoking. While multifocal ground‐glass opacities (GGO), often indicative of indolent MPLC, are not the primary focus of the included studies, only 86 cases were documented. The majority of cases were clinically and pathologically assessed as “early‐stage” nodules with no lymph node metastasis and a diameter of no more than 3 cm (staged as T1N0) if diagnosed as MPLC (Table 1). A total of 5182 tumor‐associated single nucleotide variants (SNVs) of tumor mutations were extracted and successfully underwent molecular assessment.

**TABLE 1:** Characteristics of included studies.

Author^a^	Study period	*N* (patients/tumors)	Synchronous (%)	Reference standard	No. (%) inconclusive cases	Diagnostic molecular methods^c^	No. (%) inconclusive Cases	Suvival analysis	Panel
Arai	1970–2010	12/24	100	HPE	0 (0)	Mole1	8 (67)	NA	1
Asmar	2005–2014	69/154	75.36	HPE	0 (0)	Mole1	29 (42)	NA	4
Belardinilli	2015–2019	10/24	60	CHA + IHC	0 (0)	Mole1	1 (10)	NA	22
Bruehl	2008–2019	32/64	100	HPE	0 (0)	Mole1	8 (25)	Yes	35 (17limited)
Chang	NA	60/128	75	CHA	23 (30)	Mole2	0 (0)	Yes	341–468
Chen	2017–2018	17/35	100	HPE	0 (0)	Mole1	1 (6)	Yes	168
Donfrancesco	2010–2018	24/50	100	CHA + IHC	0 (0)	Mole1	3 (13)	Yes	22
Duan	2016–2018	16/36	100	HPE	0 (0)	Mole1	0 (0)	NA	520
Ezer	2016–2020	61/131	NA	CHA	8 (13)	Mole1	8 (13)	Yes	15/52
Girard	2003–2008	7/14	42.86	MM/ACCP	0 (0)	Mole1	0 (0)	NA	2
Goodwin	2000–2019	40/80	52.5	MM/HPE	0 (0)	Mole1	0 (0)	Yes	50
Goto	2014–2016	24/48	100	Mole1	0 (0)	MoleX	0 (0)	NA	53
Higuchi	2015–2019	37/76	70.27	HPE	0 (0)	Mole1	NA	NA	53
Hu	2018–2020	112/255	92.9	MM	0 (0)	NA	NA	NA	1021
Izumi	2007–2019	17/38	NA	MM	0 (0)	NA	NA	NA	409
Lee	2017–2020	101/208	91.1	ACCP	0 (0)	Mole1	54 (54)	Yes	1
Li	2019–2021	41/94	100	MM	0 (0)	Mole1	5 (12)	NA	500
Liu	2013–2014	38/76	100	HPE	0 (0)	Mole1	4 (11)	NA	1
Liu (A)	NA	6/15	100	MM	0 (0)	Mole1	0 (0)	NA	WES
Liu (B)	NA	15/35	NA	HPE	1 (7)	Mole1	0 (0)	NA	464
Mansuet‐Lupo	2010–2012	120/240 (Included 30/60)	72	HPE	0 (0)	Mole1	7 (23)	Yes	22
Pagan	NA	47/108	100	ACCP	0 (0)	Mole1	12 (26)	NA	47
Patel	2014–2015	11/31	45.45	CHA	3 (27)	Mole1	0 (0)	Yes	50
Pei	2017–2019	30/67	83.3	MM	0 (0)	Mole1	0 (0)	NA	808
Qiu (A)	2019–2019	30/66	100	HPE	0 (0)	Mole1	1 (3)	NA	568
Qiu (B)	2008–2016	44/88	100	HPE	0 (0)	Mole1	26 (59)	NA	22
Qu	2016–2019	8/17	100	MM	0 (0)	Mole1	0 (0)	NA	1
Rodriguez	NA	30/60	50	Mole1	10 (33)	NA	NA	NA	8
Roepman	2003–2014	50/111	72	CHA + IHC	0 (0)	Mole1	14 (24)	Yes	50
Saab	2015–2015	18/52	33.33	HPE	6 (35)	CHA + NGS	1 (8)/5 (28)	NA	50
Takahashi	2002–2013	37/82	48.65	MM/HPE	MM:0 (0)	MoleX	0 (0)	Yes	20
					HPE:17 (46)				
Takamochi	1996–2008	30/68	83.33	MM	0 (0)	Mole1	5 (17)	Yes	2
Vincenten^b^	2007‐2015	6/13	83.3	ACCP	0 (0)	Mole1	3 (50)	NA	2
Wang	NA	Training cohort:35/80	100	HPE	0 (0)	Mole1	0 (0)	NA	605
		Validation cohort:16/36							
Xiao	2004–2015	6/14	100	Mole1	0 (0)	MoleX	NA	NA	50
Xu	2014–2017	50/101	100	ACCP	0 (0)	Mole1	7 (14)	Yes	10
Yang	2007–2020	24/48	45.83	Mole1	10 (42)	MoleX	0 (0)	NA	410–468
Zhang	2018–2021	42/93	100	ACCP	0 (0)	Mole1	3 (7)	NA	10
Zhang (B)	2018–2019	45/101	100	CHA	0 (0)	MoleX	0 (0)	Yes	425
Zheng	2013–2018	18/41	100	HPE	1 (5)	Mole1	3 (17)	NA	4/48
Zhou	2019–2019	19/60	100	MM	0 (0)	Mole1	0 (0)	NA	WES

Abbreviations: ACCP, American College of Chest Physicians; CHA, comprehensive histologic assessment; HPE, histopathological evaluation excluding CHA; HC, Immunohistochemistry; MM, criteria proposed by Martini and Melamed; WES, whole exome sequencing; NA, no report.

^a^ Author: Some studies include authors with the same surname, differentiated by suffixes.

^b^ Limited cases have extractable mutation data, but the full cohort is sufficiently large for comparison. Only cases with mutation data are shown.

^c^ MoleX refers to other rare molecular methods that employ bioinformatics techniques.

### 3.3 Prognostic Stratification Analysis

**FIGURE 2:**
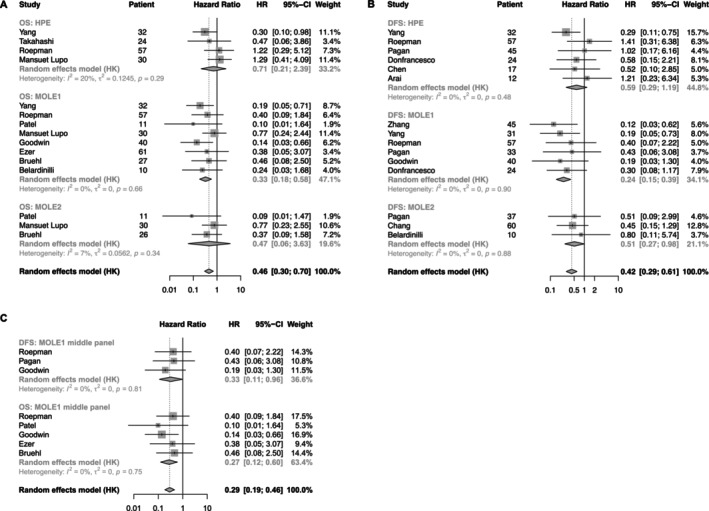
Prognostic analysis of different methods for distinguishing MLCs. (A) Prognostic analysis of OS comparing HPE, Mole1, and Mole2 methods. (B) Prognostic analysis of DFS comparing HPE, Mole1, and Mole2 methods. (C) *OS* and DFS analyzes using Mole1 with middle‐size sequencing panels (30–50 genes). OS, Overall survival; DFS, Disease‐free survival; HPE, histopathology evaluation.

Although definitive reference diagnoses are unavailable for the included cases, the ability to differentiate prognoses between MPLC and IPM has been proposed as a crucial benchmark for evaluating the efficacy of diagnostic methods in identifying clonal relationships [10, 12, 13, 14, 15, 16, 18, 20, 30, 31, 32, 38, 40, 46, 48]. As shown in Figure 2, clinical assessment and HPE showed limited effectiveness in distinguishing the prognosis of different MLCs subtypes, irrespective of whether overall survival (OS) or disease‐free survival (DFS) was considered. In contrast, molecular assessments, with the exception of Mole2's suboptimal performance in stratifying OS, exhibited superior performance to clinical‐histopathological assessments. Notably, Mole1 identified MPLC with a significantly lower risk of disease progression, yielding a HR of 0.24 (95% CI, 0.15–0.39), which was more favorable compared to the HR for OS, at 0.33 (95% CI, 0.18–0.58). Mole2 was slightly inferior to Mole1, with an HR for distinguishing DFS at 0.51 (95% CI, 0.27–0.98). No significant differences were observed between the results of different studies.

Further analysis revealed that Mole1 exhibited sufficient discriminatory power in studies employing sequencing panels comprising more than 50 genes, as evidenced by its ability to distinguish both DFS and OS (Figure S2). Even when gene panels were limited to 30–50 genes, Mole1 consistently demonstrated a significant capacity to differentiate MLC subtypes, yielding hazard ratios of 0.27 (95% CI, 0.12–0.60) for OS and 0.33 (95% CI, 0.11–0.96) for DFS (Figure 2C). However, this discriminatory power diminished with smaller panels employed.

### 3.4 Analysis of Sensitivity and Specificity in Molecular Assessments

By statistically analyzing the diagnostic performance of two molecular methods in simulated MLCs, we determined the necessary prior information. Based on this, we successfully employed a Bayesian latent class model for a comprehensive evaluation of two representative molecular assessments in included cases. We systematically compared the diagnostic performance of the two methods using several metrics, including sensitivity, specificity, IDI, AUC, and the difference in AUC (AUC.diff), based on predicted diagnoses generated by the LCM model.

**FIGURE 3:**
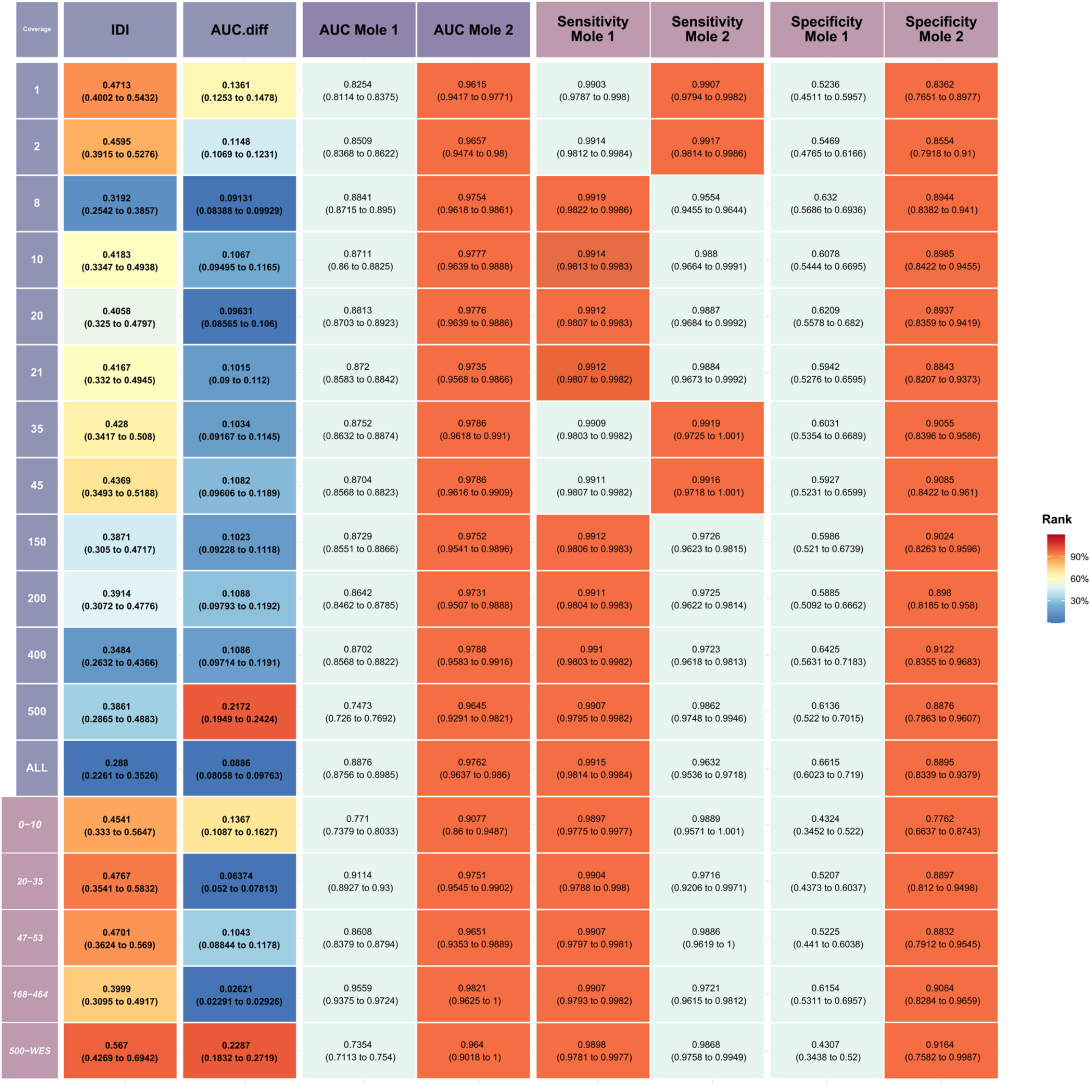
Comparative analysis of diagnostic performance metrics between Mole1 and Mole2. The figure illustrates the performance of Mole1 and Mole2 across various sequencing panels, displaying the IDI, AUC values, AUC.diff, sensitivity, and specificity. Color coding indicates the rank of diagnostic performance for each metric. Specifically, AUC, sensitivity, and specificity are compared for equivalent sequencing panels (within rows), with superior performance highlighted in orange‐red. IDI and AUC.diff, conversely, rank performance across different panels (within columns). IDI, Integrated Discrimination Improvement; AUC, Area Under the Curve.

IDI quantifies the difference in predicted probabilities between the two groups, while AUC.diff assesses the overall discriminatory ability of Mole2 versus Mole1 and evaluates the clinical risk of misdiagnosis by comparing the AUC of the two methods. Both molecular assessment methods demonstrated high accuracy in definitively diagnosed cases. Mole1 achieved an AUC range of 0.747 to 0.888, while Mole2 ranged from 0.962 to 0.979 (Figure 3). Analyzes of IDI and AUC.diff indicate that Mole2 outperformed Mole1 across various sequencing coverages. Notably, the performance gap widened as panel size decreased, a trend particularly evident in the panel grouping analysis. This trend was less pronounced in the subsampling strategy analysis. Sensitivity and specificity for both methods exhibited an increasing trend with expanding panel size. While both Mole1 and Mole2 demonstrated high and remarkably similar sensitivity values, Mole2 consistently outperformed Mole1 in specificity (Mole1: 0.524–0.662 vs. Mole2: 0.836–0.912), particularly with smaller sequencing panels.

Further analysis of indeterminate (IN) rates reveals the instability of Mole1's performance compared to Mole2. Mole1 produced a significantly higher proportion of IN results (Mole1 43.0% vs. Mole2 32.2%), particularly with panels containing fewer than 50 genes (Figure S3). To achieve comparable diagnostic accuracy to Mole2, Mole1 requires integration with a larger sequencing panel (greater than 100 genes). This finding is further supported by the concordance analysis between the two methods. Even after excluding panels with fewer than 10 genes, agreement remained relatively low across most panel sizes, ranging from 76.6% to 80.6% (Figure S4). Notably, as the sequencing panel expanded, Mole2 demonstrated improved accuracy in identifying inconclusive cases initially diagnosed as such by Mole1 (11.1%–16.9%), while the proportion of Mole1 diagnoses being modified to inconclusive by Mole2 remained below 1.7%. In panel‐grouped analysis, the improvement rate reached 7.6%–19.7%, while conversions remained under 3.4%.

These findings suggest that subjective judgment methods like Mole1 may compromise the effectiveness of molecular assessments compared to more objective methods like Mole2, which exhibit greater stability and reliability. Subgroup analyzes further support these findings, despite minor differences (Figure 3).

### 3.5 Threshold Effect of Gene Panel Size on Molecular Diagnostic Accuracy

**FIGURE 4:**
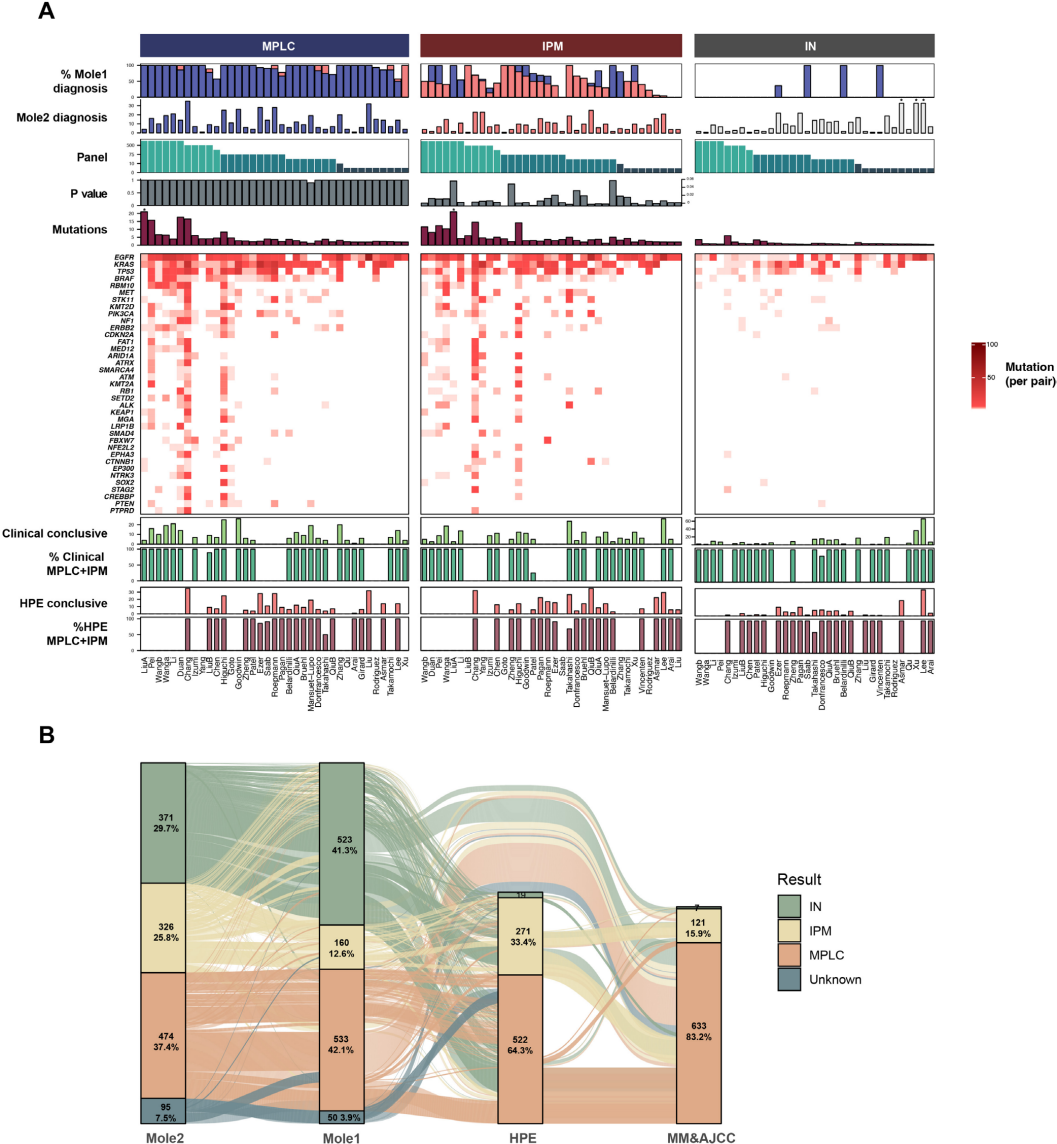
Overview of included studies and diagnostic tracking for included cases. (A) Multidimensional diagnostic landscape was stratified by Mole2 reference classifications, encompassing corresponding Mole1 molecular classification results, tumor mutation heatmaps, gene panel sizes, and diagnostic accuracy rates for clinical or histopathological evaluations. (B) The Sankey diagram visualizes longitudinal shifts in case classifications across clinical, pathological, and molecular evaluations within a cohort of 1266 cases, highlighting diagnostic reclassifications across different approaches.

Analysis of molecular assessment results across different panel sizes in all cases revealed a threshold effect on both tumor mutation detection rates and definitive diagnosis rates: beyond a certain sequencing range, the benefits of further panel expansion diminished significantly (Table S4). For example, studies utilizing panels exceeding 100 genes consistently achieved definitive diagnoses in 85%–95% of included cases, with minimal difference between studies. Moreover, both the tumor mutation detection rate and improvement rates exhibited rapid initial growth before plateauing (Figures 4A and S3). These findings suggest that in real‐world applications of NGS‐based molecular assessment, there is no need to indiscriminately expand molecular testing. Utilizing a panel of an appropriate size is sufficient to meet diagnostic requirements without placing an unnecessary burden on patients.

### 3.6 Discrepancies in Diagnostic Accuracies Between Different Diagnostic Assessments

A significant discrepancy exists between clinical‐pathological evaluations and molecular assessments. Using Mole2 diagnoses as the reference standard, we found that clinical assessments, including the Martini‐Melamed criteria and the non‐molecular parts of ACCP criteria and IASLC proposal [10, 13, 15, 16, 17, 20, 22, 23, 24, 25, 26, 28, 29, 30, 32, 33, 34, 36, 40, 41, 43, 45, 48, 49, 50], demonstrate limited diagnostic accuracy, with potential misjudgments observed in at least one‐third of all cases. Subsampling analyzes revealed that the proportion of cases with diagnoses concordant with Mole2 ranged from 16.4% (1 gene) to 66.0% (over 500 genes). Panel‐grouped analyzes yielded concordance rates between 16.4%–47.6% (fewer than 10 genes) and a maximum of 61.8% (more than 100 genes). The concordance rate between pathological and molecular assessments is even lower, with over 40% of cases showing diagnostic changes in both subsampling and panel‐grouped analyzes (Figure 4B).

The aforementioned findings suggest that rigidly adhering to traditional clinicopathological criteria may be counterproductive in differentiating MLCs. To avoid inconsistencies between diagnostic standards and circular diagnostic processes, we propose a tiered evaluation framework that integrates clinical‐pathological assessment criteria with enhanced specificity or sensitivity alongside molecular evaluation, as supported by previous studies and consensus guidelines [14, 30, 51]. To achieve this, we validated different criteria using a cohort of 812 cases with extracted pathological information [10, 11, 12, 13, 14, 15, 16, 18, 19, 22, 25, 27, 29, 30, 31, 32, 34, 35, 38, 39, 40, 42, 48, 49]. Our clinical experience‐informed validation yielded several key conclusions [46, 52, 53, 54]. Three categories of cases can be confidently diagnosed as MPLC using HPE alone: MLCs with distinct pathological types (e.g., one lesion identified as lung adenocarcinoma (LUAD) and another diagnosed as lung squamous cell carcinoma (LUSC)) are highly likely to be MPLC; MLCs predominantly composed of low‐grade components are highly likely to be MPLC; multifocal invasive mucinous adenocarcinomas (IMA) are highly likely to represent IPM. Conversely, two categories strongly warrant molecular evaluation. First, cases with high‐grade components require caution, as highlighted by the misclassification of 22 out of 93 similar cases, with 7 reclassified as IPM and 15 as MPLC. Second, cases diagnosed as IPM using HPE, compared to those diagnosed as MPLC, have a higher potential for misdiagnosis (MPLC: 69/284 vs. IPM: 96/207, *p* < 0.0001).

These conclusions offer valuable insights for developing a comprehensive diagnostic framework for suspected MLCs. This stepwise workflow would commence with clinical suspicion of MLCs, followed by pathological screening of surgical specimens. HPE results would then be used to stratify cases into low and high risks of misdiagnosis. Based on this stratification, tailored recommendations for concurrent molecular testing would be provided. This framework aims to guide personalized treatment strategies for patients with suspected MLCs, balancing diagnostic precision with cost‐effectiveness.

## 4 Discussion

This systematic review and meta‐analysis provide the first comprehensive evaluation of molecular and clinical‐pathological methods in distinguishing MLCs. We included 41 studies with 1266 patients across diverse clinical settings. Mole1 and Mole2, representing the two most typical and widely used molecular methods, were analyzed alongside the traditional clinical‐pathological approaches. Despite variability in panel sizes, Mole2 exhibited superior specificity and comparable sensitivity compared to Mole1. Comparisons between molecular methods and clinical‐pathological evaluations showed that molecular approaches exhibited significantly higher accuracy in MLCs diagnosis. While some heterogeneity existed among the included studies, these findings lay the groundwork for refining molecular diagnostic strategies and developing integrated clinical‐pathological –molecular diagnostic approaches to improve the management of MLCs.

Mole1 demonstrated robust prognostic stratification capabilities with consistent performance across various panels. However, Mole2 showed less favorable results for OS stratification, contrasting with other analyzes suggesting its superior performance. This discrepancy is likely due to the limited availability of prognostic data. Only three studies provided extractable prognostic data for Mole2, and these studies had relatively short follow‐up periods (e.g., Chang et al.'s study reported a median follow‐up of only 15 months [14]). This suggests that the observed suboptimal performance of Mole2 may not be an inherent limitation of the methodology, but rather a reflection of the need for further validation through large‐scale studies with longer follow‐up periods.

Our study reveals that traditional clinical‐pathological evaluations, once considered the gold standard for diagnosis, fall short in accuracy. We identified several reasons contributing to their suboptimal performance. First, the clinical diagnostic criteria used in the included studies lacked standardization, resulting in inconsistencies after data integration, even after data harmonization efforts guided by later‐published guidelines. Second, only 29.3% (227/774) of cases incorporated analyzes of histological subtypes. The limited adoption of CHA has led to misclassification, particularly in cases with overlapping or ambiguous features.

A combined approach integrating molecular assessments and clinical‐pathological criteria represents a pragmatic solution, especially in settings with limited access to molecular diagnostics. HPE can function as an effective initial triage tool for cases with clear diagnostic features, such as those characterized by distinct pathological features identifiable through specific criteria outlined earlier. In these cases, molecular diagnostics can be strategically reserved for more complex or ambiguous cases that necessitate higher diagnostic precision. This integrated strategy optimizes cost‐effectiveness, feasibility, and diagnostic yield, ensuring high accuracy while minimizing resource waste.

To address the challenge posed by the lack of a definitive reference diagnosis, we employed a Bayesian modeling approach to conduct diagnostic tests. Given the limited availability of prior information from real MLC cases and the potential biases introduced by simulated data, we performed additional model simulations under mild assumptions. These simulations were designed to avoid extremes, neither assuming a complete absence of prior knowledge nor imposing overly strong priors. The results obtained from this strategy were consistent with our primary findings, as shown in Figures S5 and S6.

This study has several limitations. First, the distribution of sequencing panels across studies was uneven, with a notable scarcity of data available for larger panels, particularly those comprising over 500 genes. While subsampling partially addressed this issue, it could not fully mitigate this limitation. Second, variations in bioinformatics analysis pipelines for NGS, such as variant allele frequency (VAF) detection thresholds and mutation calling protocols (Table S3), introduced potential inconsistencies that may have impacted the study's findings. Third, the prognostic analysis was constrained by the limited number of studies reporting prognostic data and small sample sizes, resulting in considerable uncertainty in effect size estimation. Additionally, genomic analyzes exploring mechanistic differences between MPLC and IPM were constrained by data format inconsistencies, incomplete datasets, and heterogeneity in panel designs and inclusion criteria. Preliminary findings did not reveal substantial differences in driver mutations or pathway‐level mutation distributions. Consequently, the main text focuses on results related to diagnostic performance, with mechanistic insights requiring further investigation in future studies.

## 5 Conclusions

NGS‐based molecular assessments demonstrate superior diagnostic accuracy compared to clinical‐pathological evaluations in distinguishing MPLC from IPM. Bioinformatics‐assisted molecular methods offer higher specificity and stability across sequencing panels than routine molecular assessments. Integrating molecular assessment with clinical‐pathological criteria can streamline the diagnostic process, leading to improved efficiency and cost‐effectiveness.

## 6 Author Contributions

Ziyang Wang, Xiaoqiu Yuan, Kezhong Chen: conceptualization. Ziyang Wang, Xiaoqiu Yuan: data curation. Ziyang Wang, Xiaoqiu Yuan: formal analysis. Kezhong Chen: funding acquisition. All authors: investigation. Ziyang Wang, Xiaoqiu Yuan, Kezhong Chen: methodology. Ziyang Wang, Xiaoqiu Yuan: project administration. Ziyang Wang, Xiaoqiu Yuan: resources. Ziyang Wang, Xiaoqiu Yuan: software. Ziyang Wang, Xiaoqiu Yuan, Yuntao Nie, Kezhong Chen: supervision. All authors: validation. Ziyang Wang, Xiaoqiu Yuan: visualization. Ziyang Wang, Xiaoqiu Yuan: writing – original draft. All authors: writing – review and editing.

## 8 Conflicts of Interest

The authors declare no conflicts of interest.

9

## Supporting information


**Data S1.** Supporting Information.

## Data Availability

The data are available from the corresponding author on reasonable request.
