# Supplementary material for: Next‐Generation Sequencing vs. Clinical‐Pathological Assessment in Diagnosis of Multiple Lung Cancers: A Systematic Review and Meta‐Analysis
Source: Thorac Cancer. 2025 Mar 21;16(6):e70039. doi: 10.1111/1759-7714.70039 (PMC11928291; doi:10.1111/1759-7714.70039)
Supplement: Supplementary file 1 — Data S1. Supporting Information. [file TCA-16-e70039-s001.docx]

**Supplemental Online Content**

Ziyang Wang, Xiaoqiu Yuan, Yuntao Nie, Jun Wang, Guanchao Jiang, Kezhong Chen

[Supplement Appendix1. Major Diagnostic Criteria for MPLC and IPM^(1-4)^ 2](#_Toc191422872)

[Supplement Appendix2. Mutation-Based Analysis of Clonal Relatedness 4](#_Toc191422873)

[Supplement Appendix3. Search Strategy 6](#_Toc191422874)

[Supplement Appendix4. Supplementary Methods 7](#_Toc191422875)

[Figure S1. Quality Assessment of Diagnostic Accuracy Studies 2 (QUADAS-2) Study Quality Summary 10](#_Toc191422876)

[Figure S2. Subgroup analysis of sequencing panel sizes 11](#_Toc191422877)

[Figure S3. Comparative diagnostic conclusiveness rates of two molecular evaluation methods across varying panel sizes 12](#_Toc191422878)

[Figure S4. Concordance analysis between the two molecular methods across different panel sizes and subsampling scenarios 13](#_Toc191422879)

[Figure S5. ROC curves for Mole1 and Mole2 across different panel sizes and subsampling scenarios 14](#_Toc191422880)

[Figure S6. Forest plots depicting the sensitivity and specificity analysis of Mole1 and Mole2, based on a Bayesian model with Beta (2, 2) prior assumptions, across various sequencing panel groups and subsampling scenarios 15](#_Toc191422881)

[Table S1: Subsampled Panels Used in Analysis 16](#_Toc191422882)

[Table S2: Prior Probability Distributions and Optimized Parameters for Sensitivity and Specificity Across Different Panels 41](#_Toc191422883)

[Table S3. Characteristics of Included Studies not shown in the main table 43](#_Toc191422884)

[Table S4. Diagnostic results of included cases under different methods 46](#_Toc191422885)

[Reference 77](#_Toc191422886)

This supplemental material has been provided by the authors to give readers additional information about their work.

# Supplement Appendix1. Major Diagnostic Criteria for MPLC and IPM^(1-4)^

|  | Martini Criteria (1975) | ACCP Guidelines (2007 & 2013) | IASLC Recommendations (2016) | | | |
| --- | --- | --- | --- | --- | --- | --- |
|  |  |  | Clinical Criteria | | Pathological Criteria | |
|  |  |  | Exact Evidence | Suggestive Evidence | Exact Evidence | Suggestive Evidence |
| MPLC | Different histological types, Same histological type with in situ origin, no lymph node metastasis, no extrapulmonary metastasis, tumor-free interval ≥ 2 years | Different histological subtypes, Different in situ origins, Different genetic characteristics, Same histological type with no N2, N3 lymph node metastasis, no extrapulmonary metastasis, tumor-free interval ≥ 4 years | Markedly different histological types based on biopsy | Different genetic characteristics (driver gene mutations),  No lymph node and distant metastasis, Different radiological features or metabolic uptake, Different growth rates (if previous imaging data is available) | Markedly different histological types, markedly different comprehensive pathological evaluation results, Squamous cell carcinoma originating from in situ carcinoma | Different genetic characteristics (driver gene mutations),  No lymph node and distant metastasis |
| IPM | —— | Same histological type with distant metastasis, Same histological type with N2, N3 lymph node metastasis | Identical chromosomal breakpoints (comparative genomic hybridization) | Same genetic characteristics (when histological types are the same),  Presence of lymph node and distant metastasis,  Similar radiological features.  Similar growth rates (if previous imaging data is available) | Identical chromosomal breakpoints (comparative genomic hybridization) | Same genetic characteristics, Presence of lymph node and distant metastasis, Identical comprehensive pathological evaluation |

The protocol of CHA generally adheres to the WHO guidelines for lung adenocarcinoma classification, involving the recording of histological subtypes in 5% increment, along with other histological features of the tumor, such as cytology (clear cells or signet ring features) or stroma (fibroblastic proliferation or inflammation)(5-7).

# Supplement Appendix2. Mutation-Based Analysis of Clonal Relatedness

We compared two representative methods for assessing clonal relatedness based on mutation analysis: counting shared mutations (Mole1) and calculating clonal probability using all mutations (Mole2). Mole1 is the more commonly used approach.

Both Mole1 and Mole2 can be applied to patients with multiple lung cancers who have undergone molecular testing targeting single nucleotide variants (SNVs), such as next-generation sequencing (NGS). For Mole1 assessment, standard bioinformatic analysis should be performed to generate a clinician-interpretable mutation format (e.g., EGFR 19del). Mole2 assessment requires technical specialized personnel to encode mutations according to the input requirements of the clonality analysis software.

Mole1 embodies an empirical approach prevalent in clinical practice, wherein MPLC is defined by the absence of shared mutations between paired tumors, or the presence of private mutations unique to each tumor, with the exception of a single shared hotspot mutation. Cases lacking detectable mutations or exhibiting a single overlapping hotspot mutation are classified as "inconclusive (IN)." All other scenarios are classified as IPM. However, the definitions of hotspot mutations vary across studies and lack standardization. To address this, we adopted the COSMIC database's designation of hotspot mutations as a reference standard in this study.

Mole2 employs a bioinformatics-assisted approach via the "Clonality" package(8), which estimates clonal relatedness by assessing mutation frequencies and mutation patterns between tumors. This package has been widely used for analyzing tumor clonal relationships and is therefore considered representative. We utilized TCGA data as the reference dataset, considering that the included studies involved a diverse, multi-ethnic population. Clonal relatedness is assessed based on the presence of unique variants (indicating independent origins) and shared mutations (indicating a common origin). To address inter-study heterogeneity, we utilized the SNVtest function. Extracted mutations were re-annotated with “Transvar” to meet requirements of SNVtest function(9). The failure rate of re-annotation is below 2%. SNVtest assigns a p-value to each tumor pair. A p-value below 0.05 indicates IPM, while a p-value above 0.95 confirms MPLC. Cases with p-values between 0.05 and 0.95 remain inconclusive. For patients with three tumors, pairwise comparisons were conducted, and IPM was diagnosed if any pair was classified as IPM.

To assess the performance of molecular assessments across different sequencing panels, we employed two complementary approaches.

1. Panel-Grouped Analyses: Initially, we categorized panels based on size, using thresholds of 30 and 100 genes. For more granular analyses, we further subdivided panels into the following groups: 1, 2, 8-10, 20-35, 47-53, 168, 400-464, and 500-10000 genes. While this method provides a straightforward comparison, it is limited by the relatively small number of panel types and cases within each group.
2. Subsampling Analyses: To overcome the limitations of panel grouping, we implemented a mutation subsampling approach. This method simulates molecular evaluations under various panel sizes by randomly selecting subsets of mutations from larger panels. While this approach addresses the limitations of panel grouping, it introduces potential variability due to differences in sequencing parameters (e.g., VAF thresholds, sequencing depth) across studies. The impact of these parameters on evaluation results remains to be fully elucidated, and therefore, findings from subsampling analyses should be interpreted with caution.

Details regarding the composition of the subsampled panels are provided in Table S1. These panels were constructed by selecting mutations based on their ranked frequency of detection, referencing commonly used commercial panels. This approach ensured that larger panels comprehensively covered the mutational landscape of smaller panels, thereby validating the feasibility and reliability of the subsampling process.

# Supplement Appendix3. Search Strategy

This study followed the Preferred Reporting Items for Systematic Reviews and Meta-Analyses (PRISMA) guidelines to conduct a meta-analysis of diagnostic test accuracy(10). The protocol was pre-registered on PROSPERO (CRD42024612366).

A comprehensive search was performed across four major databases, including PubMed, Web of Science, Embase, and Scopus, to identify studies on molecular testing in MLCs. Considering the widespread acceptance of HPE and the large-scale clinical use of NGS since 2010, the study selection period was set from 2010 to March, 2024. The search limited to original studies published in English. The search terms included combinations of keywords such as “Diagnosis”, “Multiple”, “Second”, “Lung Neoplasms”, and “High-Throughput Nucleotide Sequencing”.

Studies lacking extractable molecular testing data (gene mutations) were excluded, as the primary aim of this review is to compare the performance of various molecular evaluation methods. To mitigate concerns about publication bias and inflated effect estimates from small studies, we further excluded studies with fewer than 8 patients. Review articles, commentary articles, and case reports were excluded. Additionally, we screened the reference lists of all included studies to identify additional relevant studies for inclusion in the meta-analysis.

The search strategy included the following keywords and terms across multiple databases:

| **PubMed** | (“Diagnosis”[Mesh]) AND (((second[Title]) OR (multiple[Title])) AND (“Lung Neoplasms”[Mesh]))  (“High-Throughput Nucleotide Sequencing”[Mesh]) AND (((second[Title/Abstract]) OR (multiple[Title/Abstract])) AND (“Lung Neoplasms”[Mesh])) |
| --- | --- |
| **Web of Science** | TS=(lung cancer) AND (TI=(multiple) OR TI=(second)) AND TS=(diagnosis) |
| **Embase** | diagnosis:ab,ti AND ‘multiple pulmonary nodules’:ab,ti AND english:la AND [2010-2024]/py |
| **Scopus** | (TITLE (lung) AND TITLE-ABS-KEY (ngs) AND TITLE-ABS-KEY (multiple)) AND PUBYEAR > 2009 AND PUBYEAR < 2025 AND (LIMIT-TO (LANGUAGE, “English”)) AND (LIMIT-TO (DOCTYPE, “ar”)) |

# Supplement Appendix4. Supplementary Methods

**Search Strategy and Review Process**

Two authors (Z.Y.W and X.Q.Y) independently conducted the literature screening. Initial screening was based on titles and abstracts, followed by a full-text review to determine eligibility. For each included study, the following data were extracted: author, publication year, study location, study design, sample size, patient demographics, diagnostic methods (e.g., NGS panel size and clinical or pathological standards), the discriminating criteria for MLCs, primary outcomes (diagnostic accuracy and prognostic indicators), and available genomic alterations at various levels.

The identification results based on clinical-pathological criteria included different kinds of HPE (CHA is separately annotated), the ACCP guidelines, the IASLC proposal and the Martini-Melamed criteria. Any disagreements during the screening process were resolved by a third author. For studies with missing key data, the original authors were contacted to request additional information.

**Quality Assessment**

Two authors (Z.Y.W and Y.T.N) independently assessed the risk of bias in each study using the Quality Assessment of Diagnostic Accuracy Studies 2 (QUADAS-2) tool. Any disagreements were resolved through discussion with the third author (Y.T.N). The QUADAS-2 tool evaluates four domains of potential bias: patient selection, index test, reference test, and flow and timing. Additionally, it assesses the applicability of the study results to the target population, identifying risks of reduced generalizability due to bias. Studies deemed to have a high risk of bias were flagged, and the reasons for this designation were documented. However, if the bias affected only specific parts of the analysis, the study was included while acknowledging the potential limitations. The result was shown as **Figure S1**.

**Bayesian Latent Class Model for Evaluating Diagnostic Performance**

A Bayesian latent class model (LCM) was utilized to evaluate the diagnostic performance of different tests in the absence of a perfect reference standard, particularly for molecular methods in diagnosing MLCs(11, 12). This model addressed the limitations of imperfect reference tests by estimating the true disease status of each participant based on the combined results of both index and reference tests. By not assuming that the reference test is perfect, the model accounted for potential misclassifications, such as false positives and false negatives, which are common challenges in MLCs diagnosis.

The disease prevalence, sensitivity, and specificity of the tests were modeled as random variables using prior distributions, reflecting prior knowledge while allowing for uncertainty in parameter estimates. The setting of prior distributions is detailed later. To address conditional dependencies between the index and reference tests, the model included covariance terms for sensitivity and specificity, capturing the degree of dependence between tests and adjusting the estimates accordingly.

The likelihood of observed test outcomes was modeled using a multinomial distribution, with probabilities based on true disease status and test parameters. Diagnostic accuracy was assessed using two primary metrics: the improvement in discrimination index (IDI) and the area under the receiver operating characteristic curve (AUC). The IDI measured improvements in the classification accuracy of the new molecular approach (Mole2) compared to the traditional approach (Mole1), while the AUC quantified overall diagnostic performance, with AUC differences (AUC.diff) highlighting the improvement from new molecular methods.

The Bayesian model was implemented in OpenBUGS (version 3.2.3), using Markov Chain Monte Carlo (MCMC) simulations to estimate posterior distributions. After a burn-in phase of 5,000 iterations, 50,000 sampling iterations were used to ensure stable estimates, with convergence confirmed using the Gelman-Rubin diagnostic. Posterior means and 95% credible intervals were reported for key parameters, providing robust estimates of disease prevalence, sensitivity, specificity, and diagnostic performance metrics.

By modeling the imperfections of reference standards, addressing conditional dependencies, and accounting for heterogeneity, this Bayesian latent class model provided a comprehensive and robust framework for evaluating diagnostic tests in the complex context of MLCs diagnosis.

**Prior Probability Distribution for Simulated MLCs**

Due to the lack of validated MLCs cohorts, we constructed a simulated dataset to model MPLC and IPM using pairwise sequencing profiles derived from multi-region sequencing of solitary NSCLC(13). Samples from the same patient were paired to simulate IPM (sim-IPM), while samples from different patients were paired to mimic MPLC (sim-MPLC), generating 28,203 simulated cases. WES data from these cases were subsampled to evaluate the performance of two molecular methods. Optimized parameters for Clonality corresponding to each panel were established using a subset of these cases, applied to construct ROC curves, calculate AUCs, and determine probability cutoffs, with repeated iterations ensuring robustness and validation in further analyses.

Based on these simulations, we derived prior probability distributions for several key parameters of Mole1 and Mole2, including sensitivity, specificity, and their 95% credible intervals (**Table S2**). Due to significant heterogeneity in these parameters across different panels, targeted parameter estimation was performed for each scenario(14).

Given the potential discrepancies between simulated MLCs and real-world scenarios, we also conducted model simulations under mild assumptions to account for uncertainty. Specifically, we adopted a low-information beta (2, 2) prior distribution, avoiding assumptions of either complete ignorance or overly strong prior knowledge. This low-information prior remained unchanged across subgroups.

**Clinical-pathological Review of Included Cases and Prognostic stratification** **Analysis**

For cases with incomplete clinical evaluations, those meeting the basic requirement for clinical evaluation were manually reviewed by two authors (Z.Y.W and X.Q.Y) with follow-up data unprovided. Due to the variability and subjectivity of pathological evaluation standards across studies, no re-evaluation was performed.

For the prognostic analysis, hazard ratios (HRs) and 95% confidence intervals were calculated to evaluate survival outcomes. When HRs were not directly reported, they were derived from Kaplan-Meier survival curves using the “Engauge Digitizer” software (https://markummitchell.github.io/engauge-digitizer) and standard estimation methods(15). A random-effects model, based on the DerSimonian and Laird method, was employed to pool HRs and CIs, accounting for heterogeneity across studies. Prognostic outcomes, including overall survival (OS) and disease-free survival (DFS), were analyzed across different molecular method subgroups and panel size subgroups to ensure the robustness of the findings. A random-effects model was chosen to account for clinical heterogeneity potentially present across studies. Heterogeneity among studies was evaluated using the chi-squared test and I^2^ statistics. The interpretation of I^2^ values was as follows: 0%–24%, no heterogeneity; 25%–49%, low heterogeneity; 50%–74%, moderate heterogeneity; and 75%–100%, high heterogeneity. All data analyses were performed using R software (version 4.3.2 for macOS and version 4.4.1 for Windows; <https://www.r-project.org/>).

# Figure S1. Quality Assessment of Diagnostic Accuracy Studies 2 (QUADAS-2) Study Quality Summary


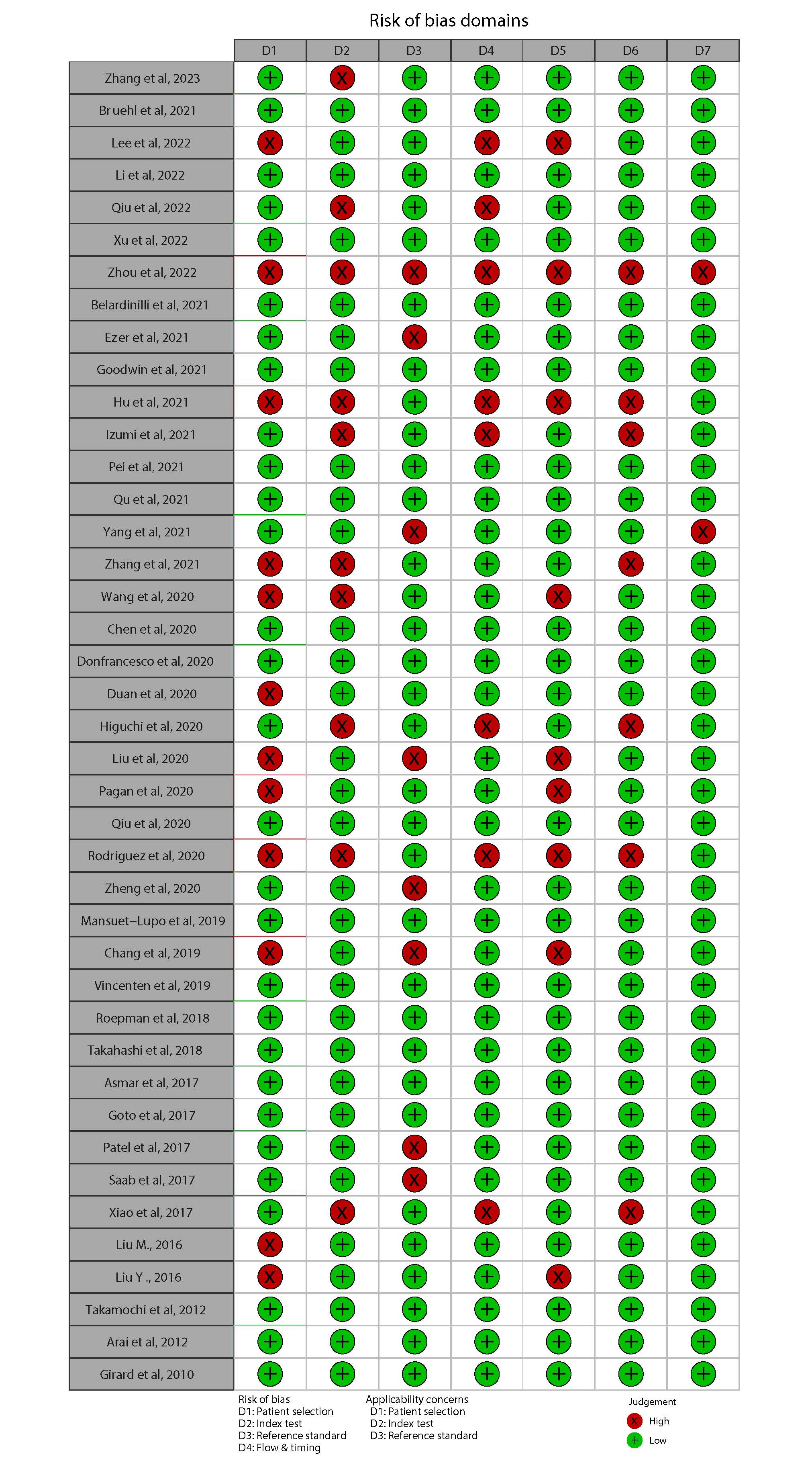


# Figure S2. Subgroup analysis of sequencing panel sizes

**
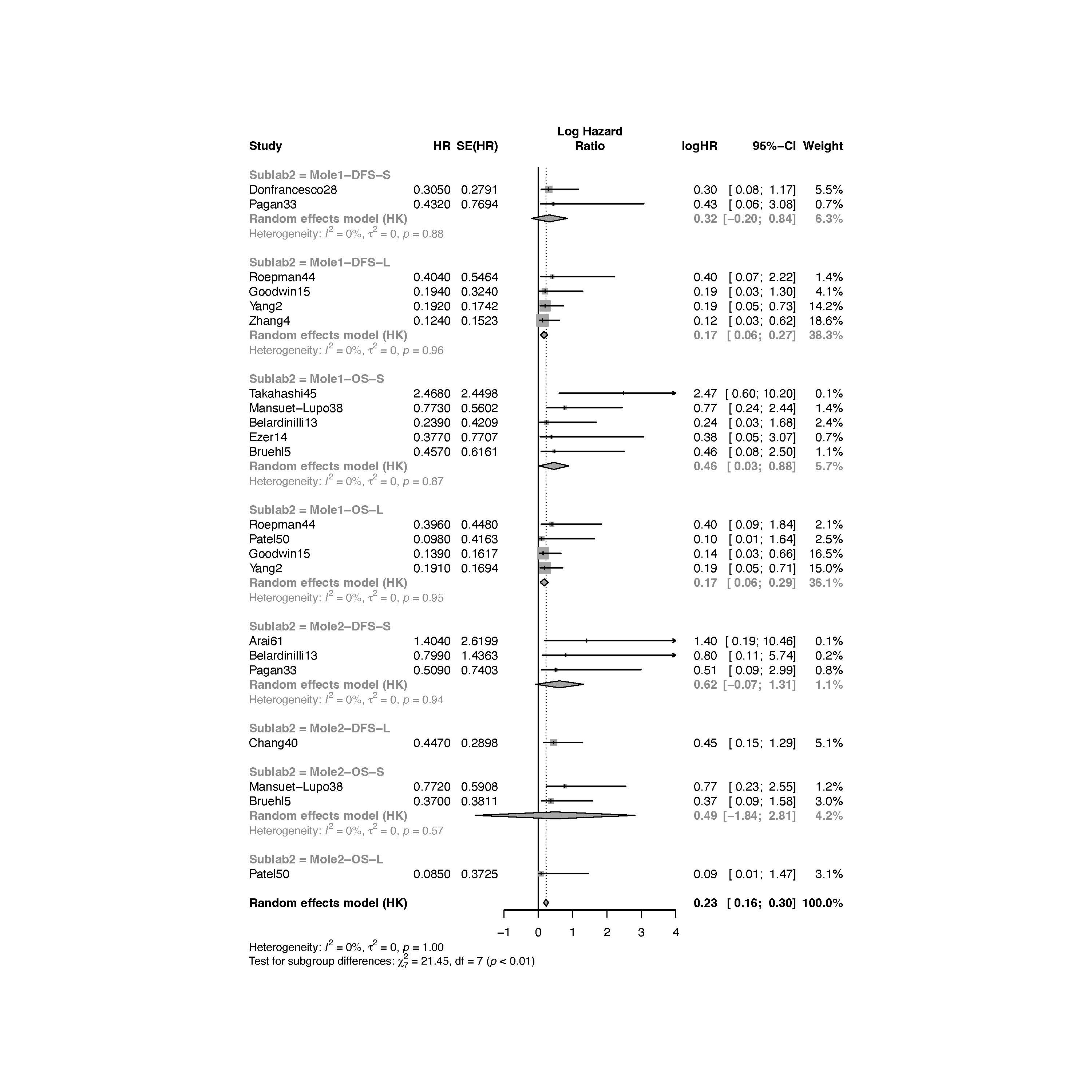
**

# Figure S3. Comparative diagnostic conclusiveness rates of two molecular evaluation methods across varying panel sizes


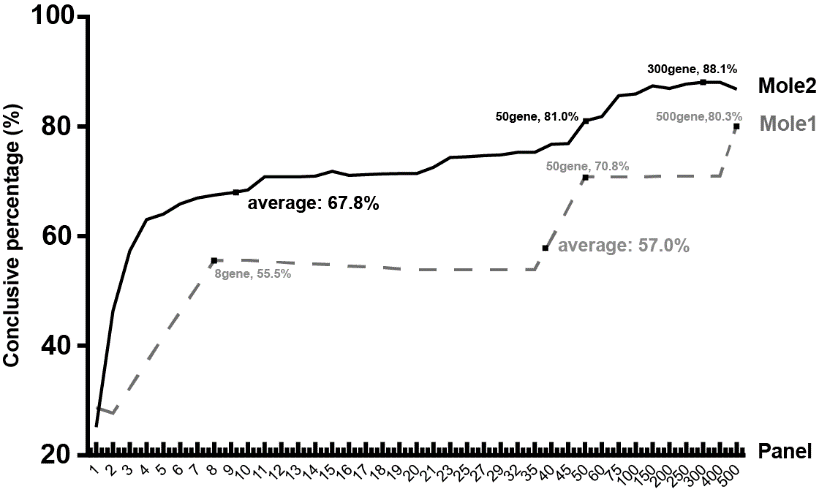


The figure depicts the proportion of conclusive diagnostic outcomes (defined as samples receiving definitive classification, %) across sequencing panels of varying sizes (number of targeted genes). Two molecular methods are compared: Mole1 (dashed line), which classifies samples by counting shared mutations, and Mole2 (solid line), which calculates clonal probability using all mutation data. Despite both methods exhibiting improved diagnostic conclusiveness with larger panels, Mole2 consistently demonstrates superior performance in achieving definitive classifications as panel size increases.

# Figure S4. Concordance analysis between the two molecular methods across different panel sizes and subsampling scenarios

**
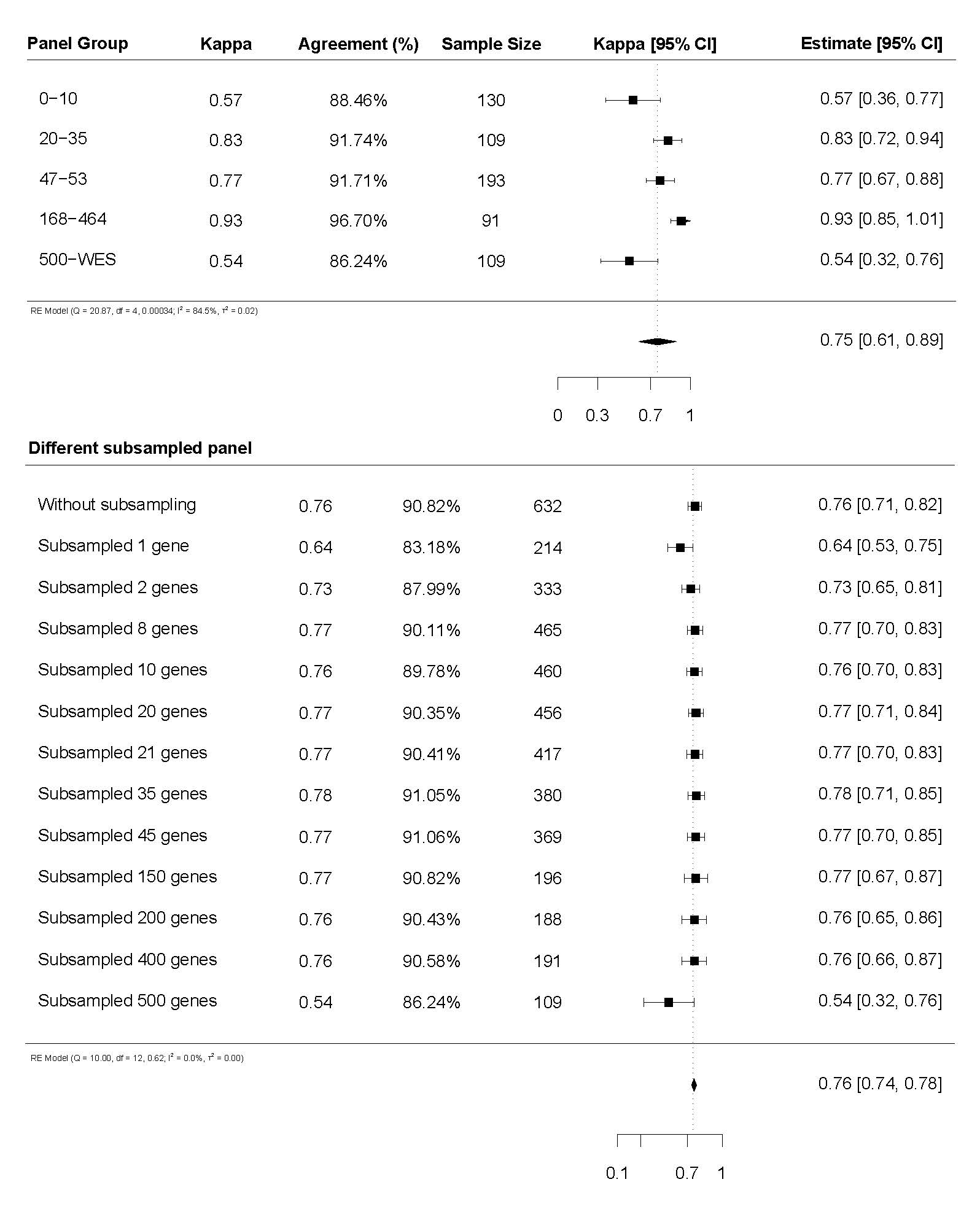
**

The figure illustrates Kappa values, agreement percentages, and sample sizes for each group, showing the stability of molecular assessment outcomes across various panel configurations.

# Figure S5. ROC curves for Mole1 and Mole2 across different panel sizes and subsampling scenarios

**
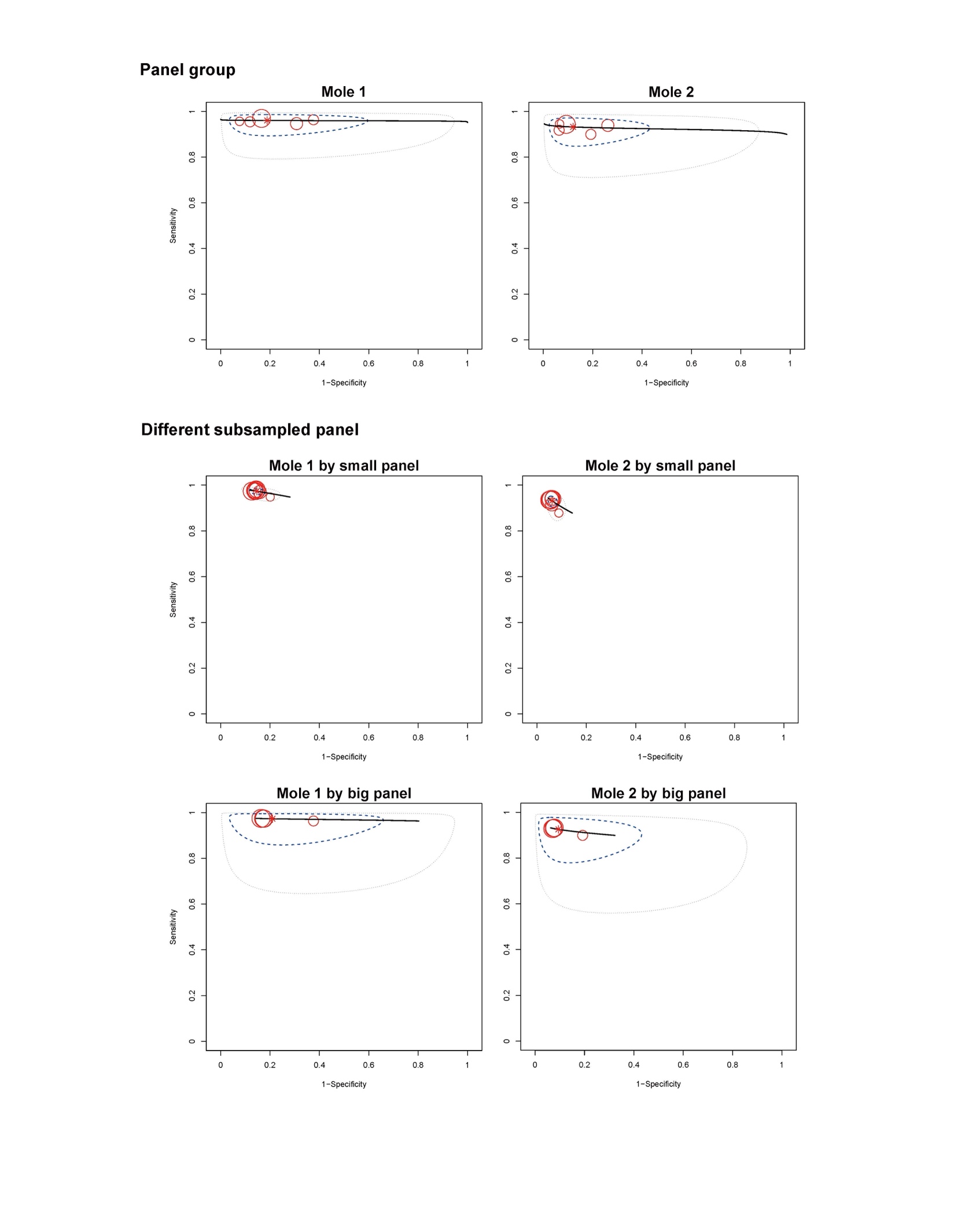
**

The figure shows the diagnostic performance of each method, highlighting that Mole2 consistently achieves better stability compared to Mole1, particularly with small panels.

# Figure S6. Forest plots depicting the sensitivity and specificity analysis of Mole1 and Mole2, based on a Bayesian model with Beta (2, 2) prior assumptions, across various sequencing panel groups and subsampling scenarios

**
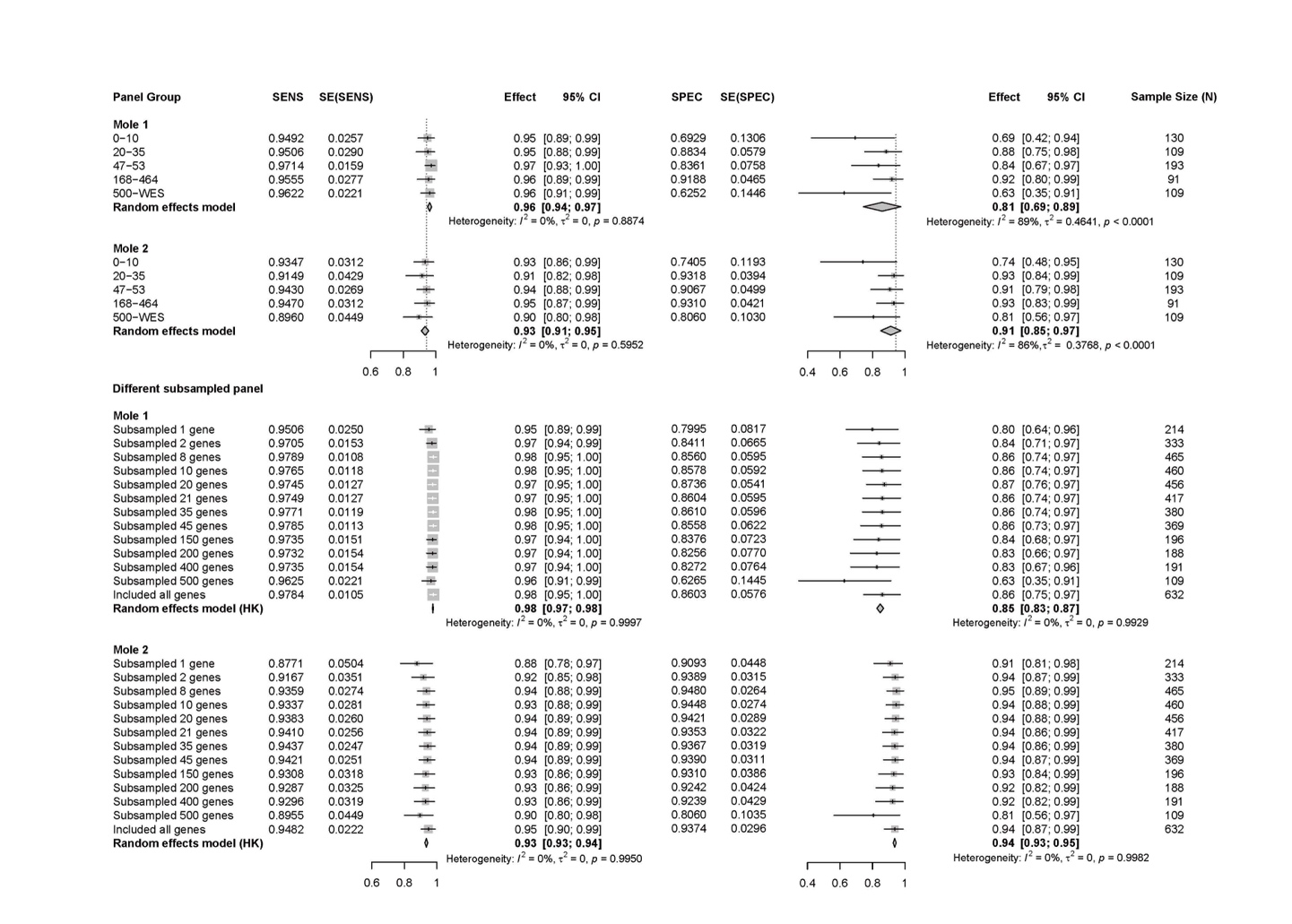
**

# Table S1: Subsampled Panels Used in Analysis

| **Panel** | **Case number** | **Coverage** |
| --- | --- | --- |
| WES | 25 | AASDH, AATF, ABCA9, ABCB5, ABCC1, ABCD4, ABI3BP, ABL1, ACACB, ACAN, ACSBG1, ACTG2, ADAM2, ADAM22, ADAM23, ADAM30, ADAMDEC1, ADAMTSL3, ADCY7, ADIPOR1, ADPRHL1, AEBP2, AGO2, AGPS, AHI1, AIM2, AKT1, AKT2, AKT3, ALDH1B1, ALG6, ALG9, ALK, AMER1, ANK2, ANKFY1, ANKHD1, ANKRD11, ANKRD30B, ANKS1B, ANXA1, ANXA7, AOAH, AP3B1, APC, APCDD1, APOB, AR, ARAF, ARAP3, ARFRP1, ARHGAP11A, ARHGAP35, ARHGEF25, ARID1A, ARID1B, ARID2, ARID5B, ARRDC3, ASB2, ASB3, ASTN2, ASXL1, ASXL2, ATAD5, ATF5, ATF7IP, ATG13, ATM, ATP2A2, ATP2B3, ATPAF1, ATR, ATRX, AURKA, AURKB, AXIN1, AXIN2, AXL, B4GALNT2, BAAT, BAP1, BBC3, BCCIP, BCL11A, BCL11B, BCL2, BCL2L11, BCL2L2, BCL6, BCOR, BCORL1, BCR, BECN1, BEND4, BIRC3, BLM, BMPR1A, BMPR2, BMS1, BRAF, BRCA1, BRCA2, BRD4, BRINP3, BRIP1, BRP44L, BRWD1, BTG1, BTK, BUB1, C10ORF140, C10ORF90, C11ORF41, C12ORF26, C12ORF51, C15ORF2, C15ORF44, C21ORF33, C2ORF71, C2ORF77, C3ORF19, C4ORF17, C6ORF168, C9ORF117, C9ORF79, C9ORF86, CABYR, CACNA1G, CACNA1I, CADM2, CADM3, CALCA, CALR, CAPZA3, CARD11, CARM1, CASP12, CASP8, CBFA2T2, CBL, CBLB, CCDC113, CCDC15, CCDC164, CCDC83, CCND1, CCND2, CCND3, CCNE1, CCNT2, CD19, CD1B, CD274, CD2AP, CD5L, CD74, CD74-NRG1, CD79B, CD9, CDC73, CDH18, CDH7, CDK12, CDK4, CDK6, CDK8, CDKN1A, CDKN1C, CDKN2A, CEBPA, CENPA, CENPF, CEP192, CEP350, CHD1, CHD2, CHD4, CHEK1, CHEK2, CHRM2, CHRNB3, CHST12, CHSY1, CIC, CLCN1, CLDND1, CLIP3, CNDP1, CNNM1, CNOT2, CNST, CNTN5, CNTN6, CNTNAP2, CNTNAP4, CNTNAP5, COBL, COG8, COL14A1, COL16A1, COL17A1, COL22A1, COL2A1, COL3A1, COL4A3, COL9A1, CORIN, CPEB4, CPS1, CPT1A, CPXM2, CREBBP, CRKL, CROCC, CSDE1, CSF1R, CSF3R, CSMD1, CSMD2, CSMD3, CTAGE10P, CTCF, CTDP1, CTLA4, CTNNB1, CTNND2, CUL3, CUL4A, CUL4B, CXCR4, CXORF22, CXORF26, CYBB, CYLD, CYP17A1, CYP4F2, CYSLTR2, DAG1, DAGLA, DAK, DAXX, DCAF8L2, DCHS2, DCST2, DCUN1D1, DDR2, DDX10, DDX27, DDX3X, DDX51, DEFB112, DENND1A, DENND3, DHODH, DICER1, DIDO1, DIS3, DMXL2, DNAH11, DNAH2, DNAH6, DNAH9, DNAJB1, DNAJB8, DNMT1, DNMT3A, DNMT3B, DOT1L, DPP10, DPP6, DPYD, DPYS, DROSHA, DSCAM, DSG1, DSG3, DTX1, DYNC1H1, DYNC2H1, DYNC2LI1, DZIP3, E2F3, EAPP, EDC4, EDN3, EFCAB5, EFR3A, EGER, EGFL7, EGFR, EHD1, EIF2B1, ELF3, ELMO1, ELMO2, EML4, EML5, EMR2, EMSY, EP300, EPAS1, EPB41L4B, EPC1, EPHA2, EPHA3, EPHA5, EPHA7, EPHB1, EPPK1, ERBB2, ERBB3, ERBB4, ERC2, ERCC1, ERCC2, ERCC3, ERCC5, ERCC6, ERG, ERRFI1, ESR1, ESR2, ETV1, ETV4, ETV6, EWSR1, EXT1, EXT2, EXTL3, EYA2, EYS, F11R-NRG1, FABP1, FAM114A2, FAM124B, FAM129B, FAM13C, FAM160B2, FAM193A, FAM47B, FAM58A, FAM65B, FAM71A, FAM76A, FANCA, FANCB, FANCC, FANCD2, FANCE, FANCG, FANCI, FANCM, FAR2, FAT1, FAT3, FAT4, FBXO34, FBXO38, FBXO39, FBXW7, FCGR2B, FGA, FGF12, FGF14, FGF19, FGF23, FGF3, FGF4, FGF6, FGFR1, FGFR2, FGFR3, FGFR4, FH, FHAD1, FIGN, FLCN, FLT1, FLT3, FLT4, FN1, FNIP1, FOXA2, FOXM1, FOXP1, FOXP2, FOXR2, FREM3, FRS2, FSTL5, FTL, FTSJD1, FUBP1, FYCO1, FYN, GABRB2, GABRB3, GABRG2, GABRG3, GALNT12, GALR3, GATA1, GATA2, GATA3, GATA4, GATA6, GBP7, GCC2, GDF11, GDF2, GGH, GKAP1, GLI1, GLIPR2, GML, GNAS, GNONES, GNPTAB, GOLGA3, GP5, GPC3, GPR75, GPR98, GPRIN2, GPX5, GRIA4, GRID1, GRIN2A, GSK3B, GSX1, GTF2B, GTF3C1, H3F3A, H3F3B, H3F3C, HAUS1, HDAC2, HDAC9, HEATR3, HEATR7B2, HEATR8, HELZ, HER2, HGF, HIF1A, HIF1AN, HIST1H1C, HIST1H1E, HIST1H2BD, HIST1H3B, HIST1H3D, HIST1H3G, HIST1H3I, HIST3H3, HIVEP1, HLA-A, HLA-B, HLTF, HMCN1, HMGA2, HNF1A, HRAS, HRASLS5, HSP90AA1, HTR1A, HTR2A, ICOSLG, ID3, IDH1, IDH2, IFI27L2, IFNGR1, IFNL2, IGF1, IGF1R, IGF2, IGFN1, IGSF5, IKBKE, IKZF1, IL22RA1, IL6R, IL7R, IMMP1L, INADL, INHA, INHBA, INPP4A, INPP4B, INPP5B, INPPL1, INSR, IPO11, IPO7, IRF4, IRS1, IRS2, ITGA10, ITGA11, ITGB4, ITIH2, ITLN2, ITM2B, ITSN2, JAK1, JAK2, JAK3, JAKMIP1, JMJD4, JUN, KANSL2, KAT6A, KAT6B,RP11-77G23.2, KDM4A, KDM5A, KDM5B, KDM5C, KDM6A, KDR, KEAP1, KEL, KIAA0232, KIAA1033, KIAA1107, KIAA1217, KIAA1429, KIAA1549, KIAA1614, KIAA1731, KIAA1967, KIF19, KIF24, KIF4A, KIF5B, KIT, KLF4, KLHL1, KLHL6, KLHL7, KMT2A, KMT2B, KMT2C, KMT2D, KMT2С, KNSTRN, KPRP, KRAS, KRT14, KRT28, KRTAP15-1, KRTAP27-1, KYNU, LACTB, LAMA4, LATS1, LATS2, LBP, LEF1, LENG8, LGI4, LILRB4, LIN54, LOC283116, LOC388499, LOXHD1, LPP, LRFN5, LRG1, LRIG2, LRIT3, LRP1B, LRP2, LRRC43, LRRC4C, LRRC72, LRRK2, LTBP1, LUM, LUZP2, LYN, LZTR1, MAGEA10, MAGED2, MAGI1, MAGI2, MALT1, MAMLD1, MAP1B, MAP23K1, MAP2K1, MAP2K2, MAP2K4, MAP3K1, MAP3K13, MAPKBP1, MARS, MASP1, MAX, MBD5, MCL1, MCM3AP, MCMBP, MDC1.00, MDM2, MDM4, MDN1, MED12, MED14, MED17, MED6, MEF2B, MEFV, MEN1, MET, MGA, MIB1, MICALL1, MIS18A, MITF, MKI67, MKRN3, MLANA, MLF1, MLH1, MLH3, MLL, MLL3, MLLT1, MMRN1, MMS22L, MOCS3, MORC1, MORC3, MORF4L1, MPL, MPP6, MRE11A, MRPL24, MS4A6A, MSH2, MSH3, MSH6, MSI2, MST1R, MTF1, MTFMT, MTOR, MTR, MUC16, MUC17, MYB, MYC, MYCBP2, MYCN, MYEOV2, MYF5, MYH15, MYH16, MYH4, MYO10, MYO18B, MYO1A, MYO1B, MYO3A, MYO5B, MYOD1, NAA50, NAB2, NAE1, NANP, NAV2, NAV3, NBEA, NBN, NCAN, NCKAP1, NCOA1, NCOA3, NCOR1, NEGR1, NEK11, NETO2, NEUROD1, NEUROD6, NF1, NF2, NFE2L2, NFIX, NFKBIA, NINL, NKX2-1, NLGN1, NLRP13, NMD3, NMUR2, NOBOX, NOS1, NOTCH1, NOTCH2, NOTCH3, NOTCH3-CNTNAP4, NOTCH4, NPM1, NPY1R, NR4A3, NRAS, NRG1, NRK, NSD1, NTHL1, NTRK1, NTRK2, NTRK3, NUF2, NUMA1, NUP153, NUP93, OBSCN, ODZ3, OPN4, OR10G7, OR10G8, OR10H4, OR10K2, OR11L1, OR1A1, OR2AK2, OR2L3, OR2L8, OR2M7, OR2W5, OR4N2, OR51E2, OR51L1, OR5AP2, OR5D14, OR5D18, OR5L1, OR5T1, OR5T2, OR8J1, ORC5, OTOG, PAK1, PAK3, PAK7, PALB2, PAPPA, PAPPA2, PARK2, PARP1, PARP2, PAX3, PAX5, PBRM1, PCDH18, PCDHA13, PCDHA7, PCDHB10, PCDHB11, PCDHB13, PCDHB14, PCDHGB3, PCLO, PCNT, PDCD1, PDCD11, PDE6A, PDGFRA, PDGFRB, PDHA2, PDPK1, PDYN, PDZD2, PELI3, PER1, PER3, PET112, PEX19, PGAM5, PGBD2, PGR, PHF15, PHF23, PHF8, PHOX2B, PIBF1, PIEZO2, PIF1, PIGC, PIGR, PIK3C2A, PIK3C2B, PIK3C2G, PIK3C3, PIK3CA, PIK3CD, PIK3CG, PIK3R1, PIK3R2, PIK3СА, PIM1, PKM2, PLA2G15, PLCB4, PLCG2, PLCH1, PLEKHA7, PLS3, PMS1, PMS2, PNCK, POLD1, POLE, POM121L12, POM121L2, POT1, POTEA, POU5F2, PPARG, PPFIA2, PPM1D, PPP2CB, PPP2R1A, PPP2R2A, PPP4R1, PPP4R2, PPP6C, PRDM1, PRDM10, PREX2, PRKAG3, PRKCA, PRKCI, PRKD1, PRKDC, PRKN, PRMT10, PRR25, PRSS1, PRSS35, PRTG, PTBP3, PTCH1, PTEN, PTK2, PTK7, PTN, PTPN11, PTPRB, PTPRD, PTPRN2, PTPRS, PTPRT, PTPRZ1, PWP2, PXDNL, PZP, RAB3GAP2, RABEP2, RAC2, RAD21, RAD50, RAD51C, RAD52, RAD54L, RAF1, RALGAPA2, RANBP2, RAP2A, RASA1, RB1, RBM10, RBM15, RCL1, RCN1, RECQL, RECQL4, REL, RELN, RET, RHBDF2, RICTOR, RIF1, RIMS2, RIT1, RLIM, RNF123, RNF157, RNF43, ROBO2, ROBO3, ROS1, RP1L1, RPL3, RPL5, RPS26, RPS6KA1, RPS6KA4, RPTOR, RRAS, RRAS2, RSF1, RUNX1T1, RUSC2, RXRA, RYR2, S1PR3, SAMD4A, SATL1, SBDS, SCAF11, SCN10A, SCN3A, SCN9A, SDHA, SDHB, SDHC, SDK1, SDR42E1, SEMA3A, SEMA3E, SEMA6A, SERPINA11, SERPINB4, SERPINI2, SESN1, SETBP1, SETD1A, SETD2, SF3B1, SGIP1, SH2B3, SHOC2, SHQ1, SI, SIPA1L2, SLC16A7, SLC22A2, SLC22A24, SLC25A34, SLC27A3, SLC29A1, SLC34A2, SLC38A4, SLC4A7, SLC5A3, SLC6A17, SLC8A3, SLIT2, SLITRK1, SLITRK2, SLITRK3, SLITRK4, SLX4, SMAD2, SMAD3, SMAD4, SMARCA1, SMARCA4, SMARCB1, SMCR7L, SMG7, SMO, SMYD1, SNAP25, SNCAIP, SORCS2, SOS1, SOX17, SOX2, SOX4, SOX6, SOX9, SPAG5, SPATA16, SPATA4, SPATS2, SPEF2, SPEG, SPEN, SPET9, SPG11, SPNS3, SPOP, SPTA1, SPTBN1, SPTLC3, SPZ1, SRC, SRRM2, SRSF2, SRSF5, SS18, SST, STAG2, STAM, STAT3, STAT5B, STK11, STRN, STXBP5L, SUZ12, SVIL, SWT1, SYCP2, SYK, SYNE1, SYNPO2, TAF1, TAF6, TAP1, TBC1D22B, TBC1D23, TBC1D4, TBX3, TCF3, TCF4, TCF7L2, TCHHL1, TCP11L2, TCRBV7S1A1N2T, TECTA, TEK, TERT, TET1, TET2, TGFBR1, TGFBR2, TGM6, TIMP4, TKTL2, TLR4, TM9SF4, TMCO3, TMCO7, TMEM100, TMPRSS11B, TMTC3, TNFAIP3, TNFRSF10D, TNFRSF14, TNIK, TNKS2, TNR, TNRC6A, TNRC6B, TOP1.00, TOP1MT, TP53BP1, TP63, TPMT, TPRA1, TPTE, TRAF7, TRANK1, TRIM29, TRIM58, TRIML1, TRIP12, TROAP, TRPA1, TRPC6, TSC1, TSC2, TSHR, TSHZ2, TSKU, TSPAN31, TSPEAR, TTC23, TTN, TUBAIA, TUBB8, TXLNB, U2AF1, UBP1, UBR2, UGT1A1, UNC79, UNC80, UPB1, UPF1, USP15, USP24, USP34, USP48, USP9X, VAV1, VEGFA, VEZF1, VHL, VIL1, VPRBP, VTCN1, VWA3A, VWF, WAS, WASL, WDFY4, WDR43, WDR49, WDR64, WDR72, WDR81, WDR83, WHSC1, WHSC1L1, WISP3, WNT10B, WRAP53, WRN, WT1, WTAP, XIAP, XIRP2, XKR4, XPO1, XPO6, XRCC1, ZAK, ZBED5, ZBP1, ZBTB16, ZBTB8A, ZDHHC11, ZER1, ZFAT, ZFHX3, ZFHX4, ZFPM2, ZIC4, ZNF14, ZNF160, ZNF212, ZNF257, ZNF367, ZNF426, ZNF462, ZNF534, ZNF555, ZNF681, ZNF683, ZNF703, ZNF814, ZNF831, ZNF99, ZRSR2, ZSCAN20, ZXDC, ZZZ3, ВAР1, КМТ2С, ТР53 |
| 1021 | 25 | AASDH, AATF, ABCA9, ABCB5, ABCC1, ABCD4, ABI3BP, ABL1, ACACB, ACAN, ACSBG1, ACTG2, ADAM2, ADAM22, ADAM23, ADAM30, ADAMDEC1, ADAMTSL3, ADCY7, ADIPOR1, ADPRHL1, AEBP2, AGO2, AGPS, AHI1, AIM2, AKT1, AKT2, AKT3, ALDH1B1, ALG6, ALG9, ALK, AMER1, ANK2, ANKFY1, ANKHD1, ANKRD11, ANKRD30B, ANKS1B, ANXA1, ANXA7, AOAH, AP3B1, APC, APCDD1, APOB, AR, ARAF, ARAP3, ARFRP1, ARHGAP11A, ARHGAP35, ARHGEF25, ARID1A, ARID1B, ARID2, ARID5B, ARRDC3, ASB2, ASB3, ASTN2, ASXL1, ASXL2, ATAD5, ATF5, ATF7IP, ATG13, ATM, ATP2A2, ATP2B3, ATPAF1, ATR, ATRX, AURKA, AURKB, AXIN1, AXIN2, AXL, B4GALNT2, BAAT, BAP1, BBC3, BCCIP, BCL11A, BCL11B, BCL2, BCL2L11, BCL2L2, BCL6, BCOR, BCORL1, BCR, BECN1, BEND4, BIRC3, BLM, BMPR1A, BMPR2, BMS1, BRAF, BRCA1, BRCA2, BRD4, BRINP3, BRIP1, BRP44L, BRWD1, BTG1, BTK, BUB1, C10ORF140, C10ORF90, C11ORF41, C12ORF26, C12ORF51, C15ORF2, C15ORF44, C21ORF33, C2ORF71, C2ORF77, C3ORF19, C4ORF17, C6ORF168, C9ORF117, C9ORF79, C9ORF86, CABYR, CACNA1G, CACNA1I, CADM2, CADM3, CALCA, CALR, CAPZA3, CARD11, CARM1, CASP12, CASP8, CBFA2T2, CBL, CBLB, CCDC113, CCDC15, CCDC164, CCDC83, CCND1, CCND2, CCND3, CCNE1, CCNT2, CD19, CD1B, CD274, CD2AP, CD5L, CD74, CD74-NRG1, CD79B, CD9, CDC73, CDH18, CDH7, CDK12, CDK4, CDK6, CDK8, CDKN1A, CDKN1C, CDKN2A, CEBPA, CENPA, CENPF, CEP192, CEP350, CHD1, CHD2, CHD4, CHEK1, CHEK2, CHRM2, CHRNB3, CHST12, CHSY1, CIC, CLCN1, CLDND1, CLIP3, CNDP1, CNNM1, CNOT2, CNST, CNTN5, CNTN6, CNTNAP2, CNTNAP4, CNTNAP5, COBL, COG8, COL14A1, COL16A1, COL17A1, COL22A1, COL2A1, COL3A1, COL4A3, COL9A1, CORIN, CPEB4, CPS1, CPT1A, CPXM2, CREBBP, CRKL, CROCC, CSDE1, CSF1R, CSF3R, CSMD1, CSMD2, CSMD3, CTAGE10P, CTCF, CTDP1, CTLA4, CTNNB1, CTNND2, CUL3, CUL4A, CUL4B, CXCR4, CXORF22, CXORF26, CYBB, CYLD, CYP17A1, CYP4F2, CYSLTR2, DAG1, DAGLA, DAK, DAXX, DCAF8L2, DCHS2, DCST2, DCUN1D1, DDR2, DDX10, DDX27, DDX3X, DDX51, DEFB112, DENND1A, DENND3, DHODH, DICER1, DIDO1, DIS3, DMXL2, DNAH11, DNAH2, DNAH6, DNAH9, DNAJB1, DNAJB8, DNMT1, DNMT3A, DNMT3B, DOT1L, DPP10, DPP6, DPYD, DPYS, DROSHA, DSCAM, DSG1, DSG3, DTX1, DYNC1H1, DYNC2H1, DYNC2LI1, DZIP3, E2F3, EAPP, EDC4, EDN3, EFCAB5, EFR3A, EGER, EGFL7, EGFR, EHD1, EIF2B1, ELF3, ELMO1, ELMO2, EML4, EML5, EMR2, EMSY, EP300, EPAS1, EPB41L4B, EPC1, EPHA2, EPHA3, EPHA5, EPHA7, EPHB1, EPPK1, ERBB2, ERBB3, ERBB4, ERC2, ERCC1, ERCC2, ERCC3, ERCC5, ERCC6, ERG, ERRFI1, ESR1, ESR2, ETV1, ETV4, ETV6, EWSR1, EXT1, EXT2, EXTL3, EYA2, EYS, F11R-NRG1, FABP1, FAM114A2, FAM124B, FAM129B, FAM13C, FAM160B2, FAM193A, FAM47B, FAM58A, FAM65B, FAM71A, FAM76A, FANCA, FANCB, FANCC, FANCD2, FANCE, FANCG, FANCI, FANCM, FAR2, FAT1, FAT3, FAT4, FBXO34, FBXO38, FBXO39, FBXW7, FCGR2B, FGA, FGF12, FGF14, FGF19, FGF23, FGF3, FGF4, FGF6, FGFR1, FGFR2, FGFR3, FGFR4, FH, FHAD1, FIGN, FLCN, FLT1, FLT3, FLT4, FN1, FNIP1, FOXA2, FOXM1, FOXP1, FOXP2, FOXR2, FREM3, FRS2, FSTL5, FTL, FTSJD1, FUBP1, FYCO1, FYN, GABRB2, GABRB3, GABRG2, GABRG3, GALNT12, GALR3, GATA1, GATA2, GATA3, GATA4, GATA6, GBP7, GCC2, GDF11, GDF2, GGH, GKAP1, GLI1, GLIPR2, GML, GNAS, GNONES, GNPTAB, GOLGA3, GP5, GPC3, GPR75, GPR98, GPRIN2, GPX5, GRIA4, GRID1, GRIN2A, GSK3B, GSX1, GTF2B, GTF3C1, H3F3A, H3F3B, H3F3C, HAUS1, HDAC2, HDAC9, HEATR3, HEATR7B2, HEATR8, HELZ, HER2, HGF, HIF1A, HIF1AN, HIST1H1C, HIST1H1E, HIST1H2BD, HIST1H3B, HIST1H3D, HIST1H3G, HIST1H3I, HIST3H3, HIVEP1, HLA-A, HLA-B, HLTF, HMCN1, HMGA2, HNF1A, HRAS, HRASLS5, HSP90AA1, HTR1A, HTR2A, ICOSLG, ID3, IDH1, IDH2, IFI27L2, IFNGR1, IFNL2, IGF1, IGF1R, IGF2, IGFN1, IGSF5, IKBKE, IKZF1, IL22RA1, IL6R, IL7R, IMMP1L, INADL, INHA, INHBA, INPP4A, INPP4B, INPP5B, INPPL1, INSR, IPO11, IPO7, IRF4, IRS1, IRS2, ITGA10, ITGA11, ITGB4, ITIH2, ITLN2, ITM2B, ITSN2, JAK1, JAK2, JAK3, JAKMIP1, JMJD4, JUN, KANSL2, KAT6A, KAT6B,RP11-77G23.2, KDM4A, KDM5A, KDM5B, KDM5C, KDM6A, KDR, KEAP1, KEL, KIAA0232, KIAA1033, KIAA1107, KIAA1217, KIAA1429, KIAA1549, KIAA1614, KIAA1731, KIAA1967, KIF19, KIF24, KIF4A, KIF5B, KIT, KLF4, KLHL1, KLHL6, KLHL7, KMT2A, KMT2B, KMT2C, KMT2D, KMT2С, KNSTRN, KPRP, KRAS, KRT14, KRT28, KRTAP15-1, KRTAP27-1, KYNU, LACTB, LAMA4, LATS1, LATS2, LBP, LEF1, LENG8, LGI4, LILRB4, LIN54, LOC283116, LOC388499, LOXHD1, LPP, LRFN5, LRG1, LRIG2, LRIT3, LRP1B, LRP2, LRRC43, LRRC4C, LRRC72, LRRK2, LTBP1, LUM, LUZP2, LYN, LZTR1, MAGEA10, MAGED2, MAGI1, MAGI2, MALT1, MAMLD1, MAP1B, MAP23K1, MAP2K1, MAP2K2, MAP2K4, MAP3K1, MAP3K13, MAPKBP1, MARS, MASP1, MAX, MBD5, MCL1, MCM3AP, MCMBP, MDC1.00, MDM2, MDM4, MDN1, MED12, MED14, MED17, MED6, MEF2B, MEFV, MEN1, MET, MGA, MIB1, MICALL1, MIS18A, MITF, MKI67, MKRN3, MLANA, MLF1, MLH1, MLH3, MLL, MLL3, MLLT1, MMRN1, MMS22L, MOCS3, MORC1, MORC3, MORF4L1, MPL, MPP6, MRE11A, MRPL24, MS4A6A, MSH2, MSH3, MSH6, MSI2, MST1R, MTF1, MTFMT, MTOR, MTR, MUC16, MUC17, MYB, MYC, MYCBP2, MYCN, MYEOV2, MYF5, MYH15, MYH16, MYH4, MYO10, MYO18B, MYO1A, MYO1B, MYO3A, MYO5B, MYOD1, NAA50, NAB2, NAE1, NANP, NAV2, NAV3, NBEA, NBN, NCAN, NCKAP1, NCOA1, NCOA3, NCOR1, NEGR1, NEK11, NETO2, NEUROD1, NEUROD6, NF1, NF2, NFE2L2, NFIX, NFKBIA, NINL, NKX2-1, NLGN1, NLRP13, NMD3, NMUR2, NOBOX, NOS1, NOTCH1, NOTCH2, NOTCH3, NOTCH3-CNTNAP4, NOTCH4, NPM1, NPY1R, NR4A3, NRAS, NRG1, NRK, NSD1, NTHL1, NTRK1, NTRK2, NTRK3, NUF2, NUMA1, NUP153, NUP93, OBSCN, ODZ3, OPN4, OR10G7, OR10G8, OR10H4, OR10K2, OR11L1, OR1A1, OR2AK2, OR2L3, OR2L8, OR2M7, OR2W5, OR4N2, OR51E2, OR51L1, OR5AP2, OR5D14, OR5D18, OR5L1, OR5T1, OR5T2, OR8J1, ORC5, OTOG, PAK1, PAK3, PAK7, PALB2, PAPPA, PAPPA2, PARK2, PARP1, PARP2, PAX3, PAX5, PBRM1, PCDH18, PCDHA13, PCDHA7, PCDHB10, PCDHB11, PCDHB13, PCDHB14, PCDHGB3, PCLO, PCNT, PDCD1, PDCD11, PDE6A, PDGFRA, PDGFRB, PDHA2, PDPK1, PDYN, PDZD2, PELI3, PER1, PER3, PET112, PEX19, PGAM5, PGBD2, PGR, PHF15, PHF23, PHF8, PHOX2B, PIBF1, PIEZO2, PIF1, PIGC, PIGR, PIK3C2A, PIK3C2B, PIK3C2G, PIK3C3, PIK3CA, PIK3CD, PIK3CG, PIK3R1, PIK3R2, PIK3СА, PIM1, PKM2, PLA2G15, PLCB4, PLCG2, PLCH1, PLEKHA7, PLS3, PMS1, PMS2, PNCK, POLD1, POLE, POM121L12, POM121L2, POT1, POTEA, POU5F2, PPARG, PPFIA2, PPM1D, PPP2CB, PPP2R1A, PPP2R2A, PPP4R1, PPP4R2, PPP6C, PRDM1, PRDM10, PREX2, PRKAG3, PRKCA, PRKCI, PRKD1, PRKDC, PRKN, PRMT10, PRR25, PRSS1, PRSS35, PRTG, PTBP3, PTCH1, PTEN, PTK2, PTK7, PTN, PTPN11, PTPRB, PTPRD, PTPRN2, PTPRS, PTPRT, PTPRZ1, PWP2, PXDNL, PZP, RAB3GAP2, RABEP2, RAC2, RAD21, RAD50, RAD51C, RAD52, RAD54L, RAF1, RALGAPA2, RANBP2, RAP2A, RASA1, RB1, RBM10, RBM15, RCL1, RCN1, RECQL, RECQL4, REL, RELN, RET, RHBDF2, RICTOR, RIF1, RIMS2, RIT1, RLIM, RNF123, RNF157, RNF43, ROBO2, ROBO3, ROS1, RP1L1, RPL3, RPL5, RPS26, RPS6KA1, RPS6KA4, RPTOR, RRAS, RRAS2, RSF1, RUNX1T1, RUSC2, RXRA, RYR2, S1PR3, SAMD4A, SATL1, SBDS, SCAF11, SCN10A, SCN3A, SCN9A, SDHA, SDHB, SDHC, SDK1, SDR42E1, SEMA3A, SEMA3E, SEMA6A, SERPINA11, SERPINB4, SERPINI2, SESN1, SETBP1, SETD1A, SETD2, SF3B1, SGIP1, SH2B3, SHOC2, SHQ1, SI, SIPA1L2, SLC16A7, SLC22A2, SLC22A24, SLC25A34, SLC27A3, SLC29A1, SLC34A2, SLC38A4, SLC4A7, SLC5A3, SLC6A17, SLC8A3, SLIT2, SLITRK1, SLITRK2, SLITRK3, SLITRK4, SLX4, SMAD2, SMAD3, SMAD4, SMARCA1, SMARCA4, SMARCB1, SMCR7L, SMG7, SMO, SMYD1, SNAP25, SNCAIP, SORCS2, SOS1, SOX17, SOX2, SOX4, SOX6, SOX9, SPAG5, SPATA16, SPATA4, SPATS2, SPEF2, SPEG, SPEN, SPET9, SPG11, SPNS3, SPOP, SPTA1, SPTBN1, SPTLC3, SPZ1, SRC, SRRM2, SRSF2, SRSF5, SS18, SST, STAG2, STAM, STAT3, STAT5B, STK11, STRN, STXBP5L, SUZ12, SVIL, SWT1, SYCP2, SYK, SYNE1, SYNPO2, TAF1, TAF6, TAP1, TBC1D22B, TBC1D23, TBC1D4, TBX3, TCF3, TCF4, TCF7L2, TCHHL1, TCP11L2, TCRBV7S1A1N2T, TECTA, TEK, TERT, TET1, TET2, TGFBR1, TGFBR2, TGM6, TIMP4, TKTL2, TLR4, TM9SF4, TMCO3, TMCO7, TMEM100, TMPRSS11B, TMTC3, TNFAIP3, TNFRSF10D, TNFRSF14, TNIK, TNKS2, TNR, TNRC6A, TNRC6B, TOP1.00, TOP1MT, TP53BP1, TP63, TPMT, TPRA1, TPTE, TRAF7, TRANK1, TRIM29, TRIM58, TRIML1, TRIP12, TROAP, TRPA1, TRPC6, TSC1, TSC2, TSHR, TSHZ2, TSKU, TSPAN31, TSPEAR, TTC23, TTN, TUBAIA, TUBB8, TXLNB, U2AF1, UBP1, UBR2, UGT1A1, UNC79, UNC80, UPB1, UPF1, USP15, USP24, USP34, USP48, USP9X, VAV1, VEGFA, VEZF1, VHL, VIL1, VPRBP, VTCN1, VWA3A, VWF, WAS, WASL, WDFY4, WDR43, WDR49, WDR64, WDR72, WDR81, WDR83, WHSC1, WHSC1L1, WISP3, WNT10B, WRAP53, WRN, WT1, WTAP, XIAP, XIRP2, XKR4, XPO1, XPO6, XRCC1, ZAK, ZBED5, ZBP1, ZBTB16, ZBTB8A, ZDHHC11, ZER1, ZFAT, ZFHX3, ZFHX4, ZFPM2, ZIC4, ZNF14, ZNF160, ZNF212, ZNF257, ZNF367, ZNF426, ZNF462, ZNF534, ZNF555, ZNF681, ZNF683, ZNF703, ZNF814, ZNF831, ZNF99, ZRSR2, ZSCAN20, ZXDC, ZZZ3, ВAР1, КМТ2С, ТР53  (Only partially displayed, not fully shown) |
| 808 | 30 | ABCB1, ABCC9, ABL1, ABL2, ACVR1, ACVR1B, ADAMTS12, ADGRG4, AGO2, AKT1, AKT2, AKT3, ALK, ALOX12B, AMER1, AMOT, ANK2, ANKRD11, APC, APCDD1, APOB, AR, ARAF, ARFRP1, ARID1A, ARID1B, ARID2, ARID5B, ASB18, ASTN1, ASTN2, ASXL1, ASXL2, ASXL3, ATIC, ATM, ATR, ATRX, AURKA, AURKB, AXIN1, AXIN2, AXL, B2M, BABAM1, BACH1, BAP1, BARD1, BBC3, BCL10, BCL2, BCL2L1, BCL2L11, BCL2L2, BCL6, BCOR, BCORL1, BCR, BIRC3, BLM, BMPR1A, BRAF, BRCA1, BRCA2, BRD4, BRINP3, BRIP1, BTG1, BTK, C11ORF30, C14ORF177, C17ORF39, C2ORF44, C6ORF118, C8ORF34, CA10, CALR, CARD11, CARM1, CARS, CASP7, CASP8, CBFB, CBL, CCDC6, CCND1, CCND2, CCND3, CCNE1, CD274, CD276, CD74, CD79A, CD79B, CDA, CDC42, CDC73, CDH1, CDH10, CDH12, CDH18, CDH9, CDK12, CDK4, CDK5RAP2, CDK6, CDK8, CDKN1A, CDKN1B, CDKN2A, CDKN2B, CDKN2C, CEBPA, CENPA, CFTR, CHD2, CHD4, CHEK1, CHEK2, CHST3, CHUK, CIC, CLIP1, CLTC, CNTNAP2, CNTNAP5, COL22A1, CRBN, CREBBP, CRKL, CRLF2, CSDE1, CSF1R, CSF3R, CSMD3, CTCF, CTLA4, CTNNA1, CTNNA2, CTNNB1, CUL3, CUL4A, CUL4B, CXCR4, CYLD, CYP17A1, CYP19A1, CYP1A1, CYP1B1, CYP2C8, CYP2D6, CYP2E1, CYP3A4, CYP3A5, CYP4B1, CYSLTR2, DAXX, DCAF12L1, DCAF12L2, DCAF4L2, DCK, DCTN1, DCUN1D1, DDR2, DICER1, DIS3, DMD, DNAJB1, DNMT1, DNMT3A, DNMT3B, DOT1L, DPYD, DROSHA, DUSP27, DUSP4, DYNC2H1, E2F3, EED, EGF, EGFL7, EGFR, EIF1AX, EIF4A2, EIF4E, ELF3, EML4, EMSY, ENG, EP300, EPAS1, EPCAM, EPHA2, EPHA3, EPHA5, EPHA7, EPHB1, ERBB2, ERBB3, ERBB4, ERC1, ERCC1, ERCC2, ERCC3, ERCC4, ERCC5, ERF, ERG, ERICH3, ERRFI1, ESR1, ETV1, ETV4, ETV5, ETV6, EWSR1, EZH1, EZH2, EZR, F3, FAM123B, FAM135B, FAM175A, FAM46C, FAM58A, FANCA, FANCB, FANCC, FANCD1, FANCD2, FANCE, FANCF, FANCG, FANCI, FANCL, FANCM, FAS, FAT1, FAT3, FBN2, FBXL7, FBXW7, FDPS, FES, FGD1, FGF10, FGF12, FGF14, FGF19, FGF23, FGF3, FGF4, FGF6, FGF7, FGFR1, FGFR1OP, FGFR2, FGFR3, FGFR4, FH, FIP1L1, FLCN, FLT1, FLT3, FLT4, FN1, FOXA1, FOXL2, FOXO1, FOXP1, FRK, FRS2, FRYL, FUBP1, FYN, GABRA2, GABRA6, GALNT12, GALNT14, GATA1, GATA2, GATA3, GATA4, GATA6, GEN1, GID4, GLI1, GNA11, GNA13, GNAQ, GNAS, GOLGA5, GOPC, GPR124, GPR158, GPS2, GREM1, GRID1, GRIK3, GRIN2A, GRM3, GRM8, GSK3B, GSTA1, GSTP1, H3F3A, H3F3AP4, H3F3B, H3F3C, HAPLN1, HCN1, HDAC9, HFE, HGF, HIF2A, HIP1, HIST1H1C, HIST1H2BD, HIST1H3A, HIST1H3B, HIST1H3C, HIST1H3D, HIST1H3E, HIST1H3F, HIST1H3G, HIST1H3H, HIST1H3I, HIST1H3J, HIST2H3A, HIST2H3C, HIST2H3D, HIST3H3, HLA-A, HLA-B, HNF1A, HOOK3, HOXB13, HRAS, HSD3B1, HSP90AA1, HTR1A, ICOSLG, ID3, IDH1, IDH2, IFNGR1, IFNL3, IFNL4, IGF1, IGF1R, IGF2, IGFL3, IKBKE, IKZF1, IL10, IL7R, IMPDH2, INHA, INHBA, INPP4A, INPP4B, INPPL1, INSR, IQCJ, IRF2, IRF4, IRS1, IRS2, JAK1, JAK2, JAK3, JUN, KAT6A, KCNA4, KCND2, KCNJ3, KCNT2, KDM5A, KDM5C, KDM6A, KDR, KEAP1, KEL, KIAA1598, KIF2B, KIF5B, KIT, KLC1, KLF4, KLHL1, KLHL6, KMT2A, KMT2B, KMT2C, KMT2D, KMT5A, KNSTRN, KRAS, KTN1, LATS1, LATS2, LMO1, LOC349160, LPL, LPPR4, LRFN5, LRIG3, LRP1B, LRRC4C, LRRIQ3, LRRK2, LRRTM4, LTK, LYN, LZTR1, MAGI2, MALT1, MAP2K1, MAP2K2, MAP2K4, MAP3K1, MAP3K13, MAP3K14, MAP3K5, MAPK1, MAPK3, MAPKAP1, MARCH1, MAX, MCL1, MDC1, MDH2, MDM2, MDM4, MED12, MEF2B, MEF2BNB-MEF2B, MEN1, MET, MGA, MIR4728, MITF, MKRN3, MLH1, MLL3, MPL, MRE11A, MS4A3, MSH2, MSH3, MSH6, MSI1, MSI2, MSN, MST1, MST1R, MTHFR, MTOR, MTR, MTRR, MUTYH, MYB, MYC, MYCL, MYCL1, MYCN, MYD88, MYO5A, MYOD1, MYST3, NAT2, NAV3, NBN, NCAM1, NCOA3, NCOA4, NCOR1, NEGR1, NF1, NF2, NFE2L2, NFKBIA, NKX2-1, NKX3-1, NLRP3, NLRP5, NOS3, NOTCH1, NOTCH2, NOTCH3, NOTCH4, NPM1, NQO1, NRAS, NSD1, NTHL1, NTM, NTRK1, NTRK2, NTRK3, NUDT15, NUF2, NUP93, NUTM1, PAK1, PAK3, PAK5, PAK6, PAK7, PALB2, PALLD, PAPPA2, PARK2, PARP1, PARP2, PARP3, PARP4, PAX5, PAX8, PBRM1, PCDH10, PCDH17, PCM1, PDCD1, PDCD1LG2, PDGFRA, PDGFRB, PDHA2, PDK1, PDPK1, PDYN, PDZRN3, PGR, PHOX2B, PIK3C2B, PIK3C2G, PIK3C3, PIK3CA, PIK3CB, PIK3CD, PIK3CG, PIK3R1, PIK3R2, PIK3R3, PIM1, PLCG2, PLK2, PMAIP1, PML, PMS1, PMS2, PNRC1, POLD1, POLDIP2, POLE, POM121L12, PPARD, PPARG, PPFIBP1, PPM1D, PPP2R1A, PPP4R2, PPP6C, PRDM1, PRDM14, PREX2, PRIM2, PRKACA, PRKAR1A, PRKCI, PRKD1, PRKDC, PRSS1, PRSS8, PTCH1, PTEN, PTP4A1, PTPN11, PTPRD, PTPRS, PTPRT, PWWP2A, PXDNL, QKI, RAB35, RAC1, RAC2, RAD21, RAD50, RAD51, RAD51B, RAD51C, RAD51D, RAD52, RAD54L, RAF1, RANBP2, RARA, RASA1, RB1, RBM10, RECQL, RECQL3, RECQL4, REG3A, REL, REQ4, RET, RFWD2, RHBDF2, RHEB, RHOA, RICTOR, RINT1, RIT1, RNF43, ROCK1, ROS1, RP1L1, RPA1, RPL11, RPL13, RPL35A, RPL5, RPS10, RPS17, RPS19, RPS24, RPS26, RPS6KA4, RPS6KB2, RPS7, RPTOR, RRAGC, RRAS, RRAS2, RRM1, RRM2, RRM2B, RTEL1, RUNX1, RUNX1T1, RXRA, RYBP, RYR2, SALL1, SDC4, SDHA, SDHAF2, SDHB, SDHC, SDHD, SEC31A, SEMA3C, SESN1, SESN2, SESN3, SETBP1, SETD2, SF3B1, SH2B3, SH2D1A, SHOC2, SHQ1, SLC14A2, SLC19A1, SLC22A12, SLC34A2, SLC45A2, SLC8A1, SLCO1B1, SLCO1B3, SLIT2, SLIT3, SLITRK1, SLITRK2, SLITRK3, SLX4, SMAD2, SMAD3, SMAD4, SMARCA4, SMARCB1, SMARCD1, SMO, SMYD3, SNCAIP, SOCS1, SOD2, SOS1, SOX10, SOX17, SOX2, SOX9, SPEN, SPG7, SPHKAP, SPINK1, SPOP, SPRED1, SPTA1, SQSTM1, SRC, SRSF2, ST6GAL2, STAG2, STAT3, STAT4, STAT5A, STAT5B, STK11, STK19, STK40, STRN, SUFU, SULT2B1, SUZ12, SYK, TAF1, TAP1, TAP2, TBX3, TCEB1, TCF3, TCF7L2, TEK, TEKT4, TERC, TERT, TET1, TET2, TFG, TG, TGFBR1, TGFBR2, TGFBR3, TIPARP, TLR4, TMEM127, TMPRSS2, TNFAIP3, TNFRSF14, TNN, TNR, TOP1, TOP2A, TP53, TP53BP1, TP63, TPM3, TPM4, TPMT, TRAF2, TRAF7, TRIM24, TRIM27, TRIM33, TRIM58, TRPC5, TRRAP, TSC1, TSC2, TSHR, TSHZ3, TUBB1, TYK2, TYMS, U2AF1, UGT1A1, UGT1A8, UMPS, UPF1, VCL, VEGFA, VHL, VTCN1, WHSC1, WHSC1L1, WISP3, WRN, WT1, WWTR1, XIAP, XIRP2, XPC, XPO1, XRCC1, XRCC2, XRCC3, YAP1, YES1, ZAN, ZBTB2, ZCCHC8, ZFHX3, ZFHX4, ZIC1, ZIC4, ZIM2, ZNF217, ZNF423, ZNF521, ZNF536, ZNF703, ZNF804A, ZNF804B, ZNF831, ZRSR2 |
| 605 | 51 | ABL1, AKT1, AKT2, AKT3, ALK, APC, AR, ARAF, ARID1A, ARID1B, ARID2, ASXL1, ATM, ATR, ATRX, AURKA, AURKB, AXIN1, AXIN2, AXL, BAP1, BCL2, BCL2L11, BCL6, BCOR, BLM, BMPR1A, BRAF, BRCA1, BRCA2, BRD4, BRIP1, BTK, CARD11, CASP8, CBL, CCND1, CCND2, CCND3, CCNE1, CD274, CD79B, CDC73, CDK12, CDK4, CDK6, CDK8, CDKN1A, CDKN2A, CHEK1, CHEK2, CIC, CREBBP, CRKL, CSF1R, CTCF, CTNNB1, CUL3, DAXX, DDR2, DICER1, DNMT3A, DOT1L, EGFR, EP300, EPHA3, EPHA5, EPHB1, ERBB2, ERBB3, ERBB4, ERCC2, ERCC3, ERCC5, ERG, ESR1, ETV1, ETV6, FANCA, FANCC, FAT1, FBXW7, FGF19, FGF3, FGF4, FGFR1, FGFR2, FGFR3, FGFR4, FH, FLCN, FLT1, FLT3, FLT4, FOXP1, FUBP1, GATA1, GATA2, GATA3, GNAS, GRIN2A, GSK3B, HGF, HNF1A, HRAS, IDH1, IDH2, IGF1R, IGF2, IKBKE, IKZF1, IL7R, INPP4B, IRF4, IRS2, JAK1, JAK2, JAK3, JUN, KDM5A, KDM5C, KDM6A, KDR, KEAP1, KIT, KLF4, KRAS, LATS1, LATS2, MAP2K1, MAP2K2, MAP2K4, MAP3K1, MAX, MCL1, MDM2, MDM4, MED12, MEF2B, MEN1, MET, MITF, MLH1, MPL, MRE11A, MSH2, MSH6, MTOR, MYC, MYCN, MYOD1, NBN, NF1, NF2, NFE2L2, NKX2-1, NOTCH1, NOTCH2, NOTCH3, NPM1, NRAS, NSD1, NTRK1, NTRK2, NTRK3, PAK1, PALB2, PARK2, PARP1, PAX5, PBRM1, PDCD1, PDGFRA, PDGFRB, PDPK1, PIK3CA, PIK3CD, PIK3CG, PIK3R1, PIK3R2, PIM1, PMS1, PMS2, POLE, PPP2R1A, PRDM1, PTCH1, PTEN, PTPN11, PTPRD, PTPRT, RAD50, RAD51C, RAD52, RAD54L, RAF1, RB1, RBM10, RECQL4, REL, RET, RICTOR, RIT1, RNF43, ROS1, RPTOR, SDHA, SDHB, SDHC, SETD2, SF3B1, SMAD2, SMAD3, SMAD4, SMARCA4, SMARCB1, SMO, SOX2, SOX9, SPEN, SPOP, SRC, STAG2, STK11, SUZ12, SYK, TBX3, TERT, TET1, TET2, TGFBR2, TNFAIP3, TNFRSF14, TSC1, TSC2, TSHR, U2AF1, VHL, WT1, XPO1, AMER1, B2M, BARD1, BCL2L1, BCORL1, CALR, CBFB, CDH1, CDKN1B, CDKN2B, CDKN2C, CEBPA, CHD4, CNTNAP5, CRLF2, CSF3R, CSMD3, CXCR4, CYLD, EED, EPCAM, EPHA2, EPHA7, ERCC1, ERCC4, ERRFI1, ETV4, EWSR1, EZH2, FAM175A, FAM46C, FANCB, FANCG, FANCI, FGF23, FGF6, FOXA1, FOXL2, GATA6, GLI1, GNA11, GNAQ, GREM1, H3F3A, HSP90AA1, INHBA, KMT2A, KMT2B, KMT2C, KMT2D, LMO1, LRP1B, LYN, MAPK1, MDC 1.00, MSH3, MST1R, MUTYH, MYD88, NCOA3, NFKBIA, NUP93, PGR, PIK3CB, PLCG2, POLD1, PREX2, PRKAR1A, PRKCI, PRSS1, RAC1, RAD21, RAD51, RARA, RECQL, RHBDF2, RHOA, RUNX1, SDHAF2, SDHD, SETBP1, SH2B3, SLX4, SOCS1, SRSF2, STAT3, SUFU, TMEM127, TMPRSS2, TOP 1.00, TP53, TPMT, UGT1A1, VEGFA, WRN, YAP1, YES1, ACVR1, ARID5B, ASXL2, BBC3, BUB1, CADM2, CBLB, CD79A, CDKN1C, CTLA4, CYP19A1, DCUN1D1, DDX3X, DDX51, DIS3, DNMT1, DNMT3B, DPYD, DYNC2H1, E2F3, EGFL7, ESR2, EXT1, EXT2, FANCL, FGF10, FGF7, FOXM1, FOXP2, GEN1, GPRIN2, H3F3C, HDAC2, HIF1A, HIST1H1C, HIST1H2BD, HIST1H3B, HMGA2, HOXB13, ICOSLG, IFNGR1, IFNL2, IGF1, INPP4A, INSR, IRS1, KRT14, LRIG3, MAP3K13, MAPK3, MAPKBP1, MKI67, MLH3, MYCL, NAB2, NCOR1, NOTCH4, NR4A3, NRG1, NUTM1, PAK7, PALLD, PAX3, PAX8, PDCD1LG2, PHOX2B, PIK3C2G, PIK3C3, PPP2R2A, PRKACA, PTN, PTPRS, PZP, RAD51B, RAD51D, RASA1, RHEB, RIF1, ROBO2, RPS6KA4, RRAS2, RSF1, SBDS, SHQ1, SOX17, SPINK1, SS18, SYNE1, TGFBR1, TP63, TRAF7, TSPAN31, TYMS, VTCN1, WAS, XIAP, XRCC1, ZBTB16, ZNF367, ABCB1, ABCC1, ACTL6A, ADH1B, AGO2, ALDH2, ALOX12B, ANK2, ANKRD11, ANXA5, APCDD1, APOB, AREG, ARFRP1, ASNS, ASPH, ASTN2, BCL2L2, BCR, BIRC3, BRD2, BRINP3, BTG1, C8ORF34, CACNA1C, CAMTA1, CAPN2, CARM1, CASP7, CCL18, CD276, CD74, CDA, CDH18, CENPA, CFD, CHD2, CNTNAP2, COL22A1, CSDE1, CUL4A, CUL4B, CYP17A1, CYP1A1, CYP1B1, CYP2C8, CYP2D6, CYP 20.00, CYP3A4, CYP3A5, CYSLTR2, DDIT3, DNAJB1, DROSHA, DSCAM, EGF, EIF1AX, ELAC2, ELF3, EML4, EMSY, EPAS1, EREG, FAM123B, FAM58A, FANCD2, FANCE, FANCM, FAT3, FBN3, FGF1, FGF12, FGF14, FGF2, FGF5, FGF8, FGF9, FN1, FOXK2, FRS2, FUS, FYN, GAB2, GALNT12, GALNT14, GATA4, GEMIN6, GGH, GK5, GLIPR1, GLRX, GMEB1, GPER1, GPX5, GRID1, GSTA1, GSTP1, H3F3B, HDAC9, HFE2, HIST1H3D, HIST1H3G, HIST1H3I, HIST3H3, HLA-A, HLA-B, HNF1B, HSD17B3, HSD3B2, HTR1A, ID3, IFNLR1, IL10, INHA, INPPL1, KAT6A, KCNJ5, KEL, KIF1B, KIF5B, KLHL1, KLHL6, KLLN, KNSTRN, KRT15, KRT5, LARP4, LBR, LRFN5, LRP2, LRRC4C, LRRK2, LZTR1, MAGI2, MALT1, MGA, MKRN3, MLL3, MSI2, MTHFR, MTR, MTUS1, MYB, MYCL1, NAT2, NAV3, NCOA1, NEGR1, NKX3-1, NOS3, NOVA1, NQO1, NTHL1, NUF2, PAK3, PAPPA2, PARP2, PAX7, PCBP1, PDGFB, PDHA2, PDYN, PIK3C2B, PIK3R3, PLAUR, PLIN2, PLK2, PMAIP1, PNRC1, POM121L12, PPARG, PPIB, PPM1D, PPP4R2, PPP6C, PRKACB, PRKD1, PRKDC, PSME2, PXDNL, RAC2, RANBP2, RECK, RFWD2, RILP, RNASEL, RP1L1, RPL5, RPS26, RPS6KB1, RPS6KB2, RRAS, RRM1, RUNX1T1, RXRA, RYBP, RYR2, SCN10A, SELL, SEMA3C, SESN1, SETD7, SH2D1A, SHOC2, SHOX, SLC19A1, SLC22A2, SLC29A1, SLC34A2, SLCO1B1, SLCO1B3, SLIT2, SLITRK1, SLITRK2, SLITRK3, SMARCD1, SNCAIP, SOCS6, SOD2, SOS 1, SOX10, SPTA1, SRD5A2, STAT5B, STK40, STRN, SULT1A1, TAF1, TAP1, TCF3, TCF7L2, TEK, TERC, TFE3, TLR4, TNFRSF19, TNFSF8, TNR, TP53BP1, TRAF1, TRIM58, TUBB1, UPF1, WHSC1, WHSC1L1, WISP3, XIRP2, XPC, XRCC3, ZFHX3, ZFHX4, ZIC4, ZNF423, ZNF703, ZNF717, ZNF750, ZNF831, ZRSR2 |
| 520 | 16 | ABL1, AKT1, AKT2, AKT3, ALK, APC, AR, ARAF, ARID1A, ARID1B, ARID2, ASXL1, ATM, ATR, ATRX, AURKA, AURKB, AXIN1, AXIN2, AXL, BAP1, BCL2, BCL2L11, BCL6, BCOR, BLM, BMPR1A, BRAF, BRCA1, BRCA2, BRD4, BRIP1, BTK, CARD11, CASP8, CBL, CCND1, CCND2, CCND3, CCNE1, CD274, CD79B, CDC73, CDK12, CDK4, CDK6, CDK8, CDKN1A, CDKN2A, CHEK1, CHEK2, CIC, CREBBP, CRKL, CSF1R, CTCF, CTNNB1, CUL3, DAXX, DDR2, DICER1, DNMT3A, DOT1L, EGFR, EP300, EPHA3, EPHA5, EPHB1, ERBB2, ERBB3, ERBB4, ERCC2, ERCC3, ERCC5, ERG, ESR1, ETV1, ETV6, FANCA, FANCC, FAT1, FBXW7, FGF19, FGF3, FGF4, FGFR1, FGFR2, FGFR3, FGFR4, FH, FLCN, FLT1, FLT3, FLT4, FOXP1, FUBP1, GATA1, GATA2, GATA3, GNAS, GRIN2A, GSK3B, HGF, HNF1A, HRAS, IDH1, IDH2, IGF1R, IGF2, IKBKE, IKZF1, IL7R, INPP4B, IRF4, IRS2, JAK1, JAK2, JAK3, JUN, KDM5A, KDM5C, KDM6A, KDR, KEAP1, KIT, KLF4, KRAS, LATS1, LATS2, MAP2K1, MAP2K2, MAP2K4, MAP3K1, MAX, MCL1, MDM2, MDM4, MED12, MEF2B, MEN1, MET, MITF, MLH1, MPL, MRE11A, MSH2, MSH6, MTOR, MYC, MYCN, MYOD1, NBN, NF1, NF2, NFE2L2, NKX2-1, NOTCH1, NOTCH2, NOTCH3, NPM1, NRAS, NSD1, NTRK1, NTRK2, NTRK3, PAK1, PALB2, PARK2, PARP1, PAX5, PBRM1, PDCD1, PDGFRA, PDGFRB, PDPK1, PIK3CA, PIK3CD, PIK3CG, PIK3R1, PIK3R2, PIM1, PMS1, PMS2, POLE, PPP2R1A, PRDM1, PTCH1, PTEN, PTPN11, PTPRD, PTPRT, RAD50, RAD51C, RAD52, RAD54L, RAF1, RB1, RBM10, RECQL4, REL, RET, RICTOR, RIT1, RNF43, ROS1, RPTOR, SDHA, SDHB, SDHC, SETD2, SF3B1, SMAD2, SMAD3, SMAD4, SMARCA4, SMARCB1, SMO, SOX2, SOX9, SPEN, SPOP, SRC, STAG2, STK11, SUZ12, SYK, TBX3, TERT, TET1, TET2, TGFBR2, TNFAIP3, TNFRSF14, TSC1, TSC2, TSHR, U2AF1, VHL, WT1, XPO1, AMER1, B2M, BARD1, BCL2L1, BCORL1, CALR, CBFB, CDH1, CDKN1B, CDKN2B, CDKN2C, CEBPA, CHD4, CNTNAP5, CRLF2, CSF3R, CSMD3, CXCR4, CYLD, EED, EPCAM, EPHA2, EPHA7, ERCC1, ERCC4, ERRFI1, ETV4, EWSR1, EZH2, FAM175A, FAM46C, FANCB, FANCG, FANCI, FGF23, FGF6, FOXA1, FOXL2, GATA6, GLI1, GNA11, GNAQ, GREM1, H3F3A, HSP90AA1, INHBA, KMT2A, KMT2B, KMT2C, KMT2D, LMO1, LRP1B, LYN, MAPK1, MDC 1.00, MSH3, MST1R, MUTYH, MYD88, NCOA3, NFKBIA, NUP93, PGR, PIK3CB, PLCG2, POLD1, PREX2, PRKAR1A, PRKCI, PRSS1, RAC1, RAD21, RAD51, RARA, RECQL, RHBDF2, RHOA, RUNX1, SDHAF2, SDHD, SETBP1, SH2B3, SLX4, SOCS1, SRSF2, STAT3, SUFU, TMEM127, TMPRSS2, TOP 1.00, TP53, TPMT, UGT1A1, VEGFA, WRN, YAP1, YES1, ACVR1, ARID5B, ASXL2, BBC3, BUB1, CADM2, CBLB, CD79A, CDKN1C, CTLA4, CYP19A1, DCUN1D1, DDX3X, DDX51, DIS3, DNMT1, DNMT3B, DPYD, DYNC2H1, E2F3, EGFL7, ESR2, EXT1, EXT2, FANCL, FGF10, FGF7, FOXM1, FOXP2, GEN1, GPRIN2, H3F3C, HDAC2, HIF1A, HIST1H1C, HIST1H2BD, HIST1H3B, HMGA2, HOXB13, ICOSLG, IFNGR1, IFNL2, IGF1, INPP4A, INSR, IRS1, KRT14, LRIG3, MAP3K13, MAPK3, MAPKBP1, MKI67, MLH3, MYCL, NAB2, NCOR1, NOTCH4, NR4A3, NRG1, NUTM1, PAK7, PALLD, PAX3, PAX8, PDCD1LG2, PHOX2B, PIK3C2G, PIK3C3, PPP2R2A, PRKACA, PTN, PTPRS, PZP, RAD51B, RAD51D, RASA1, RHEB, RIF1, ROBO2, RPS6KA4, RRAS2, RSF1, SBDS, SHQ1, SOX17, SPINK1, SS18, SYNE1, TGFBR1, TP63, TRAF7, TSPAN31, TYMS, VTCN1, WAS, XIAP, XRCC1, ZBTB16, ZNF367, ABCB1, ABCC1, ACTL6A, ADH1B, AGO2, ALDH2, ALOX12B, ANK2, ANKRD11, ANXA5, APCDD1, APOB, AREG, ARFRP1, ASNS, ASPH, ASTN2, BCL2L2, BCR, BIRC3, BRD2, BRINP3, BTG1, C8ORF34, CACNA1C, CAMTA1, CAPN2, CARM1, CASP7, CCL18, CD276, CD74, CDA, CDH18, CENPA, CFD, CHD2, CNTNAP2, COL22A1, CSDE1, CUL4A, CUL4B, CYP17A1, CYP1A1, CYP1B1, CYP2C8, CYP2D6, CYP 20.00, CYP3A4, CYP3A5, CYSLTR2, DDIT3, DNAJB1, DROSHA, DSCAM, EGF, EIF1AX, ELAC2, ELF3, EML4, EMSY, EPAS1, EREG, FAM123B, FAM58A, FANCD2, FANCE, FANCM, FAT3, FBN3, FGF1, FGF12, FGF14, FGF2, FGF5, FGF8, FGF9, FN1, FOXK2, FRS2, FUS, FYN, GAB2, GALNT12, GALNT14, GATA4, GEMIN6, GGH, GK5, GLIPR1, GLRX, GMEB1, GPER1, GPX5, GRID1, GSTA1, GSTP1, H3F3B, HDAC9, HFE2, HIST1H3D, HIST1H3G, HIST1H3I, HIST3H3, HLA-A, HLA-B, HNF1B, HSD17B3, HSD3B2, HTR1A, ID3, IFNLR1, IL10, INHA, INPPL1, KAT6A, KCNJ5, KEL, KIF1B, KIF5B, KLHL1, KLHL6, KLLN, KNSTRN, KRT15, KRT5, LARP4, LBR, LRFN5, LRP2, LRRC4C, LRRK2, LZTR1, MAGI2, MALT1, MGA, MKRN3, MLL3, MSI2, MTHFR, MTR, MTUS1, MYB, MYCL1, NAT2, NAV3, NCOA1, NEGR1, NKX3-1, NOS3, NOVA1, NQO1, NTHL1, NUF2, PAK3, PAPPA2, PARP2, PAX7, PCBP1, PDGFB, PDHA2, PDYN, PIK3C2B, PIK3R3, PLAUR, PLIN2, PLK2, PMAIP1, PNRC1, POM121L12, PPARG, PPIB, PPM1D, PPP4R2, PPP6C, PRKACB, PRKD1, PRKDC, PSME2, PXDNL, RAC2, RANBP2, RECK, RFWD2, RILP, RNASEL, RP1L1, RPL5, RPS26, RPS6KB1, RPS6KB2, RRAS, RRM1, RUNX1T1, RXRA, RYBP, RYR2, SCN10A, SELL, SEMA3C, SESN1, SETD7, SH2D1A, SHOC2, SHOX, SLC19A1, SLC22A2, SLC29A1, SLC34A2, SLCO1B1, SLCO1B3, SLIT2, SLITRK1, SLITRK2, SLITRK3, SMARCD1, SNCAIP, SOCS6, SOD2, SOS 1, SOX10, SPTA1, SRD5A2, STAT5B, STK40, STRN, SULT1A1, TAF1, TAP1, TCF3, TCF7L2, TEK, TERC, TFE3, TLR4, TNFRSF19, TNFSF8, TNR, TP53BP1, TRAF1, TRIM58, TUBB1, UPF1, WHSC1, WHSC1L1, WISP3, XIRP2, XPC, XRCC3, ZFHX3, ZFHX4, ZIC4, ZNF423, ZNF703, ZNF717, ZNF750, ZNF831, ZRSR2 |
| 500 | 41 | ABL1, AKT1, AKT2, AKT3, ALK, APC, AR, ARAF, ARID1A, ARID1B, ARID2, ASXL1, ATM, ATR, ATRX, AURKA, AURKB, AXIN1, AXIN2, AXL, BAP1, BCL2, BCL2L11, BCL6, BCOR, BLM, BMPR1A, BRAF, BRCA1, BRCA2, BRD4, BRIP1, BTK, CARD11, CASP8, CBL, CCND1, CCND2, CCND3, CCNE1, CD274, CD79B, CDC73, CDK12, CDK4, CDK6, CDK8, CDKN1A, CDKN2A, CHEK1, CHEK2, CIC, CREBBP, CRKL, CSF1R, CTCF, CTNNB1, CUL3, DAXX, DDR2, DICER1, DNMT3A, DOT1L, EGFR, EP300, EPHA3, EPHA5, EPHB1, ERBB2, ERBB3, ERBB4, ERCC2, ERCC3, ERCC5, ERG, ESR1, ETV1, ETV6, FANCA, FANCC, FAT1, FBXW7, FGF19, FGF3, FGF4, FGFR1, FGFR2, FGFR3, FGFR4, FH, FLCN, FLT1, FLT3, FLT4, FOXP1, FUBP1, GATA1, GATA2, GATA3, GNAS, GRIN2A, GSK3B, HGF, HNF1A, HRAS, IDH1, IDH2, IGF1R, IGF2, IKBKE, IKZF1, IL7R, INPP4B, IRF4, IRS2, JAK1, JAK2, JAK3, JUN, KDM5A, KDM5C, KDM6A, KDR, KEAP1, KIT, KLF4, KRAS, LATS1, LATS2, MAP2K1, MAP2K2, MAP2K4, MAP3K1, MAX, MCL1, MDM2, MDM4, MED12, MEF2B, MEN1, MET, MITF, MLH1, MPL, MRE11A, MSH2, MSH6, MTOR, MYC, MYCN, MYOD1, NBN, NF1, NF2, NFE2L2, NKX2-1, NOTCH1, NOTCH2, NOTCH3, NPM1, NRAS, NSD1, NTRK1, NTRK2, NTRK3, PAK1, PALB2, PARK2, PARP1, PAX5, PBRM1, PDCD1, PDGFRA, PDGFRB, PDPK1, PIK3CA, PIK3CD, PIK3CG, PIK3R1, PIK3R2, PIM1, PMS1, PMS2, POLE, PPP2R1A, PRDM1, PTCH1, PTEN, PTPN11, PTPRD, PTPRT, RAD50, RAD51C, RAD52, RAD54L, RAF1, RB1, RBM10, RECQL4, REL, RET, RICTOR, RIT1, RNF43, ROS1, RPTOR, SDHA, SDHB, SDHC, SETD2, SF3B1, SMAD2, SMAD3, SMAD4, SMARCA4, SMARCB1, SMO, SOX2, SOX9, SPEN, SPOP, SRC, STAG2, STK11, SUZ12, SYK, TBX3, TERT, TET1, TET2, TGFBR2, TNFAIP3, TNFRSF14, TSC1, TSC2, TSHR, U2AF1, VHL, WT1, XPO1, AMER1, B2M, BARD1, BCL2L1, BCORL1, CALR, CBFB, CDH1, CDKN1B, CDKN2B, CDKN2C, CEBPA, CHD4, CNTNAP5, CRLF2, CSF3R, CSMD3, CXCR4, CYLD, EED, EPCAM, EPHA2, EPHA7, ERCC1, ERCC4, ERRFI1, ETV4, EWSR1, EZH2, FAM175A, FAM46C, FANCB, FANCG, FANCI, FGF23, FGF6, FOXA1, FOXL2, GATA6, GLI1, GNA11, GNAQ, GREM1, H3F3A, HSP90AA1, INHBA, KMT2A, KMT2B, KMT2C, KMT2D, LMO1, LRP1B, LYN, MAPK1, MDC 1.00, MSH3, MST1R, MUTYH, MYD88, NCOA3, NFKBIA, NUP93, PGR, PIK3CB, PLCG2, POLD1, PREX2, PRKAR1A, PRKCI, PRSS1, RAC1, RAD21, RAD51, RARA, RECQL, RHBDF2, RHOA, RUNX1, SDHAF2, SDHD, SETBP1, SH2B3, SLX4, SOCS1, SRSF2, STAT3, SUFU, TMEM127, TMPRSS2, TOP 1.00, TP53, TPMT, UGT1A1, VEGFA, WRN, YAP1, YES1, ACVR1, ARID5B, ASXL2, BBC3, BUB1, CADM2, CBLB, CD79A, CDKN1C, CTLA4, CYP19A1, DCUN1D1, DDX3X, DDX51, DIS3, DNMT1, DNMT3B, DPYD, DYNC2H1, E2F3, EGFL7, ESR2, EXT1, EXT2, FANCL, FGF10, FGF7, FOXM1, FOXP2, GEN1, GPRIN2, H3F3C, HDAC2, HIF1A, HIST1H1C, HIST1H2BD, HIST1H3B, HMGA2, HOXB13, ICOSLG, IFNGR1, IFNL2, IGF1, INPP4A, INSR, IRS1, KRT14, LRIG3, MAP3K13, MAPK3, MAPKBP1, MKI67, MLH3, MYCL, NAB2, NCOR1, NOTCH4, NR4A3, NRG1, NUTM1, PAK7, PALLD, PAX3, PAX8, PDCD1LG2, PHOX2B, PIK3C2G, PIK3C3, PPP2R2A, PRKACA, PTN, PTPRS, PZP, RAD51B, RAD51D, RASA1, RHEB, RIF1, ROBO2, RPS6KA4, RRAS2, RSF1, SBDS, SHQ1, SOX17, SPINK1, SS18, SYNE1, TGFBR1, TP63, TRAF7, TSPAN31, TYMS, VTCN1, WAS, XIAP, XRCC1, ZBTB16, ZNF367, ABCB1, ABCC1, ACTL6A, ADH1B, AGO2, ALDH2, ALOX12B, ANK2, ANKRD11, ANXA5, APCDD1, APOB, AREG, ARFRP1, ASNS, ASPH, ASTN2, BCL2L2, BCR, BIRC3, BRD2, BRINP3, BTG1, C8ORF34, CACNA1C, CAMTA1, CAPN2, CARM1, CASP7, CCL18, CD276, CD74, CDA, CDH18, CENPA, CFD, CHD2, CNTNAP2, COL22A1, CSDE1, CUL4A, CUL4B, CYP17A1, CYP1A1, CYP1B1, CYP2C8, CYP2D6, CYP 20.00, CYP3A4, CYP3A5, CYSLTR2, DDIT3, DNAJB1, DROSHA, DSCAM, EGF, EIF1AX, ELAC2, ELF3, EML4, EMSY, EPAS1, EREG, FAM123B, FAM58A, FANCD2, FANCE, FANCM, FAT3, FBN3, FGF1, FGF12, FGF14, FGF2, FGF5, FGF8, FGF9, FN1, FOXK2, FRS2, FUS, FYN, GAB2, GALNT12, GALNT14, GATA4, GEMIN6, GGH, GK5, GLIPR1, GLRX, GMEB1, GPER1, GPX5, GRID1, GSTA1, GSTP1, H3F3B, HDAC9, HFE2, HIST1H3D, HIST1H3G, HIST1H3I, HIST3H3, HLA-A, HLA-B, HNF1B, HSD17B3, HSD3B2, HTR1A, ID3, IFNLR1, IL10, INHA, INPPL1, KAT6A, KCNJ5, KEL, KIF1B, KIF5B, KLHL1, KLHL6, KLLN, KNSTRN, KRT15, KRT5, LARP4, LBR, LRFN5, LRP2, LRRC4C, LRRK2, LZTR1, MAGI2, MALT1, MGA, MKRN3, MLL3, MSI2, MTHFR, MTR, MTUS1, MYB, MYCL1, NAT2, NAV3, NCOA1, NEGR1, NKX3-1, NOS3, NOVA1, NQO1, NTHL1, NUF2, PAK3, PAPPA2, PARP2, PAX7, PCBP1, PDGFB, PDHA2, PDYN, PIK3C2B, PIK3R3, PLAUR, PLIN2, PLK2, PMAIP1, PNRC1, POM121L12, PPARG, PPIB, PPM1D, PPP4R2, PPP6C, PRKACB, PRKD1, PRKDC, PSME2, PXDNL, RAC2, RANBP2, RECK, RFWD2, RILP, RNASEL, RP1L1, RPL5, RPS26, RPS6KB1, RPS6KB2, RRAS, RRM1, RUNX1T1, RXRA, RYBP, RYR2, SCN10A, SELL, SEMA3C, SESN1, SETD7, SH2D1A, SHOC2, SHOX, SLC19A1, SLC22A2, SLC29A1, SLC34A2, SLCO1B1, SLCO1B3, SLIT2, SLITRK1, SLITRK2, SLITRK3, SMARCD1, SNCAIP, SOCS6, SOD2, SOS 1, SOX10, SPTA1, SRD5A2, STAT5B, STK40, STRN, SULT1A1, TAF1, TAP1, TCF3, TCF7L2, TEK, TERC, TFE3, TLR4, TNFRSF19, TNFSF8, TNR, TP53BP1, TRAF1, TRIM58, TUBB1, UPF1, WHSC1, WHSC1L1, WISP3, XIRP2, XPC, XRCC3, ZFHX3, ZFHX4, ZIC4, ZNF423, ZNF703, ZNF717, ZNF750, ZNF831, ZRSR2 |
| 464 | 16 | ABL1, ACTL6A, ACVR1, ADH1B, AKT1, AKT2, AKT3, ALDH2, ALK, AMER1, ANXA5, APC, AR, ARAF, AREG, ARID1A, ARID1B, ARID2, ASNS, ASPH, ASXL1, ATM, ATR, ATRX, AURKA, AURKB, AXIN1, AXIN2, AXL, B2M, BAP1, BARD1, BCL2, BCL2L1, BCL2L11, BCL6, BCOR, BCORL1, BLM, BMPR1A, BRAF, BRCA1, BRCA2, BRD2, BRD4, BRIP1, BTK, BUB1, CACNA1C, CADM2, CALR, CAMTA1, CAPN2, CARD11, CASP8, CBFB, CBL, CBLB, CCL18, CCND1, CCND2, CCND3, CCNE1, CD274, CD79A, CD79B, CDC73, CDH1, CDK12, CDK4, CDK6, CDK8, CDKN1A, CDKN1B, CDKN1C, CDKN2A, CDKN2B, CDKN2C, CEBPA, CFD, CHD4, CHEK1, CHEK2, CIC, CNTNAP5, CREBBP, CRKL, CRLF2, CSF1R, CSF3R, CSMD3, CTCF, CTNNB1, CUL3, CXCR4, CYLD, CYP19A1, DAXX, DDIT3, DDR2, DDX3X, DDX51, DICER1, DNMT3A, DOT1L, EED, EGFR, ELAC2, EP300, EPCAM, EPHA2, EPHA3, EPHA5, EPHA7, EPHB1, ERBB2, ERBB3, ERBB4, ERCC1, ERCC2, ERCC3, ERCC4, ERCC5, EREG, ERG, ERRFI1, ESR1, ESR2, ETV1, ETV4, ETV6, EWSR1, EXT1, EXT2, EZH2, FAM175A, FAM46C, FANCA, FANCB, FANCC, FANCG, FANCI, FANCL, FAT1, FBN3, FBXW7, FGF1, FGF10, FGF19, FGF2, FGF23, FGF3, FGF4, FGF5, FGF6, FGF7, FGF8, FGF9, FGFR1, FGFR2, FGFR3, FGFR4, FH, FLCN, FLT1, FLT3, FLT4, FOXA1, FOXK2, FOXL2, FOXM1, FOXP1, FOXP2, FUBP1, FUS, GAB2, GATA1, GATA2, GATA3, GATA6, GEMIN6, GEN1, GK5, GLI1, GLIPR1, GLRX, GMEB1, GNA11, GNAQ, GNAS, GPER1, GPRIN2, GREM1, GRIN2A, GSK3B, H3F3A, HDAC2, HFE2, HGF, HIF1A, HMGA2, HNF1A, HNF1B, HOXB13, HRAS, HSD17B3, HSD3B2, HSP90AA1, IDH1, IDH2, IFNL2, IFNLR1, IGF1R, IGF2, IKBKE, IKZF1, IL7R, INHBA, INPP4B, IRF4, IRS2, JAK1, JAK2, JAK3, JUN, KCNJ5, KDM5A, KDM5C, KDM6A, KDR, KEAP1, KIF1B, KIT, KLF4, KLLN, KMT2A, KMT2B, KMT2C, KMT2D, KRAS, KRT14, KRT15, KRT5, LARP4, LATS1, LATS2, LBR, LMO1, LRIG3, LRP1B, LYN, MAP2K1, MAP2K2, MAP2K4, MAP3K1, MAPK1, MAPK3, MAPKBP1, MAX, MCL1, MDC1, MDM2, MDM4, MED12, MEF2B, MEN1, MET, MITF, MKI67, MLH1, MLH3, MPL, MRE11A, MSH2, MSH3, MSH6, MST1R, MTOR, MTUS1, MUTYH, MYC, MYCL, MYCN, MYD88, MYOD1, NAB2, NBN, NCOA3, NF1, NF2, NFE2L2, NFKBIA, NKX2-1, NOTCH1, NOTCH2, NOTCH3, NOVA1, NPM1, NR4A3, NRAS, NRG1, NSD1, NTRK1, NTRK2, NTRK3, NUP93, NUTM1, PAK1, PALB2, PALLD, PARK2, PARP1, PAX3, PAX5, PAX7, PAX8, PBRM1, PCBP1, PDCD1, PDCD1LG2, PDGFB, PDGFRA, PDGFRB, PDPK1, PGR, PIK3CA, PIK3CB, PIK3CD, PIK3CG, PIK3R1, PIK3R2, PIM1, PLAUR, PLCG2, PLIN2, PMS1, PMS2, POLD1, POLE, PPIB, PPP2R1A, PPP2R2A, PRDM1, PREX2, PRKACA, PRKACB, PRKAR1A, PRKCI, PRSS1, PSME2, PTCH1, PTEN, PTN, PTPN11, PTPRD, PTPRT, PZP, RAC1, RAD21, RAD50, RAD51, RAD51B, RAD51C, RAD51D, RAD52, RAD54L, RAF1, RARA, RB1, RBM10, RECK, RECQL, RECQL4, REL, RET, RHBDF2, RHEB, RHOA, RICTOR, RIF1, RILP, RIT1, RNASEL, RNF43, ROBO2, ROS1, RPS6KB1, RPTOR, RSF1, RUNX1, SBDS, SDHA, SDHAF2, SDHB, SDHC, SDHD, SELL, SETBP1, SETD2, SETD7, SF3B1, SH2B3, SHOX, SLX4, SMAD2, SMAD3, SMAD4, SMARCA4, SMARCB1, SMO, SOCS1, SOCS6, SOX2, SOX9, SPEN, SPINK1, SPOP, SRC, SRD5A2, SRSF2, SS18, STAG2, STAT3, STK11, SUFU, SULT1A1, SUZ12, SYK, SYNE1, TBX3, TERT, TET1, TET2, TFE3, TGFBR2, TMEM127, TMPRSS2, TNFAIP3, TNFRSF14, TNFRSF19, TNFSF8, TOP1, TP53, TPMT, TRAF1, TSC1, TSC2, TSHR, TSPAN31, TYMS, U2AF1, UGT1A1, VEGFA, VHL, WAS, WRN, WT1, XPO1, YAP1, YES1, ZBTB16, ZNF367, ZNF717, ZNF750 |
| 425 | 45 | ABL1, AKT1, AKT2, AKT3, ALK, APC, AR, ARAF, ARID1A, ARID1B, ARID2, ASXL1, ATM, ATR, ATRX, AURKA, AURKB, AXIN1, AXIN2, AXL, BAP1, BCL2, BCL2L11, BCL6, BCOR, BLM, BMPR1A, BRAF, BRCA1, BRCA2, BRD4, BRIP1, BTK, CARD11, CASP8, CBL, CCND1, CCND2, CCND3, CCNE1, CD274, CD79B, CDC73, CDK12, CDK4, CDK6, CDK8, CDKN1A, CDKN2A, CHEK1, CHEK2, CIC, CREBBP, CRKL, CSF1R, CTCF, CTNNB1, CUL3, DAXX, DDR2, DICER1, DNMT3A, DOT1L, EGFR, EP300, EPHA3, EPHA5, EPHB1, ERBB2, ERBB3, ERBB4, ERCC2, ERCC3, ERCC5, ERG, ESR1, ETV1, ETV6, FANCA, FANCC, FAT1, FBXW7, FGF19, FGF3, FGF4, FGFR1, FGFR2, FGFR3, FGFR4, FH, FLCN, FLT1, FLT3, FLT4, FOXP1, FUBP1, GATA1, GATA2, GATA3, GNAS, GRIN2A, GSK3B, HGF, HNF1A, HRAS, IDH1, IDH2, IGF1R, IGF2, IKBKE, IKZF1, IL7R, INPP4B, IRF4, IRS2, JAK1, JAK2, JAK3, JUN, KDM5A, KDM5C, KDM6A, KDR, KEAP1, KIT, KLF4, KRAS, LATS1, LATS2, MAP2K1, MAP2K2, MAP2K4, MAP3K1, MAX, MCL1, MDM2, MDM4, MED12, MEF2B, MEN1, MET, MITF, MLH1, MPL, MRE11A, MSH2, MSH6, MTOR, MYC, MYCN, MYOD1, NBN, NF1, NF2, NFE2L2, NKX2-1, NOTCH1, NOTCH2, NOTCH3, NPM1, NRAS, NSD1, NTRK1, NTRK2, NTRK3, PAK1, PALB2, PARK2, PARP1, PAX5, PBRM1, PDCD1, PDGFRA, PDGFRB, PDPK1, PIK3CA, PIK3CD, PIK3CG, PIK3R1, PIK3R2, PIM1, PMS1, PMS2, POLE, PPP2R1A, PRDM1, PTCH1, PTEN, PTPN11, PTPRD, PTPRT, RAD50, RAD51C, RAD52, RAD54L, RAF1, RB1, RBM10, RECQL4, REL, RET, RICTOR, RIT1, RNF43, ROS1, RPTOR, SDHA, SDHB, SDHC, SETD2, SF3B1, SMAD2, SMAD3, SMAD4, SMARCA4, SMARCB1, SMO, SOX2, SOX9, SPEN, SPOP, SRC, STAG2, STK11, SUZ12, SYK, TBX3, TERT, TET1, TET2, TGFBR2, TNFAIP3, TNFRSF14, TSC1, TSC2, TSHR, U2AF1, VHL, WT1, XPO1, AMER1, B2M, BARD1, BCL2L1, BCORL1, CALR, CBFB, CDH1, CDKN1B, CDKN2B, CDKN2C, CEBPA, CHD4, CNTNAP5, CRLF2, CSF3R, CSMD3, CXCR4, CYLD, EED, EPCAM, EPHA2, EPHA7, ERCC1, ERCC4, ERRFI1, ETV4, EWSR1, EZH2, FAM175A, FAM46C, FANCB, FANCG, FANCI, FGF23, FGF6, FOXA1, FOXL2, GATA6, GLI1, GNA11, GNAQ, GREM1, H3F3A, HSP90AA1, INHBA, KMT2A, KMT2B, KMT2C, KMT2D, LMO1, LRP1B, LYN, MAPK1, MDC 1.00, MSH3, MST1R, MUTYH, MYD88, NCOA3, NFKBIA, NUP93, PGR, PIK3CB, PLCG2, POLD1, PREX2, PRKAR1A, PRKCI, PRSS1, RAC1, RAD21, RAD51, RARA, RECQL, RHBDF2, RHOA, RUNX1, SDHAF2, SDHD, SETBP1, SH2B3, SLX4, SOCS1, SRSF2, STAT3, SUFU, TMEM127, TMPRSS2, TOP 1.00, TP53, TPMT, UGT1A1, VEGFA, WRN, YAP1, YES1, ACVR1, ARID5B, ASXL2, BBC3, BUB1, CADM2, CBLB, CD79A, CDKN1C, CTLA4, CYP19A1, DCUN1D1, DDX3X, DDX51, DIS3, DNMT1, DNMT3B, DPYD, DYNC2H1, E2F3, EGFL7, ESR2, EXT1, EXT2, FANCL, FGF10, FGF7, FOXM1, FOXP2, GEN1, GPRIN2, H3F3C, HDAC2, HIF1A, HIST1H1C, HIST1H2BD, HIST1H3B, HMGA2, HOXB13, ICOSLG, IFNGR1, IFNL2, IGF1, INPP4A, INSR, IRS1, KRT14, LRIG3, MAP3K13, MAPK3, MAPKBP1, MKI67, MLH3, MYCL, NAB2, NCOR1, NOTCH4, NR4A3, NRG1, NUTM1, PAK7, PALLD, PAX3, PAX8, PDCD1LG2, PHOX2B, PIK3C2G, PIK3C3, PPP2R2A, PRKACA, PTN, PTPRS, PZP, RAD51B, RAD51D, RASA1, RHEB, RIF1, ROBO2, RPS6KA4, RRAS2, RSF1, SBDS, SHQ1, SOX17, SPINK1, SS18, SYNE1, TGFBR1, TP63, TRAF7, TSPAN31, TYMS, VTCN1, WAS, XIAP, XRCC1, ZBTB16, ZNF367 |
| 410 | 24 | ABL1, AKT1, AKT2, AKT3, ALK, APC, AR, ARAF, ARID1A, ARID1B, ARID2, ASXL1, ATM, ATR, ATRX, AURKA, AURKB, AXIN1, AXIN2, AXL, BAP1, BCL2, BCL2L11, BCL6, BCOR, BLM, BMPR1A, BRAF, BRCA1, BRCA2, BRD4, BRIP1, BTK, CARD11, CASP8, CBL, CCND1, CCND2, CCND3, CCNE1, CD274, CD79B, CDC73, CDK12, CDK4, CDK6, CDK8, CDKN1A, CDKN2A, CHEK1, CHEK2, CIC, CREBBP, CRKL, CSF1R, CTCF, CTNNB1, CUL3, DAXX, DDR2, DICER1, DNMT3A, DOT1L, EGFR, EP300, EPHA3, EPHA5, EPHB1, ERBB2, ERBB3, ERBB4, ERCC2, ERCC3, ERCC5, ERG, ESR1, ETV1, ETV6, FANCA, FANCC, FAT1, FBXW7, FGF19, FGF3, FGF4, FGFR1, FGFR2, FGFR3, FGFR4, FH, FLCN, FLT1, FLT3, FLT4, FOXP1, FUBP1, GATA1, GATA2, GATA3, GNAS, GRIN2A, GSK3B, HGF, HNF1A, HRAS, IDH1, IDH2, IGF1R, IGF2, IKBKE, IKZF1, IL7R, INPP4B, IRF4, IRS2, JAK1, JAK2, JAK3, JUN, KDM5A, KDM5C, KDM6A, KDR, KEAP1, KIT, KLF4, KRAS, LATS1, LATS2, MAP2K1, MAP2K2, MAP2K4, MAP3K1, MAX, MCL1, MDM2, MDM4, MED12, MEF2B, MEN1, MET, MITF, MLH1, MPL, MRE11A, MSH2, MSH6, MTOR, MYC, MYCN, MYOD1, NBN, NF1, NF2, NFE2L2, NKX2-1, NOTCH1, NOTCH2, NOTCH3, NPM1, NRAS, NSD1, NTRK1, NTRK2, NTRK3, PAK1, PALB2, PARK2, PARP1, PAX5, PBRM1, PDCD1, PDGFRA, PDGFRB, PDPK1, PIK3CA, PIK3CD, PIK3CG, PIK3R1, PIK3R2, PIM1, PMS1, PMS2, POLE, PPP2R1A, PRDM1, PTCH1, PTEN, PTPN11, PTPRD, PTPRT, RAD50, RAD51C, RAD52, RAD54L, RAF1, RB1, RBM10, RECQL4, REL, RET, RICTOR, RIT1, RNF43, ROS1, RPTOR, SDHA, SDHB, SDHC, SETD2, SF3B1, SMAD2, SMAD3, SMAD4, SMARCA4, SMARCB1, SMO, SOX2, SOX9, SPEN, SPOP, SRC, STAG2, STK11, SUZ12, SYK, TBX3, TERT, TET1, TET2, TGFBR2, TNFAIP3, TNFRSF14, TSC1, TSC2, TSHR, U2AF1, VHL, WT1, XPO1, AMER1, B2M, BARD1, BCL2L1, BCORL1, CALR, CBFB, CDH1, CDKN1B, CDKN2B, CDKN2C, CEBPA, CHD4, CNTNAP5, CRLF2, CSF3R, CSMD3, CXCR4, CYLD, EED, EPCAM, EPHA2, EPHA7, ERCC1, ERCC4, ERRFI1, ETV4, EWSR1, EZH2, FAM175A, FAM46C, FANCB, FANCG, FANCI, FGF23, FGF6, FOXA1, FOXL2, GATA6, GLI1, GNA11, GNAQ, GREM1, H3F3A, HSP90AA1, INHBA, KMT2A, KMT2B, KMT2C, KMT2D, LMO1, LRP1B, LYN, MAPK1, MDC 1.00, MSH3, MST1R, MUTYH, MYD88, NCOA3, NFKBIA, NUP93, PGR, PIK3CB, PLCG2, POLD1, PREX2, PRKAR1A, PRKCI, PRSS1, RAC1, RAD21, RAD51, RARA, RECQL, RHBDF2, RHOA, RUNX1, SDHAF2, SDHD, SETBP1, SH2B3, SLX4, SOCS1, SRSF2, STAT3, SUFU, TMEM127, TMPRSS2, TOP 1.00, TP53, TPMT, UGT1A1, VEGFA, WRN, YAP1, YES1, ACVR1, ARID5B, ASXL2, BBC3, BUB1, CADM2, CBLB, CD79A, CDKN1C, CTLA4, CYP19A1, DCUN1D1, DDX3X, DDX51, DIS3, DNMT1, DNMT3B, DPYD, DYNC2H1, E2F3, EGFL7, ESR2, EXT1, EXT2, FANCL, FGF10, FGF7, FOXM1, FOXP2, GEN1, GPRIN2, H3F3C, HDAC2, HIF1A, HIST1H1C, HIST1H2BD, HIST1H3B, HMGA2, HOXB13, ICOSLG, IFNGR1, IFNL2, IGF1, INPP4A, INSR, IRS1, KRT14, LRIG3, MAP3K13, MAPK3, MAPKBP1, MKI67, MLH3, MYCL, NAB2, NCOR1, NOTCH4, NR4A3, NRG1, NUTM1, PAK7, PALLD, PAX3, PAX8, PDCD1LG2, PHOX2B, PIK3C2G, PIK3C3, PPP2R2A, PRKACA, PTN, PTPRS, PZP, RAD51B, RAD51D, RASA1, RHEB, RIF1, ROBO2, RPS6KA4, RRAS2, RSF1, SBDS, SHQ1, SOX17, SPINK1, SS18, SYNE1, TGFBR1, TP63, TRAF7, TSPAN31, TYMS, VTCN1, WAS, XIAP, XRCC1, ZBTB16, ZNF367 |
| 409 | 17 | ABL1, AKT1, AKT2, AKT3, ALK, APC, AR, ARAF, ARID1A, ARID1B, ARID2, ASXL1, ATM, ATR, ATRX, AURKA, AURKB, AXIN1, AXIN2, AXL, BAP1, BCL2, BCL2L11, BCL6, BCOR, BLM, BMPR1A, BRAF, BRCA1, BRCA2, BRD4, BRIP1, BTK, CARD11, CASP8, CBL, CCND1, CCND2, CCND3, CCNE1, CD274, CD79B, CDC73, CDK12, CDK4, CDK6, CDK8, CDKN1A, CDKN2A, CHEK1, CHEK2, CIC, CREBBP, CRKL, CSF1R, CTCF, CTNNB1, CUL3, DAXX, DDR2, DICER1, DNMT3A, DOT1L, EGFR, EP300, EPHA3, EPHA5, EPHB1, ERBB2, ERBB3, ERBB4, ERCC2, ERCC3, ERCC5, ERG, ESR1, ETV1, ETV6, FANCA, FANCC, FAT1, FBXW7, FGF19, FGF3, FGF4, FGFR1, FGFR2, FGFR3, FGFR4, FH, FLCN, FLT1, FLT3, FLT4, FOXP1, FUBP1, GATA1, GATA2, GATA3, GNAS, GRIN2A, GSK3B, HGF, HNF1A, HRAS, IDH1, IDH2, IGF1R, IGF2, IKBKE, IKZF1, IL7R, INPP4B, IRF4, IRS2, JAK1, JAK2, JAK3, JUN, KDM5A, KDM5C, KDM6A, KDR, KEAP1, KIT, KLF4, KRAS, LATS1, LATS2, MAP2K1, MAP2K2, MAP2K4, MAP3K1, MAX, MCL1, MDM2, MDM4, MED12, MEF2B, MEN1, MET, MITF, MLH1, MPL, MRE11A, MSH2, MSH6, MTOR, MYC, MYCN, MYOD1, NBN, NF1, NF2, NFE2L2, NKX2-1, NOTCH1, NOTCH2, NOTCH3, NPM1, NRAS, NSD1, NTRK1, NTRK2, NTRK3, PAK1, PALB2, PARK2, PARP1, PAX5, PBRM1, PDCD1, PDGFRA, PDGFRB, PDPK1, PIK3CA, PIK3CD, PIK3CG, PIK3R1, PIK3R2, PIM1, PMS1, PMS2, POLE, PPP2R1A, PRDM1, PTCH1, PTEN, PTPN11, PTPRD, PTPRT, RAD50, RAD51C, RAD52, RAD54L, RAF1, RB1, RBM10, RECQL4, REL, RET, RICTOR, RIT1, RNF43, ROS1, RPTOR, SDHA, SDHB, SDHC, SETD2, SF3B1, SMAD2, SMAD3, SMAD4, SMARCA4, SMARCB1, SMO, SOX2, SOX9, SPEN, SPOP, SRC, STAG2, STK11, SUZ12, SYK, TBX3, TERT, TET1, TET2, TGFBR2, TNFAIP3, TNFRSF14, TSC1, TSC2, TSHR, U2AF1, VHL, WT1, XPO1, AMER1, B2M, BARD1, BCL2L1, BCORL1, CALR, CBFB, CDH1, CDKN1B, CDKN2B, CDKN2C, CEBPA, CHD4, CNTNAP5, CRLF2, CSF3R, CSMD3, CXCR4, CYLD, EED, EPCAM, EPHA2, EPHA7, ERCC1, ERCC4, ERRFI1, ETV4, EWSR1, EZH2, FAM175A, FAM46C, FANCB, FANCG, FANCI, FGF23, FGF6, FOXA1, FOXL2, GATA6, GLI1, GNA11, GNAQ, GREM1, H3F3A, HSP90AA1, INHBA, KMT2A, KMT2B, KMT2C, KMT2D, LMO1, LRP1B, LYN, MAPK1, MDC 1.00, MSH3, MST1R, MUTYH, MYD88, NCOA3, NFKBIA, NUP93, PGR, PIK3CB, PLCG2, POLD1, PREX2, PRKAR1A, PRKCI, PRSS1, RAC1, RAD21, RAD51, RARA, RECQL, RHBDF2, RHOA, RUNX1, SDHAF2, SDHD, SETBP1, SH2B3, SLX4, SOCS1, SRSF2, STAT3, SUFU, TMEM127, TMPRSS2, TOP 1.00, TP53, TPMT, UGT1A1, VEGFA, WRN, YAP1, YES1, ACVR1, ARID5B, ASXL2, BBC3, BUB1, CADM2, CBLB, CD79A, CDKN1C, CTLA4, CYP19A1, DCUN1D1, DDX3X, DDX51, DIS3, DNMT1, DNMT3B, DPYD, DYNC2H1, E2F3, EGFL7, ESR2, EXT1, EXT2, FANCL, FGF10, FGF7, FOXM1, FOXP2, GEN1, GPRIN2, H3F3C, HDAC2, HIF1A, HIST1H1C, HIST1H2BD, HIST1H3B, HMGA2, HOXB13, ICOSLG, IFNGR1, IFNL2, IGF1, INPP4A, INSR, IRS1, KRT14, LRIG3, MAP3K13, MAPK3, MAPKBP1, MKI67, MLH3, MYCL, NAB2, NCOR1, NOTCH4, NR4A3, NRG1, NUTM1, PAK7, PALLD, PAX3, PAX8, PDCD1LG2, PHOX2B, PIK3C2G, PIK3C3, PPP2R2A, PRKACA, PTN, PTPRS, PZP, RAD51B, RAD51D, RASA1, RHEB, RIF1, ROBO2, RPS6KA4, RRAS2, RSF1, SBDS, SHQ1, SOX17, SPINK1, SS18, SYNE1, TGFBR1, TP63, TRAF7, TSPAN31, TYMS, VTCN1, WAS, XIAP, XRCC1, ZBTB16, ZNF367 |
| 400 | 60 | ABL1, AKT1, AKT2, AKT3, ALK, APC, AR, ARAF, ARID1A, ARID1B, ARID2, ASXL1, ATM, ATR, ATRX, AURKA, AURKB, AXIN1, AXIN2, AXL, BAP1, BCL2, BCL2L11, BCL6, BCOR, BLM, BMPR1A, BRAF, BRCA1, BRCA2, BRD4, BRIP1, BTK, CARD11, CASP8, CBL, CCND1, CCND2, CCND3, CCNE1, CD274, CD79B, CDC73, CDK12, CDK4, CDK6, CDK8, CDKN1A, CDKN2A, CHEK1, CHEK2, CIC, CREBBP, CRKL, CSF1R, CTCF, CTNNB1, CUL3, DAXX, DDR2, DICER1, DNMT3A, DOT1L, EGFR, EP300, EPHA3, EPHA5, EPHB1, ERBB2, ERBB3, ERBB4, ERCC2, ERCC3, ERCC5, ERG, ESR1, ETV1, ETV6, FANCA, FANCC, FAT1, FBXW7, FGF19, FGF3, FGF4, FGFR1, FGFR2, FGFR3, FGFR4, FH, FLCN, FLT1, FLT3, FLT4, FOXP1, FUBP1, GATA1, GATA2, GATA3, GNAS, GRIN2A, GSK3B, HGF, HNF1A, HRAS, IDH1, IDH2, IGF1R, IGF2, IKBKE, IKZF1, IL7R, INPP4B, IRF4, IRS2, JAK1, JAK2, JAK3, JUN, KDM5A, KDM5C, KDM6A, KDR, KEAP1, KIT, KLF4, KRAS, LATS1, LATS2, MAP2K1, MAP2K2, MAP2K4, MAP3K1, MAX, MCL1, MDM2, MDM4, MED12, MEF2B, MEN1, MET, MITF, MLH1, MPL, MRE11A, MSH2, MSH6, MTOR, MYC, MYCN, MYOD1, NBN, NF1, NF2, NFE2L2, NKX2-1, NOTCH1, NOTCH2, NOTCH3, NPM1, NRAS, NSD1, NTRK1, NTRK2, NTRK3, PAK1, PALB2, PARK2, PARP1, PAX5, PBRM1, PDCD1, PDGFRA, PDGFRB, PDPK1, PIK3CA, PIK3CD, PIK3CG, PIK3R1, PIK3R2, PIM1, PMS1, PMS2, POLE, PPP2R1A, PRDM1, PTCH1, PTEN, PTPN11, PTPRD, PTPRT, RAD50, RAD51C, RAD52, RAD54L, RAF1, RB1, RBM10, RECQL4, REL, RET, RICTOR, RIT1, RNF43, ROS1, RPTOR, SDHA, SDHB, SDHC, SETD2, SF3B1, SMAD2, SMAD3, SMAD4, SMARCA4, SMARCB1, SMO, SOX2, SOX9, SPEN, SPOP, SRC, STAG2, STK11, SUZ12, SYK, TBX3, TERT, TET1, TET2, TGFBR2, TNFAIP3, TNFRSF14, TSC1, TSC2, TSHR, U2AF1, VHL, WT1, XPO1, AMER1, B2M, BARD1, BCL2L1, BCORL1, CALR, CBFB, CDH1, CDKN1B, CDKN2B, CDKN2C, CEBPA, CHD4, CNTNAP5, CRLF2, CSF3R, CSMD3, CXCR4, CYLD, EED, EPCAM, EPHA2, EPHA7, ERCC1, ERCC4, ERRFI1, ETV4, EWSR1, EZH2, FAM175A, FAM46C, FANCB, FANCG, FANCI, FGF23, FGF6, FOXA1, FOXL2, GATA6, GLI1, GNA11, GNAQ, GREM1, H3F3A, HSP90AA1, INHBA, KMT2A, KMT2B, KMT2C, KMT2D, LMO1, LRP1B, LYN, MAPK1, MDC 1.00, MSH3, MST1R, MUTYH, MYD88, NCOA3, NFKBIA, NUP93, PGR, PIK3CB, PLCG2, POLD1, PREX2, PRKAR1A, PRKCI, PRSS1, RAC1, RAD21, RAD51, RARA, RECQL, RHBDF2, RHOA, RUNX1, SDHAF2, SDHD, SETBP1, SH2B3, SLX4, SOCS1, SRSF2, STAT3, SUFU, TMEM127, TMPRSS2, TOP 1.00, TP53, TPMT, UGT1A1, VEGFA, WRN, YAP1, YES1, ACVR1, ARID5B, ASXL2, BBC3, BUB1, CADM2, CBLB, CD79A, CDKN1C, CTLA4, CYP19A1, DCUN1D1, DDX3X, DDX51, DIS3, DNMT1, DNMT3B, DPYD, DYNC2H1, E2F3, EGFL7, ESR2, EXT1, EXT2, FANCL, FGF10, FGF7, FOXM1, FOXP2, GEN1, GPRIN2, H3F3C, HDAC2, HIF1A, HIST1H1C, HIST1H2BD, HIST1H3B, HMGA2, HOXB13, ICOSLG, IFNGR1, IFNL2, IGF1, INPP4A, INSR, IRS1, KRT14, LRIG3, MAP3K13, MAPK3, MAPKBP1, MKI67, MLH3, MYCL, NAB2, NCOR1, NOTCH4, NR4A3, NRG1, NUTM1, PAK7, PALLD, PAX3, PAX8, PDCD1LG2, PHOX2B, PIK3C2G, PIK3C3, PPP2R2A, PRKACA, PTN, PTPRS, PZP, RAD51B, RAD51D, RASA1, RHEB, RIF1, ROBO2, RPS6KA4, RRAS2, RSF1, SBDS, SHQ1, SOX17, SPINK1, SS18, SYNE1, TGFBR1, TP63, TRAF7, TSPAN31, TYMS, VTCN1, WAS, XIAP, XRCC1, ZBTB16, ZNF367 |
| 168 | 17 | ABL1, AKT1, AKT2, AKT3, ALK, APC, AR, ARAF, ARID1A, ARID1B, ARID2, ASXL1, ATM, ATR, ATRX, AURKA, AURKB, AXIN1, AXIN2, AXL, BAP1, BCL2, BCL2L11, BCL6, BCOR, BLM, BMPR1A, BRAF, BRCA1, BRCA2, BRD4, BRIP1, BTK, CARD11, CASP8, CBL, CCND1, CCND2, CCND3, CCNE1, CD274, CD79B, CDC73, CDK12, CDK4, CDK6, CDK8, CDKN1A, CDKN2A, CHEK1, CHEK2, CIC, CREBBP, CRKL, CSF1R, CTCF, CTNNB1, CUL3, DAXX, DDR2, DICER1, DNMT3A, DOT1L, EGFR, EP300, EPHA3, EPHA5, EPHB1, ERBB2, ERBB3, ERBB4, ERCC2, ERCC3, ERCC5, ERG, ESR1, ETV1, ETV6, FANCA, FANCC, FAT1, FBXW7, FGF19, FGF3, FGF4, FGFR1, FGFR2, FGFR3, FGFR4, FH, FLCN, FLT1, FLT3, FLT4, FOXP1, FUBP1, GATA1, GATA2, GATA3, GNAS, GRIN2A, GSK3B, HGF, HNF1A, HRAS, IDH1, IDH2, IGF1R, IGF2, IKBKE, IKZF1, IL7R, INPP4B, IRF4, IRS2, JAK1, JAK2, JAK3, JUN, KDM5A, KDM5C, KDM6A, KDR, KEAP1, KIT, KLF4, KRAS, LATS1, LATS2, MAP2K1, MAP2K2, MAP2K4, MAP3K1, MAX, MCL1, MDM2, MDM4, MED12, MEF2B, MEN1, MET, MITF, MLH1, MPL, MRE11A, MSH2, MSH6, MTOR, MYC, MYCN, MYOD1, NBN, NF1, NF2, NFE2L2, NKX2-1, NOTCH1, NOTCH2, NOTCH3, NPM1, NRAS, NSD1, NTRK1, NTRK2, NTRK3, PAK1, PALB2, PARK2, PARP1, PAX5, PBRM1, PDCD1, PDGFRA, PDGFRB, PDPK1, PIK3CA, PIK3CD, PIK3CG, PIK3R1, PIK3R2, PIM1, PMS1, PMS2, POLE, PPP2R1A, PRDM1, PTCH1, PTEN, PTPN11, PTPRD, PTPRT, RAD50, RAD51C, RAD52, RAD54L, RAF1, RB1, RBM10, RECQL4, REL, RET, RICTOR, RIT1, RNF43, ROS1, RPTOR, SDHA, SDHB, SDHC, SETD2, SF3B1, SMAD2, SMAD3, SMAD4, SMARCA4, SMARCB1, SMO, SOX2, SOX9, SPEN, SPOP, SRC, STAG2, STK11, SUZ12, SYK, TBX3, TERT, TET1, TET2, TGFBR2, TNFAIP3, TNFRSF14, TSC1, TSC2, TSHR, U2AF1, VHL, WT1, XPO1 |
| 53 | 49 | AKT1, AKT2, AKT3, ARID1A, ARID1B, ARID2, ASCL4, ATM, BRAF, CDKN2A, COBL, CREBBP, CTNNB1, CUL3, EGFR, EP300, EPHA7, ERBB2, ERBB3, FGFR1, FGFR2, FGFR3, FOXP2, HRAS, KEAP1, KMT2D, KRAS, MAP2K1, MET, MGA, MLL, NF1, NFE2L2, NOTCH1, NOTCH2, NRAS, PIK3CA, PTEN, RASA1, RB1, RBM10, RIT1, SETD2, SLIT2, SMAD4, SMARCA4, SOX2, STK11, TP53, TP63, TSC1, TSC2, U2AF1 |
| 52 | 61 | ABL1, AKT1, AKT3, ALK, AR, AXL, BRAF, CCND1, CDK4, CDK6, CTNNB1, DDR2, EGFR, ERBB2, ERBB3, ERBB4, ERG, ESR1, ETV1, ETV4, ETV5, FGFR1, FGFR2, FGFR3, FGFR4, GNA11, GNAQ, HRAS, IDH1, IDH2, JAK1, JAK2, JAK3, KIT, KRAS, MAP2K1, MAP2K2, MET, MTOR, MYC, MYCN, NRAS, NTRK1, NTRK2, NTRK3, PDGFRA, PIK3CA, PPARG, RAF1, RET, ROS1, SMO |
| 50 | 125 | ABL1, AKT1, ALK, APC, ATM, BRAF, CDH1, CDKN2A, CSF1R, CTNNB1, EGFR, ERBB2, ERBB4, EZH2, FBXW7, FGFR1, FGFR2, FGFR3, FLT3, GNA11, GNAQ, GNAS, HNF1A, HRAS, IDH1, IDH2, JAK2, JAK3, KDR, KIT, KRAS, MET, MLH1, MPL, NOTCH1, NPM1, NRAS, PDGFRA, PIK3CA, PTEN, PTPN11, RB1, RET, SMAD4, SMARCB1, SMO, SRC, STK11, TP53, VHL |
| 48/47 | 65 | ABL1, AKT1, ALK, APC, ATM, BRAF, CDH1, CDKN2A, CSF1R, CTNNB1, EGFR, ERBB2, ERBB4, FBXW7, FGFR1, FGFR2, FGFR3, FLT3, GNA11, GNAQ, GNAS, HNF1A, HRAS, IDH1, JAK2, JAK3, KDR, KIT, KRAS, MET, MLH1, MPL, NOTCH1, NPM1, NRAS, PDGFRA, PIK3CA, PTEN, PTPN11, RB1, RET, SMAD4, SMARCB1, SMO, SRC, STK11, TP53, VHL |
| 35 | 32 | AKT1, ALK, APC, ATM, BRAF, CDH1, CDKN2A, CSF1R, CTNNB1, EGFR, ERBB2, ERBB4, FBXW7, FGFR1, FGFR2, FGFR3, HNF1A, HRAS, IDH1, IDH2, KDR, KIT, KRAS, MET, NOTCH1, NRAS, PDGFRA, PIK3CA, RET, SMAD4, SMARCB1, SMO, SRC, STK11, TP53 |
| 22 | 107 | AKT1, ALK, BRAF, CTNNB1, DDR2, EGFR, ERBB2, ERBB4, FBX7, FGFR1, FGFR2, FGFR3, KRAS, MAP2K1, MET, NOTCH1, NRAS, PIK3CA, PTEN, SMAD4, STK11, TP53 |
| 20 | 67 | AKT1, ALK, BRAF, CDKN2A, CTNNB1, EGFR, ERBB2, HRAS, KIT, KRAS, MET, MTOR, NRAS, PDGFRA, PIK3CA, PTEN, PTGS2, RB1, STK11, TP53 |
| 10 | 42 | EGFR, ALK, ROS1, BRAF, RET, MET, ERBB2, KRAS, PIK3CA, TP53 |
| 8 | 30 | BRAF, EGFR, ERBB2, KRAS, NRAS, AKT1, PIK3CA, TP53 |
| 2 | 112 | EGFR, KRAS |
| 1 | 209 | EGFR |

This table presents the details of the subsampled panels used in the analysis. Panels were selected and arranged based on the ranked frequency of mutation detection, referencing commonly used commercial panels. All larger panels covered the smaller ranges adequately, ensuring the subsampling process was both feasible and reliable.

# Table S2: Prior Probability Distributions and Optimized Parameters for Sensitivity and Specificity Across Different Panels

| **panel** | **pi** | **N** | **n11** | **n10** | **n01** | **n00** | **sens1_alpha** | **sens1_beta** | **spec1_**  **alpha** | **spec1_**  **beta** | **sens2_**  **alpha** | **sens2_**  **beta** | **spec2_**  **alpha** | **spec2_**  **beta** |
| --- | --- | --- | --- | --- | --- | --- | --- | --- | --- | --- | --- | --- | --- | --- |
| **0-10** | 0.590 | 130 | 102 | 9 | 6 | 13 | 391.048 | 3.949 | 36.851 | 57.397 | 16.449 | 0.829 | 44.318 | 8.442 |
| **20-35** | 0.590 | 109 | 59 | 7 | 2 | 41 | 391.059 | 3.946 | 30.707 | 58.558 | 23.581 | 1.802 | 41.153 | 6.867 |
| **47-53** | 0.590 | 193 | 139 | 12 | 4 | 38 | 391.055 | 3.952 | 38.000 | 57.000 | 19.332 | 1.169 | 34.201 | 4.270 |
| **168-464** | 0.590 | 91 | 54 | 2 | 1 | 34 | 391.052 | 3.943 | 49.500 | 49.500 | 1092.725 | 32.635 | 36.964 | 5.184 |
| **500-WES** | 0.590 | 109 | 82 | 13 | 2 | 12 | 391.043 | 3.946 | 40.134 | 56.110 | 543.444 | 7.716 | 14.796 | 0.665 |
| **1** | 0.590 | 214 | 118 | 28 | 8 | 60 | 391.043 | 3.947 | 34.781 | 57.969 | 391.050 | 3.950 | 50.400 | 12.600 |
| **2** | 0.590 | 333 | 203 | 32 | 8 | 90 | 391.041 | 3.949 | 18.500 | 55.500 | 391.050 | 3.950 | 51.031 | 13.159 |
| **8** | 0.590 | 465 | 302 | 37 | 9 | 117 | 391.057 | 3.956 | 36.851 | 57.397 | 1739.152 | 87.688 | 44.318 | 8.442 |
| **10** | 0.590 | 460 | 296 | 37 | 10 | 117 | 391.052 | 3.944 | 30.707 | 58.558 | 23.581 | 1.802 | 41.153 | 6.867 |
| **20** | 0.590 | 456 | 293 | 33 | 11 | 119 | 391.054 | 3.950 | 30.707 | 58.558 | 23.581 | 1.802 | 41.153 | 6.867 |
| **21** | 0.590 | 417 | 278 | 30 | 10 | 99 | 391.040 | 3.952 | 30.707 | 58.558 | 23.581 | 1.802 | 41.153 | 6.867 |
| **35** | 0.590 | 380 | 258 | 26 | 8 | 88 | 391.059 | 3.941 | 38.000 | 57.000 | 19.332 | 1.169 | 34.201 | 4.270 |
| **45** | 0.590 | 369 | 253 | 26 | 7 | 83 | 391.057 | 3.952 | 38.000 | 57.000 | 19.332 | 1.169 | 34.201 | 4.270 |
| **150** | 0.590 | 196 | 132 | 15 | 3 | 46 | 391.044 | 3.943 | 49.500 | 49.500 | 1092.725 | 32.635 | 36.964 | 5.184 |
| **200** | 0.590 | 188 | 128 | 15 | 3 | 42 | 391.044 | 3.941 | 49.500 | 49.500 | 1092.725 | 32.635 | 36.964 | 5.184 |
| **400** | 0.590 | 191 | 131 | 15 | 3 | 42 | 391.044 | 3.959 | 55.876 | 40.628 | 1092.725 | 32.635 | 33.481 | 4.054 |
| **500** | 0.590 | 109 | 82 | 13 | 2 | 12 | 391.046 | 3.959 | 58.488 | 31.632 | 543.444 | 7.716 | 33.481 | 4.054 |
| **ALL** | 0.590 | 632 | 436 | 43 | 15 | 138 | 391.051 | 3.956 | 40.874 | 55.755 | 1518.524 | 65.472 | 40.524 | 6.588 |

# Table S3. Characteristics of Included Studies not shown in the main table

| **Authora** | **Publish Year** | **Country** | **Study Design** | **Age** | **Male(%)** | **Smoke(%)** | **Size ≤ 3cm(%)b** | **N0(%)** | **Detection Threshold of VAF** | **Coverage of Sequencingc** |
| --- | --- | --- | --- | --- | --- | --- | --- | --- | --- | --- |
| Zhang(B) | 2023 | CHN | Single center | NA | 28.9 | 26.7 | >90 | 100 | 0.03 | NA |
| Lee | 2022 | KOR | Single center | 67(44-82) | 37 | 34.7 | 80 | 89.1 | 0.05 | min100 |
| Li | 2022 | CHN | Single center | 59 | 24.4 | 34.7 | 90 | 100 | 0.05 | mean500 |
| Qiu(A) | 2022 | CHN | Single center | NA | 26.7 | NA | NA | NA | NA | NA |
| Xu | 2022 | CHN | Single center | NA | 38 | 22 | NA | 80 | NA | NA |
| Zhou | 2022 | CHN | Single center | 48 | 26.3 | NA | 100 | 100 | 0.01 | min20 |
| Belardinilli | 2021 | ITA | Single center | 70 | 60 | NA | 100 | 100 | 0.05 | 500 |
| Bruehl | 2021 | USA | Single center | 67(43-84) | 37.5 | 94 | NA | 87.5 | 0.04 | min100 |
| Ezer | 2021 | CAN | Single center | NA | 31.1 | 82 | NA | 67.3 | NA | NA |
| Goodwin | 2021 | AUS | Single center | 70(50–80) | 47.5 | 75 | NA | NA | 0.05 | NA |
| Hu | 2021 | CHN | Single center | NA | 26.8 | 11.6 | NA | NA | 0.01 | min30 |
| Izumi | 2021 | JPN | Single center | 73(50–83) | 70.6 | 64.71 | NA | NA | 0.03 | min250 |
| Pei | 2021 | CHN | Single center | 60（41–78） | 28.7 | 20 | NA | NA | 0.05 | 10000 |
| Qu | 2021 | CHN | Single center | 61.43（47–69） | 62.5 | 62.5 | 71 | 0 | NA | NA |
| Yang | 2021 | CHN | Single center | 64.13(30-81) | 37.5 | NA | 58 | NA | 0.05 | NA |
| Zhang | 2021 | CHN | Single center | 58(33-75) | 26.19 | 19 | 100 | 96.8 | NA | median1000 |
| Chen | 2020 | CHN | Single center | 58(45-81) | 23.53 | 17.65 | NA | 76.47 | NA | min100 |
| Donfrancesco | 2020 | FRA | Single center | 66 | 70.83 | NA | NA | NA | 0.05 | over 300 |
| Duan | 2020 | CHN | Single center | 60.56 | 43.75 | 31.25 | 94 | 100 | NA | min100 |
| Higuchi | 2020 | JPN | Single center | 70.5(54–85) | 83.78 | 81.08 | NA | 86.49 | 0.01 | 1411(106-5096) |
| Liu(B) | 2020 | CHN | Single center | 57.4 | 33.33 | 60 | 91 | 93.33 | 0.01 | 2000 |
| Pagan | 2020 | USA | Single center | 71(45-91) | 30 | NA | NA | 76.59 | 0.05 | min500 |
| Qiu(B) | 2020 | CHN | Single center | NA | NA | NA | NA | NA | 0.05 | min500 |
| Rodriguez | 2020 | USA | Single center | NA | NA | 83.33 | NA | NA | 0.02 | min100 |
| Wang | 2020 | CHN | Single center | 57.1(34-78)/ 57.8(40-69) | 71.4/81.3 | NA | NA | NA | NA | mean5000 |
| Zheng | 2020 | USA | Single center | 69.61 | 38.89 | 83.33 | NA | 83.3 | NA | NA |
| Chang | 2019 | USA | Single center | 69.42 | 28.33 | 80 | NA | 63.33 | 0.05 | 704(186-1132) |
| Mansuet-Lupo | 2019 | FRA | Single center | 63.5(40–85) | 59 | 59 | 75 | 60 | 0.02 | min300 |
| Vincenten | 2019 | NLD | Single center | NA | NA | NA | NA | NA | NA | NA |
| Roepman | 2018 | NLD | Single center | 63(44-82) | 54 | 84 | NA | NA | 0.05 | over 100 |
| Takahashi | 2018 | JPN | Single center | 67(49‐88) | 56.8 | NA | NA | NA | 0.08 | mean1677 |
| Asmar | 2017 | USA | Single center | NA | NA | NA | NA | NA | NA | NA |
| Goto | 2017 | JPN | Single center | 70.58(54-82) | 83.33 | 83.33 | 75 | 83.33 | 0.01 | 1063(273–2471) |
| Patel | 2017 | USA | Single center | NA | 36.36 | 81.81 | 91 | 81.81 | 0.05 | over 250 |
| Saab | 2017 | USA | Single center | 66(51-78) | 27.78 | NA | 94 | NA | NA | NA |
| Xiao | 2017 | CHN | Single center | NA | NA | NA | NA | NA | NA | NA |
| Liu | 2016 | CHN | Single center | NA | NA | NA | NA | NA | NA | NA |
| Liu(A) | 2016 | CHN | Single center | NA | NA | NA | 73 | 83.33 | >0.05 | mean35/62 |
| Arai | 2012 | JPN | Single center | 65.5 | 50 | 75 | 71 | 100 | NA | NA |
| Takamochi | 2012 | JPN | Single center | 66.17 | 53.33 | 60 | 75 | 80 | NA | NA |
| Girard | 2010 | USA | Single center | 66 | 14.29 | 57.14 | 86 | 57.14 | NA | NA |

a. Author: Some studies include authors with the same surname, differentiated by suffixes.

b. Size (≤ 3cm): Percentages indicate the proportion of cases ≤ 3 cm; other values indicate average diameter. c. Sequencing Coverage: Text explanations include the corresponding coverage value; otherwise, numbers represent "average (range)".

# Table S4. Diagnostic results of included cases under different methods

| **Case ID** | **Clinical** | **HPE** | **Panel** | **Mole1** | **Mole2** | **P value** |
| --- | --- | --- | --- | --- | --- | --- |
| Takahashi1 | MPLC | MPLC | 20 | IPM | MPLC | 1 |
| Takahashi2 | MPLC | IPM | 20 | IPM | IPM | 0 |
| Takahashi3 | MPLC | MPLC | 20 | IN | IN |  |
| Takahashi4 | IPM | MPLC | 20 | IN | IN |  |
| Takahashi5 | MPLC | IN | 20 | MPLC | MPLC | 1 |
| Takahashi6 | MPLC | MPLC | 20 | IPM | IPM | 0.017 |
| Takahashi7 | MPLC | MPLC | 20 | IPM | IPM | 0.025 |
| Takahashi8 | MPLC | MPLC | 20 | IPM | IPM | 0.003 |
| Takahashi9 | IPM | IPM | 20 | IPM | IPM | 0 |
| Takahashi10 | MPLC | MPLC | 20 | IN | IN |  |
| Takahashi11 | MPLC | MPLC | 20 | IN | IN |  |
| Takahashi12 | IPM | MPLC | 20 | IN | IN |  |
| Takahashi13 | MPLC | MPLC | 20 | IN | IN |  |
| Takahashi14 | MPLC | MPLC | 20 | IN | IN |  |
| Takahashi15 | MPLC | IN | 20 | IN | IN |  |
| Takahashi16 | MPLC | MPLC | 20 | MPLC | MPLC | 1 |
| Takahashi17 | MPLC | IPM | 20 | IN | IN |  |
| Takahashi18 | MPLC | IN | 20 | IPM | IPM | 0 |
| Takahashi19 | MPLC | IN | 20 | MPLC | MPLC | 1 |
| Takahashi20 | MPLC | MPLC | 20 | IPM | IPM | 0 |
| Takahashi21 | MPLC | IN | 20 | IPM | IPM | 0 |
| Takahashi22 | MPLC | IN | 20 | IN | IN |  |
| Takahashi23 | MPLC | MPLC | 20 | IN | IN |  |
| Takahashi24 | MPLC | IPM | 20 | IPM | IPM | 0 |
| Takahashi25 | MPLC | IPM | 20 | IPM | IPM | 0 |
| Takahashi26 | MPLC | IPM | 20 | IPM | IPM | 0 |
| Takahashi27 | MPLC | MPLC | 20 | IPM | IPM | 0 |
| Takahashi28 | MPLC | MPLC | 20 | IPM | IPM | 0 |
| Takahashi29 | MPLC | MPLC | 20 | IPM | IPM | 0.001 |
| Takahashi30 | MPLC | IN | 20 | IN | IN |  |
| Takahashi31 | MPLC | IN | 20 | IN | IN |  |
| Takahashi32 | MPLC | IN | 20 | IPM | IPM | 0 |
| Takahashi33 | MPLC | IPM | 20 | IPM | IPM | 0 |
| Takahashi34 | MPLC | IN | 20 | IN | IN |  |
| Takahashi35 | MPLC | IN | 20 | IPM | IPM | 0.033 |
| Takahashi36 | MPLC | IN | 20 | IPM | IPM | 0 |
| Takahashi37 | MPLC | IN | 20 | IPM | IPM | 0.002 |
| Chang1 |  | MPLC | 400 | MPLC | MPLC | 1 |
| Chang2 |  | IPM | 400 | MPLC | MPLC | 1 |
| Chang3 |  | MPLC | 400 | IPM | IPM | 0 |
| Chang4 |  | MPLC | 400 | IN | IPM | 0.031 |
| Chang5 |  | IPM | 400 | IPM | IPM | 0 |
| Chang6 |  | IPM | 400 | IPM | IPM | 0 |
| Chang7 |  | MPLC | 400 | MPLC | MPLC | 1 |
| Chang8 |  | MPLC | 400 | IPM | IPM | 0 |
| Chang9 |  | MPLC | 400 | MPLC | MPLC | 1 |
| Chang10 |  | MPLC | 400 | MPLC | MPLC | 1 |
| Chang11 |  | MPLC | 400 | MPLC | MPLC | 1 |
| Chang12 |  | MPLC | 400 | MPLC | MPLC | 1 |
| Chang13 |  | MPLC | 400 | MPLC | MPLC | 1 |
| Chang14 |  | MPLC | 400 | MPLC | MPLC | 1 |
| Chang15 |  | MPLC | 400 | MPLC | MPLC | 1 |
| Chang16 |  | MPLC | 400 | IN | IN |  |
| Chang17 |  | MPLC | 400 | MPLC | MPLC | 1 |
| Chang18 |  | MPLC | 400 | IPM | IPM | 0 |
| Chang19 |  | MPLC | 400 | IN | IPM | 0.018 |
| Chang20 |  | IPM | 400 | MPLC | MPLC | 1 |
| Chang21 |  | IPM | 400 | IPM | IPM | 0 |
| Chang22 |  | MPLC | 400 | IN | IPM | 0.086 |
| Chang23 |  | IPM | 400 | IPM | IPM | 0 |
| Chang24 |  | MPLC | 400 | MPLC | MPLC | 1 |
| Chang25 |  | MPLC | 400 | MPLC | MPLC | 1 |
| Chang26 |  | MPLC | 400 | MPLC | MPLC | 1 |
| Chang27 |  | MPLC | 400 | IPM | IPM | 0 |
| Chang28 |  | MPLC | 400 | MPLC | MPLC | 1 |
| Chang29 |  | IPM | 400 | IN | IPM | 0.004 |
| Chang30 |  | MPLC | 400 | MPLC | MPLC | 1 |
| Chang31 |  | IPM | 400 | IPM | IPM | 0 |
| Chang32 |  | IPM | 400 | IPM | IPM | 0 |
| Chang33 |  | MPLC | 400 | MPLC | MPLC | 1 |
| Chang34 |  | MPLC | 400 | MPLC | MPLC | 1 |
| Chang35 |  | MPLC | 400 | MPLC | MPLC | 1 |
| Chang36 |  | MPLC | 400 | IN | IPM | 0.003 |
| Chang37 |  | MPLC | 400 | MPLC | MPLC | 1 |
| Chang38 |  | IPM | 400 | MPLC | MPLC | 1 |
| Chang39 |  | IPM | 400 | IN | IPM | 0.018 |
| Chang40 |  | MPLC | 400 | MPLC | MPLC | 1 |
| Chang41 |  | IPM | 400 | IPM | IPM | 0 |
| Chang42 |  | MPLC | 400 | IN | IPM | 0.071 |
| Chang43 |  | MPLC | 400 | IN | IN |  |
| Chang44 |  | MPLC | 400 | MPLC | MPLC | 1 |
| Chang45 |  | IPM | 400 | IPM | IPM | 0 |
| Chang46 |  | MPLC | 400 | MPLC | MPLC | 1 |
| Chang47 |  | IPM | 400 | IPM | IPM | 0 |
| Chang48 |  | MPLC | 400 | MPLC | MPLC | 1 |
| Chang49 |  | MPLC | 400 | IPM | IPM | 0 |
| Chang50 |  | MPLC | 400 | IPM | IPM | 0 |
| Chang51 |  | MPLC | 400 | IPM | IPM | 0 |
| Chang52 |  | MPLC | 400 | MPLC | MPLC | 1 |
| Chang53 |  | MPLC | 400 | MPLC | MPLC | 1 |
| Chang54 |  | MPLC | 400 | MPLC | MPLC | 1 |
| Chang55 |  | MPLC | 400 | MPLC | MPLC | 1 |
| Chang56 |  | MPLC | 400 | MPLC | MPLC | 1 |
| Chang57 |  | MPLC | 400 | MPLC | MPLC | 1 |
| Chang58 |  | MPLC | 400 | MPLC | MPLC | 1 |
| Chang59 |  | MPLC | 400 | MPLC | MPLC | 1 |
| Chang60 |  | IPM | 400 | MPLC | MPLC | 1 |
| Zhangb1 |  | MPLC | 425 | Unknown | Unknown |  |
| Zhangb2 |  | MPLC | 425 | Unknown | Unknown |  |
| Zhangb3 |  | MPLC | 425 | Unknown | Unknown |  |
| Zhangb4 |  | MPLC | 425 | Unknown | Unknown |  |
| Zhangb5 |  | MPLC | 425 | Unknown | Unknown |  |
| Zhangb6 |  | MPLC | 425 | Unknown | Unknown |  |
| Zhangb7 |  | MPLC | 425 | Unknown | Unknown |  |
| Zhangb8 |  | IPM | 425 | Unknown | Unknown |  |
| Zhangb9 |  | MPLC | 425 | Unknown | Unknown |  |
| Zhangb10 |  | MPLC | 425 | Unknown | Unknown |  |
| Zhangb11 |  | MPLC | 425 | Unknown | Unknown |  |
| Zhangb12 |  | MPLC | 425 | Unknown | Unknown |  |
| Zhangb13 |  | MPLC | 425 | Unknown | Unknown |  |
| Zhangb14 |  | MPLC | 425 | Unknown | Unknown |  |
| Zhangb15 |  | MPLC | 425 | Unknown | Unknown |  |
| Zhangb16 |  | MPLC | 425 | Unknown | Unknown |  |
| Zhangb17 |  | MPLC | 425 | Unknown | Unknown |  |
| Zhangb18 |  | MPLC | 425 | Unknown | Unknown |  |
| Zhangb19 |  | MPLC | 425 | Unknown | Unknown |  |
| Zhangb20 |  | MPLC | 425 | Unknown | Unknown |  |
| Zhangb21 |  | MPLC | 425 | Unknown | Unknown |  |
| Zhangb22 |  | MPLC | 425 | Unknown | Unknown |  |
| Zhangb23 |  | MPLC | 425 | Unknown | Unknown |  |
| Zhangb24 |  | MPLC | 425 | Unknown | Unknown |  |
| Zhangb25 |  | MPLC | 425 | Unknown | Unknown |  |
| Zhangb26 |  | MPLC | 425 | Unknown | Unknown |  |
| Zhangb27 |  | MPLC | 425 | Unknown | Unknown |  |
| Zhangb28 |  | MPLC | 425 | Unknown | Unknown |  |
| Zhangb29 |  | MPLC | 425 | Unknown | Unknown |  |
| Zhangb30 |  | MPLC | 425 | Unknown | Unknown |  |
| Zhangb31 |  | MPLC | 425 | Unknown | Unknown |  |
| Zhangb32 |  | MPLC | 425 | Unknown | Unknown |  |
| Zhangb33 |  | MPLC | 425 | Unknown | Unknown |  |
| Zhangb34 |  | MPLC | 425 | Unknown | Unknown |  |
| Zhangb35 |  | MPLC | 425 | Unknown | Unknown |  |
| Zhangb36 |  | MPLC | 425 | Unknown | Unknown |  |
| Zhangb37 |  | MPLC | 425 | Unknown | Unknown |  |
| Zhangb38 |  | MPLC | 425 | Unknown | Unknown |  |
| Zhangb39 |  | MPLC | 425 | Unknown | Unknown |  |
| Zhangb40 |  | MPLC | 425 | Unknown | Unknown |  |
| Zhangb41 |  | MPLC | 425 | Unknown | Unknown |  |
| Zhangb42 |  | IPM | 425 | Unknown | Unknown |  |
| Zhangb43 |  | MPLC | 425 | Unknown | Unknown |  |
| Zhangb44 |  | MPLC | 425 | Unknown | Unknown |  |
| Zhangb45 |  | IPM | 425 | Unknown | Unknown |  |
| Bruehl1 | IPM | IPM | 35 | IN | IN |  |
| Bruehl2 | IPM | IPM | 35 | IN | IPM | 0.068 |
| Bruehl3 | MPLC | MPLC | 35 | MPLC | MPLC | 1 |
| Bruehl4 | MPLC | MPLC | 35 | IN | IPM | 0.068 |
| Bruehl5 | IPM | IPM | 35 | MPLC | MPLC | 1 |
| Bruehl6 | MPLC | MPLC | 35 | IN | IN |  |
| Bruehl7 | IPM | IPM | 35 | IPM | IPM | 0 |
| Bruehl8 | IPM | IPM | 35 | IPM | IPM | 0.017 |
| Bruehl9 | MPLC | MPLC | 35 | IN | IN |  |
| Bruehl10 | IPM | MPLC | 35 | IN | IPM | 0.033 |
| Bruehl11 | IPM | IPM | 35 | IPM | IPM | 0 |
| Bruehl12 | IPM | IPM | 35 | IPM | IPM | 0 |
| Bruehl13 | IPM | IPM | 35 | IPM | IPM | 0.001 |
| Bruehl14 | IPM | IPM | 35 | IN | IN |  |
| Bruehl15 | IPM | IPM | 35 | IN | IN |  |
| Bruehl16 | IPM | IPM | 35 | IPM | IPM | 0.003 |
| Bruehl17 | MPLC | IPM | 35 | MPLC | MPLC | 1 |
| Bruehl18 | MPLC | MPLC | 35 | MPLC | MPLC | 1 |
| Bruehl19 | MPLC | MPLC | 35 | IN | IN |  |
| Bruehl20 | IPM | IPM | 35 | IN | IN |  |
| Bruehl21 | MPLC | MPLC | 35 | IN | IN |  |
| Bruehl22 | MPLC | MPLC | 35 | MPLC | MPLC | 1 |
| Bruehl23 | MPLC | MPLC | 35 | MPLC | MPLC | 1 |
| Bruehl24 | MPLC | IPM | 35 | MPLC | MPLC | 1 |
| Bruehl25 | MPLC | MPLC | 35 | MPLC | MPLC | 1 |
| Bruehl26 | MPLC | MPLC | 35 | IN | IN |  |
| Bruehl27 | MPLC | MPLC | 35 | MPLC | MPLC | 1 |
| Bruehl28 | MPLC | MPLC | 35 | IN | IN |  |
| Bruehl29 | IPM | MPLC | 35 | IN | IN |  |
| Bruehl30 | IPM | MPLC | 35 | IN | IN |  |
| Bruehl31 | MPLC | MPLC | 35 | IN | IN |  |
| Bruehl32 | IPM | IPM | 35 | IN | IPM | 0.068 |
| Lee1 | MPLC | MPLC | 1 | IN | IPM | 0 |
| Lee2 | MPLC | MPLC | 1 | IN | IPM | 0 |
| Lee3 | MPLC | MPLC | 1 | IN | IPM | 0 |
| Lee4 | MPLC | MPLC | 1 | IN | IPM | 0 |
| Lee5 | MPLC | MPLC | 1 | IN | IPM | 0 |
| Lee6 | MPLC | IPM | 1 | IN | IPM | 0.017 |
| Lee7 | MPLC | IPM | 1 | IN | IPM | 0.017 |
| Lee8 | MPLC | MPLC | 1 | IN | IPM | 0.017 |
| Lee9 | MPLC | IPM | 1 | IN | IPM | 0.017 |
| Lee10 | MPLC | IPM | 1 | IN | IPM | 0.017 |
| Lee11 | MPLC | IPM | 1 | IN | IPM | 0.017 |
| Lee12 | MPLC | IPM | 1 | IN | IPM | 0.017 |
| Lee13 | MPLC | IPM | 1 | IN | IPM | 0.017 |
| Lee14 | MPLC | IPM | 1 | IN | IPM | 0.017 |
| Lee15 | MPLC | MPLC | 1 | IN | IPM | 0.017 |
| Lee16 | MPLC | IPM | 1 | IN | IPM | 0.017 |
| Lee17 | MPLC | IPM | 1 | IN | IPM | 0.017 |
| Lee18 | MPLC | MPLC | 1 | IN | IPM | 0.017 |
| Lee19 | MPLC | MPLC | 1 | IN | IPM | 0.017 |
| Lee20 | MPLC | MPLC | 1 | IN | IPM | 0.017 |
| Lee21 | MPLC | IPM | 1 | IN | IN |  |
| Lee22 | MPLC | IPM | 1 | IN | IN |  |
| Lee23 | MPLC | MPLC | 1 | IN | IN |  |
| Lee24 | MPLC | MPLC | 1 | IN | IN |  |
| Lee25 | MPLC | MPLC | 1 | IN | IN |  |
| Lee26 | MPLC | IPM | 1 | IN | IN |  |
| Lee27 | MPLC | IPM | 1 | IN | IN |  |
| Lee28 | MPLC | IPM | 1 | IN | IN |  |
| Lee29 | MPLC | MPLC | 1 | IN | IN |  |
| Lee30 | MPLC | IPM | 1 | IN | IN |  |
| Lee31 | MPLC | IPM | 1 | IN | IN |  |
| Lee32 | MPLC | IPM | 1 | IN | IN |  |
| Lee33 | MPLC | IPM | 1 | IN | IN |  |
| Lee34 | MPLC | IPM | 1 | IN | IN |  |
| Lee35 | MPLC | IPM | 1 | IN | IN |  |
| Lee36 | MPLC | IPM | 1 | IN | IN |  |
| Lee37 | MPLC | MPLC | 1 | IN | IN |  |
| Lee38 | MPLC | MPLC | 1 | IN | IN |  |
| Lee39 | MPLC | MPLC | 1 | IN | IN |  |
| Lee40 | MPLC | IPM | 1 | IN | IN |  |
| Lee41 | MPLC | IPM | 1 | IN | IN |  |
| Lee42 | MPLC | IPM | 1 | IN | IN |  |
| Lee43 | MPLC | IPM | 1 | IN | IN |  |
| Lee44 | MPLC | IPM | 1 | IN | IN |  |
| Lee45 | MPLC | MPLC | 1 | IN | IN |  |
| Lee46 | MPLC | MPLC | 1 | IN | IN |  |
| Lee47 | MPLC | MPLC | 1 | IN | IN |  |
| Lee48 | MPLC | MPLC | 1 | IN | IN |  |
| Lee49 | MPLC | IPM | 1 | IN | MPLC | 1 |
| Lee50 | MPLC | IPM | 1 | IN | MPLC | 1 |
| Lee51 | MPLC | IPM | 1 | IN | MPLC | 1 |
| Lee52 | MPLC | MPLC | 1 | IN | MPLC | 1 |
| Lee53 | MPLC | IPM | 1 | IN | MPLC | 1 |
| Lee54 | MPLC | IPM | 1 | IN | MPLC | 1 |
| Lee55 | MPLC | IPM | 1 | IN | IN |  |
| Lee56 | MPLC | MPLC | 1 | IN | IN |  |
| Lee57 | MPLC | MPLC | 1 | IN | IN |  |
| Lee58 | MPLC | IPM | 1 | IN | IN |  |
| Lee59 | MPLC | IPM | 1 | IN | IN |  |
| Lee60 | MPLC | IPM | 1 | IN | IN |  |
| Lee61 | MPLC | MPLC | 1 | IN | IN |  |
| Lee62 | MPLC | MPLC | 1 | IN | IN |  |
| Lee63 | MPLC | MPLC | 1 | IN | IN |  |
| Lee64 | MPLC | MPLC | 1 | IN | IN |  |
| Lee65 | MPLC | IPM | 1 | IN | IN |  |
| Lee66 | MPLC | IPM | 1 | IN | IN |  |
| Lee67 | MPLC | IPM | 1 | IN | IN |  |
| Lee68 | MPLC | MPLC | 1 | IN | IN |  |
| Lee69 | MPLC | MPLC | 1 | IN | IN |  |
| Lee70 | MPLC | IPM | 1 | IN | IN |  |
| Lee71 | MPLC | IPM | 1 | IN | IN |  |
| Lee72 | MPLC | IPM | 1 | MPLC | MPLC | 1 |
| Lee73 | MPLC | IPM | 1 | MPLC | MPLC | 1 |
| Lee74 | MPLC | IPM | 1 | MPLC | MPLC | 1 |
| Lee75 | MPLC | IPM | 1 | MPLC | MPLC | 1 |
| Lee76 | MPLC | IPM | 1 | MPLC | MPLC | 1 |
| Lee77 | MPLC | IPM | 1 | IN | IN |  |
| Lee78 | MPLC | MPLC | 1 | MPLC | MPLC | 1 |
| Lee79 | MPLC | IPM | 1 | IN | IN |  |
| Lee80 | MPLC | MPLC | 1 | IN | IN |  |
| Lee81 | MPLC | MPLC | 1 | IN | IN |  |
| Lee82 | MPLC | IPM | 1 | IN | IN |  |
| Lee83 | MPLC | MPLC | 1 | IN | IN |  |
| Lee84 | MPLC | IPM | 1 | IN | IN |  |
| Lee85 | MPLC | IPM | 1 | IN | IN |  |
| Lee86 | MPLC | MPLC | 1 | IN | IN |  |
| Lee87 | MPLC | IPM | 1 | MPLC | MPLC | 1 |
| Lee88 | MPLC | IPM | 1 | IN | IN |  |
| Lee89 | MPLC | MPLC | 1 | IN | IN |  |
| Lee90 | MPLC | IPM | 1 | IN | IN |  |
| Lee91 | MPLC | MPLC | 1 | IN | IN |  |
| Lee92 | MPLC | IPM | 1 | IN | IN |  |
| Lee93 | MPLC | MPLC | 1 | IN | IN |  |
| Lee94 | MPLC | MPLC | 1 | IN | IN |  |
| Lee95 | MPLC | MPLC | 1 | IN | IN |  |
| Lee96 | MPLC | MPLC | 1 | IN | IN |  |
| Lee97 | MPLC | MPLC | 1 | IN | IN |  |
| Lee98 | MPLC | IPM | 1 | IN | IN |  |
| Lee99 | MPLC | MPLC | 1 | IN | IN |  |
| Lee100 | MPLC | IPM | 1 | IPM | MPLC | 1 |
| Lee101 | MPLC | IPM | 1 | IPM | IPM | 0.017 |
| Li1 | MPLC |  | 500 | MPLC | MPLC | 1 |
| Li2 | MPLC |  | 500 | MPLC | MPLC | 1 |
| Li3 | MPLC |  | 500 | MPLC | MPLC | 1 |
| Li4 | MPLC |  | 500 | MPLC | MPLC | 1 |
| Li5 | MPLC |  | 500 | MPLC | MPLC | 1 |
| Li6 | MPLC |  | 500 | MPLC | IPM | 0.001 |
| Li7 | MPLC |  | 500 | MPLC | MPLC | 1 |
| Li8 | MPLC |  | 500 | IN | IN |  |
| Li9 | MPLC |  | 500 | MPLC | IPM | 0.001 |
| Li10 | MPLC |  | 500 | IN | IPM | 0 |
| Li11 | MPLC |  | 500 | IN | IPM | 0 |
| Li12 | MPLC |  | 500 | MPLC | MPLC | 1 |
| Li13 | MPLC |  | 500 | IN | IN |  |
| Li14 | MPLC |  | 500 | IN | IPM | 0.017 |
| Li15 | MPLC |  | 500 | IN | IN |  |
| Li16 | MPLC |  | 500 | MPLC | MPLC | 1 |
| Li17 | MPLC |  | 500 | MPLC | MPLC | 1 |
| Li18 | MPLC |  | 500 | MPLC | MPLC | 1 |
| Li19 | MPLC |  | 500 | MPLC | MPLC | 1 |
| Li20 | MPLC |  | 500 | MPLC | MPLC | 1 |
| Li21 | MPLC |  | 500 | IN | IN |  |
| Li22 | MPLC |  | 500 | IN | IPM | 0.017 |
| Li23 | MPLC |  | 500 | IN | IN |  |
| Li24 | MPLC |  | 500 | MPLC | MPLC | 1 |
| Li25 | MPLC |  | 500 | MPLC | MPLC | 1 |
| Li26 | MPLC |  | 500 | IN | IN |  |
| Li27 | MPLC |  | 500 | MPLC | MPLC | 1 |
| Li28 | MPLC |  | 500 | MPLC | IPM | 0 |
| Li29 | MPLC |  | 500 | MPLC | MPLC | 1 |
| Li30 | MPLC |  | 500 | IN | IPM | 0.017 |
| Li31 | MPLC |  | 500 | MPLC | IPM | 0.017 |
| Li32 | MPLC |  | 500 | IN | IN |  |
| Li33 | MPLC |  | 500 | IN | IN |  |
| Li34 | MPLC |  | 500 | MPLC | MPLC | 1 |
| Li35 | MPLC |  | 500 | MPLC | MPLC | 1 |
| Li36 | MPLC |  | 500 | MPLC | IPM | 0 |
| Li37 | MPLC |  | 500 | MPLC | MPLC | 1 |
| Li38 | MPLC |  | 500 | MPLC | MPLC | 1 |
| Li39 | MPLC |  | 500 | MPLC | MPLC | 1 |
| Li40 | MPLC |  | 500 | MPLC | IPM | 0.033 |
| Li41 | MPLC |  | 500 | IN | IN |  |
| QiuA1 | MPLC | IPM | 20 | IN | IN |  |
| QiuA2 | MPLC | MPLC | 20 | MPLC | MPLC | 1 |
| QiuA3 | MPLC | MPLC | 20 | MPLC | MPLC | 1 |
| QiuA4 | MPLC | MPLC | 20 | IN | IN |  |
| QiuA5 | MPLC | MPLC | 20 | IPM | IPM | 0 |
| QiuA6 | MPLC | MPLC | 20 | MPLC | IPM | 0 |
| QiuA7 | MPLC | MPLC | 20 | IN | IN |  |
| QiuA8 | MPLC | MPLC | 20 | IN | IN |  |
| QiuA9 | MPLC | MPLC | 20 | IN | IN |  |
| QiuA10 | MPLC | IPM | 20 | MPLC | MPLC | 1 |
| QiuA11 | MPLC | MPLC | 20 | IN | IPM | 0.017 |
| QiuA12 | MPLC | MPLC | 20 | MPLC | MPLC | 1 |
| QiuA13 | MPLC | MPLC | 20 | MPLC | MPLC | 1 |
| QiuA14 | MPLC | MPLC | 20 | IN | IN |  |
| QiuA15 | MPLC | MPLC | 20 | MPLC | IPM | 0.017 |
| QiuA16 | MPLC | MPLC | 20 | IN | IN |  |
| QiuA17 | MPLC | MPLC | 20 | MPLC | MPLC | 1 |
| QiuA18 | MPLC | MPLC | 20 | MPLC | IPM | 0 |
| QiuA19 | MPLC | MPLC | 20 | IN | IN |  |
| QiuA20 | MPLC | MPLC | 20 | IN | IN |  |
| QiuA21 | MPLC | MPLC | 20 | MPLC | MPLC | 1 |
| QiuA22 | MPLC | MPLC | 20 | MPLC | MPLC | 1 |
| QiuA23 | MPLC | MPLC | 20 | IN | IN |  |
| QiuA24 | MPLC | IPM | 20 | MPLC | MPLC | 1 |
| QiuA25 | MPLC | MPLC | 20 | MPLC | MPLC | 1 |
| QiuA26 | IPM | IPM | 20 | IPM | IPM | 0 |
| QiuA27 | MPLC | MPLC | 20 | IN | IN |  |
| QiuA28 | MPLC | IPM | 20 | MPLC | MPLC | 1 |
| QiuA29 | MPLC | MPLC | 20 | MPLC | MPLC | 1 |
| QiuA30 | MPLC | MPLC | 20 | IN | IN |  |
| Xu1 | MPLC |  | 1 | MPLC | IPM | 0.017 |
| Xu2 | MPLC |  | 1 | MPLC | IPM | 0 |
| Xu3 | IPM |  | 1 | IN | IN |  |
| Xu4 | IPM |  | 1 | IPM | IPM | 0 |
| Xu5 | IPM |  | 1 | IPM | IPM | 0 |
| Xu6 | MPLC |  | 1 | IN | IN |  |
| Xu7 | MPLC |  | 1 | IN | IN |  |
| Xu8 | MPLC |  | 1 | IN | IN |  |
| Xu9 | IPM |  | 1 | IN | IN |  |
| Xu10 | IPM |  | 1 | IN | IN |  |
| Xu11 | IPM |  | 1 | IPM | IPM | 0.017 |
| Xu12 | MPLC |  | 1 | IN | IN |  |
| Xu13 | MPLC |  | 1 | IN | IN |  |
| Xu14 | MPLC |  | 1 | MPLC | IPM | 0 |
| Xu15 | MPLC |  | 1 | IN | IN |  |
| Xu16 | IPM |  | 1 | IN | IN |  |
| Xu17 | MPLC |  | 1 | IPM | MPLC | 1 |
| Xu18 | MPLC |  | 1 | IN | IN |  |
| Xu19 | IPM |  | 1 | IPM | IPM | 0 |
| Xu20 | MPLC |  | 1 | IN | IN |  |
| Xu21 | IPM |  | 1 | IN | IN |  |
| Xu22 | MPLC |  | 1 | IN | IN |  |
| Xu23 | MPLC |  | 1 | MPLC | IPM | 0 |
| Xu24 | MPLC |  | 1 | IN | IN |  |
| Xu25 | MPLC |  | 1 | IN | IN |  |
| Xu26 | IPM |  | 1 | IN | IN |  |
| Xu27 | MPLC |  | 1 | IN | IN |  |
| Xu28 | MPLC |  | 1 | MPLC | IPM | 0.017 |
| Xu29 | MPLC |  | 1 | IN | IN |  |
| Xu30 | MPLC |  | 1 | IN | IN |  |
| Xu31 | IPM |  | 1 | IN | IN |  |
| Xu32 | MPLC |  | 1 | IPM | MPLC | 1 |
| Xu33 | MPLC |  | 1 | IN | IN |  |
| Xu34 | MPLC |  | 1 | IN | IN |  |
| Xu35 | MPLC |  | 1 | IN | IN |  |
| Xu36 | IPM |  | 1 | IN | IN |  |
| Xu37 | IPM |  | 1 | IN | IN |  |
| Xu38 | MPLC |  | 1 | IN | IN |  |
| Xu39 | IPM |  | 1 | IN | IN |  |
| Xu40 | MPLC |  | 1 | IN | IN |  |
| Xu41 | MPLC |  | 1 | IN | IN |  |
| Xu42 | MPLC |  | 1 | IPM | MPLC | 1 |
| Xu43 | MPLC |  | 1 | IN | IN |  |
| Xu44 | IPM |  | 1 | IN | IN |  |
| Xu45 | MPLC |  | 1 | IN | IN |  |
| Xu46 | MPLC |  | 1 | IN | IN |  |
| Xu47 | MPLC |  | 1 | IPM | MPLC | 1 |
| Xu48 | MPLC |  | 1 | IN | IN |  |
| Xu49 | IPM |  | 1 | IPM | IPM | 0.017 |
| Xu50 | MPLC |  | 1 | IN | IN |  |
| Zhou1 | MPLC |  | 10000 | MPLC | Unknown |  |
| Zhou2 | MPLC |  | 10000 | MPLC | Unknown |  |
| Zhou3 | MPLC |  | 10000 | MPLC | Unknown |  |
| Zhou4 | MPLC |  | 10000 | IPM | Unknown |  |
| Zhou5 | MPLC |  | 10000 | MPLC | Unknown |  |
| Zhou6 | MPLC |  | 10000 | IPM | Unknown |  |
| Zhou7 | MPLC |  | 10000 | MPLC | Unknown |  |
| Zhou8 | MPLC |  | 10000 | MPLC | Unknown |  |
| Zhou9 | MPLC |  | 10000 | MPLC | Unknown |  |
| Zhou10 | MPLC |  | 10000 | MPLC | Unknown |  |
| Zhou11 | MPLC |  | 10000 | IPM | Unknown |  |
| Zhou12 | MPLC |  | 10000 | MPLC | Unknown |  |
| Zhou13 | MPLC |  | 10000 | MPLC | Unknown |  |
| Zhou14 | MPLC |  | 10000 | MPLC | Unknown |  |
| Zhou15 | MPLC |  | 10000 | IPM | Unknown |  |
| Zhou16 | MPLC |  | 10000 | IPM | Unknown |  |
| Zhou17 | MPLC |  | 10000 | MPLC | Unknown |  |
| Zhou18 | MPLC |  | 10000 | MPLC | Unknown |  |
| Zhou19 | MPLC |  | 10000 | MPLC | Unknown |  |
| Belardinilli1 | MPLC | MPLC | 22 | MPLC | IPM | 0.079 |
| Belardinilli2 | MPLC | MPLC | 22 | MPLC | IN |  |
| Belardinilli3 | MPLC | MPLC | 22 | MPLC | IN |  |
| Belardinilli4 | MPLC | MPLC | 22 | MPLC | MPLC | 1 |
| Belardinilli5 | MPLC | MPLC | 22 | MPLC | MPLC | 1 |
| Belardinilli6 | MPLC | MPLC | 22 | MPLC | IPM | 0.049 |
| Belardinilli7 | IPM | IPM | 22 | MPLC | MPLC | 1 |
| Belardinilli8 | MPLC | MPLC | 22 | MPLC | MPLC | 1 |
| Belardinilli9 | MPLC | MPLC | 22 | MPLC | MPLC | 1 |
| Belardinilli10 | MPLC | MPLC | 22 | MPLC | MPLC | 1 |
| Ezer1 |  | MPLC | 52 | MPLC | MPLC | 1 |
| Ezer2 |  | MPLC | 52 | IN | MPLC | 1 |
| Ezer3 |  | MPLC | 52 | MPLC | IN |  |
| Ezer4 |  | MPLC | 52 | MPLC | IN |  |
| Ezer5 |  | MPLC | 52 | MPLC | MPLC | 1 |
| Ezer6 |  | MPLC | 52 | IN | MPLC | 1 |
| Ezer7 |  | MPLC | 52 | MPLC | IN |  |
| Ezer8 |  | MPLC | 52 | MPLC | MPLC | 1 |
| Ezer9 |  | MPLC | 52 | MPLC | IN |  |
| Ezer10 |  | MPLC | 52 | MPLC | IN |  |
| Ezer11 |  | MPLC | 52 | MPLC | IN |  |
| Ezer12 |  | MPLC | 52 | MPLC | MPLC | 1 |
| Ezer13 |  | MPLC | 52 | MPLC | IN |  |
| Ezer14 |  | MPLC | 52 | MPLC | IN |  |
| Ezer15 |  | MPLC | 52 | MPLC | MPLC | 1 |
| Ezer16 |  | MPLC | 52 | MPLC | MPLC | 1 |
| Ezer17 |  | MPLC | 52 | MPLC | MPLC | 1 |
| Ezer18 |  | MPLC | 52 | IN | IN |  |
| Ezer19 |  | MPLC | 52 | MPLC | MPLC | 1 |
| Ezer20 |  | MPLC | 52 | MPLC | MPLC | 1 |
| Ezer21 |  | MPLC | 52 | IN | IN |  |
| Ezer22 |  | MPLC | 52 | IN | IPM | 0.068 |
| Ezer23 |  | MPLC | 52 | IN | IPM | 0.068 |
| Ezer24 |  | MPLC | 52 | MPLC | MPLC | 1 |
| Ezer25 |  | MPLC | 52 | IN | IN |  |
| Ezer26 |  | MPLC | 52 | MPLC | MPLC | 1 |
| Ezer27 |  | MPLC | 52 | MPLC | MPLC | 1 |
| Ezer28 |  | MPLC | 52 | IN | IPM | 0.004 |
| Ezer29 |  | IN | 52 | IPM | IPM | 0 |
| Ezer30 |  | IPM | 52 | IN | IN |  |
| Ezer31 |  | IPM | 52 | IN | IN |  |
| Ezer32 |  | IPM | 52 | IN | IPM | 0.077 |
| Ezer33 |  | IPM | 52 | IN | IPM | 0.001 |
| Ezer34 |  | MPLC | 52 | MPLC | MPLC | 1 |
| Ezer35 |  | IPM | 52 | IPM | IPM | 0 |
| Ezer36 |  | MPLC | 52 | IN | IN |  |
| Ezer37 |  | MPLC | 52 | IN | IN |  |
| Ezer38 |  | MPLC | 52 | MPLC | MPLC | 1 |
| Ezer39 |  | IPM | 52 | IN | IPM | 0.014 |
| Ezer40 |  | MPLC | 52 | MPLC | MPLC | 1 |
| Ezer41 |  | MPLC | 52 | IN | IN |  |
| Ezer42 |  | IPM | 52 | IN | IN |  |
| Ezer43 |  | MPLC | 52 | MPLC | MPLC | 1 |
| Ezer44 |  | MPLC | 52 | IN | IN |  |
| Ezer45 |  | IN | 52 | MPLC | MPLC | 1 |
| Ezer46 |  | IN | 52 | MPLC | MPLC | 1 |
| Ezer47 |  | MPLC | 52 | IN | IPM | 0.068 |
| Ezer48 |  | MPLC | 52 | MPLC | MPLC | 1 |
| Ezer49 |  | MPLC | 52 | IN | IN |  |
| Ezer50 |  | IPM | 52 | IPM | IPM | 0 |
| Ezer51 |  | IN | 52 | MPLC | MPLC | 1 |
| Ezer52 |  | MPLC | 52 | IPM | IPM | 0 |
| Ezer53 |  | MPLC | 52 | MPLC | MPLC | 1 |
| Ezer54 |  | MPLC | 52 | MPLC | MPLC | 1 |
| Ezer55 |  | IPM | 52 | MPLC | MPLC | 1 |
| Ezer56 |  | MPLC | 52 | MPLC | MPLC | 1 |
| Ezer57 |  | MPLC | 52 | IN | IN |  |
| Ezer58 |  | IPM | 52 | IN | IN |  |
| Ezer59 |  | MPLC | 52 | MPLC | MPLC | 1 |
| Ezer60 |  | MPLC | 52 | IN | IN |  |
| Ezer61 |  | IN | 52 | MPLC | MPLC | 1 |
| Goodwin1 | MPLC |  | 50 | MPLC | MPLC | 1 |
| Goodwin2 | MPLC |  | 50 | IPM | IPM | 0.017 |
| Goodwin3 | MPLC |  | 50 | MPLC | MPLC | 1 |
| Goodwin4 | MPLC |  | 50 | MPLC | IPM | 0 |
| Goodwin5 | MPLC |  | 50 | MPLC | MPLC | 1 |
| Goodwin6 | MPLC |  | 50 | MPLC | MPLC | 1 |
| Goodwin7 | MPLC |  | 50 | MPLC | MPLC | 1 |
| Goodwin8 | MPLC |  | 50 | IPM | IPM | 0.004 |
| Goodwin9 | MPLC |  | 50 | MPLC | MPLC | 1 |
| Goodwin10 | MPLC |  | 50 | IPM | IPM | 0.005 |
| Goodwin11 | MPLC |  | 50 | MPLC | MPLC | 1 |
| Goodwin12 | MPLC |  | 50 | MPLC | MPLC | 1 |
| Goodwin13 | MPLC |  | 50 | MPLC | IPM | 0.002 |
| Goodwin14 | MPLC |  | 50 | MPLC | MPLC | 1 |
| Goodwin15 | MPLC |  | 50 | IPM | IPM | 0 |
| Goodwin16 | MPLC |  | 50 | MPLC | MPLC | 1 |
| Goodwin17 | MPLC |  | 50 | IN | IN |  |
| Goodwin18 | MPLC |  | 50 | MPLC | MPLC | 1 |
| Goodwin19 | MPLC |  | 50 | MPLC | MPLC | 1 |
| Goodwin20 | MPLC |  | 50 | MPLC | MPLC | 1 |
| Goodwin21 | MPLC |  | 50 | MPLC | MPLC | 1 |
| Goodwin22 | MPLC |  | 50 | MPLC | MPLC | 1 |
| Goodwin23 | MPLC |  | 50 | MPLC | MPLC | 1 |
| Goodwin24 | MPLC |  | 50 | MPLC | MPLC | 1 |
| Goodwin25 | MPLC |  | 50 | MPLC | MPLC | 1 |
| Goodwin26 | MPLC |  | 50 | MPLC | MPLC | 1 |
| Goodwin27 | MPLC |  | 50 | MPLC | MPLC | 1 |
| Goodwin28 | MPLC |  | 50 | MPLC | IPM | 0 |
| Goodwin29 | MPLC |  | 50 | MPLC | MPLC | 1 |
| Goodwin30 | MPLC |  | 50 | MPLC | MPLC | 1 |
| Goodwin31 | MPLC |  | 50 | IPM | IPM | 0.033 |
| Goodwin32 | MPLC |  | 50 | IPM | IPM | 0.017 |
| Goodwin33 | MPLC |  | 50 | IN | IN |  |
| Goodwin34 | MPLC |  | 50 | IN | IN |  |
| Goodwin35 | MPLC |  | 50 | MPLC | MPLC | 1 |
| Goodwin36 | MPLC |  | 50 | MPLC | MPLC | 1 |
| Goodwin37 | MPLC |  | 50 | IN | IN |  |
| Goodwin38 | MPLC |  | 50 | MPLC | MPLC | 1 |
| Goodwin39 | MPLC |  | 50 | IN | IN |  |
| Goodwin40 | MPLC |  | 50 | MPLC | MPLC | 1 |
| Hu1 | MPLC |  | 1021 | MPLC | Unknown |  |
| Hu2 | MPLC |  | 1021 | MPLC | Unknown |  |
| Hu3 | MPLC |  | 1021 | MPLC | Unknown |  |
| Hu4 | MPLC |  | 1021 | MPLC | Unknown |  |
| Hu5 | MPLC |  | 1021 | MPLC | Unknown |  |
| Hu6 | MPLC |  | 1021 | MPLC | Unknown |  |
| Hu7 | MPLC |  | 1021 | MPLC | Unknown |  |
| Hu8 | MPLC |  | 1021 | MPLC | Unknown |  |
| Hu9 | MPLC |  | 1021 | MPLC | Unknown |  |
| Hu10 | MPLC |  | 1021 | MPLC | Unknown |  |
| Hu11 | MPLC |  | 1021 | MPLC | Unknown |  |
| Hu12 | MPLC |  | 1021 | IN | Unknown |  |
| Hu13 | MPLC |  | 1021 | MPLC | Unknown |  |
| Hu14 | MPLC |  | 1021 | MPLC | Unknown |  |
| Hu15 | MPLC |  | 1021 | MPLC | Unknown |  |
| Hu16 | MPLC |  | 1021 | IN | Unknown |  |
| Hu17 | MPLC |  | 1021 | MPLC | Unknown |  |
| Hu18 | MPLC |  | 1021 | MPLC | Unknown |  |
| Hu19 | MPLC |  | 1021 | MPLC | Unknown |  |
| Hu20 | MPLC |  | 1021 | MPLC | Unknown |  |
| Hu21 | MPLC |  | 1021 | MPLC | Unknown |  |
| Hu22 | MPLC |  | 1021 | MPLC | Unknown |  |
| Hu23 | MPLC |  | 1021 | MPLC | Unknown |  |
| Hu24 | MPLC |  | 1021 | MPLC | Unknown |  |
| Hu25 | MPLC |  | 1021 | MPLC | Unknown |  |
| Izumi1 | MPLC |  | 409 | IN | IN |  |
| Izumi2 | MPLC |  | 409 | MPLC | MPLC | 1 |
| Izumi3 | MPLC |  | 409 | IN | IPM | 0.033 |
| Izumi4 | MPLC |  | 409 | MPLC | MPLC | 1 |
| Izumi5 | MPLC |  | 409 | IN | IPM | 0.003 |
| Izumi6 | MPLC |  | 409 | IN | IN |  |
| Izumi7 | MPLC |  | 409 | IN | IN |  |
| Izumi8 | MPLC |  | 409 | MPLC | MPLC | 1 |
| Izumi9 | MPLC |  | 409 | MPLC | MPLC | 1 |
| Izumi10 | MPLC |  | 409 | MPLC | MPLC | 1 |
| Izumi11 | MPLC |  | 409 | MPLC | MPLC | 1 |
| Izumi12 | MPLC |  | 409 | MPLC | IPM | 0.001 |
| Izumi13 | MPLC |  | 409 | IN | IPM | 0.017 |
| Izumi14 | MPLC |  | 409 | IPM | IPM | 0 |
| Izumi15 | MPLC |  | 409 | MPLC | MPLC | 1 |
| Izumi16 | MPLC |  | 409 | IN | IPM | 0.033 |
| Izumi17 | MPLC |  | 409 | IN | IPM | 0.004 |
| Pei1 | MPLC |  | 808 | MPLC | MPLC | 1 |
| Pei2 | MPLC |  | 808 | IN | IN |  |
| Pei3 | MPLC |  | 808 | MPLC | MPLC | 1 |
| Pei4 | IPM |  | 808 | MPLC | MPLC | 1 |
| Pei5 | IPM |  | 808 | MPLC | MPLC | 1 |
| Pei6 | MPLC |  | 808 | IN | IN |  |
| Pei7 | MPLC |  | 808 | MPLC | MPLC | 1 |
| Pei8 | IPM |  | 808 | IPM | IPM | 0 |
| Pei9 | MPLC |  | 808 | IPM | IPM | 0 |
| Pei10 | MPLC |  | 808 | MPLC | MPLC | 1 |
| Pei11 | IPM |  | 808 | MPLC | MPLC | 1 |
| Pei12 | MPLC |  | 808 | MPLC | MPLC | 1 |
| Pei13 | MPLC |  | 808 | MPLC | MPLC | 1 |
| Pei14 | IPM |  | 808 | MPLC | MPLC | 1 |
| Pei15 | MPLC |  | 808 | MPLC | MPLC | 1 |
| Pei16 | IPM |  | 808 | MPLC | IPM | 0.039 |
| Pei17 | IPM |  | 808 | MPLC | IPM | 0.025 |
| Pei18 | IPM |  | 808 | MPLC | MPLC | 1 |
| Pei19 | IPM |  | 808 | MPLC | MPLC | 1 |
| Pei20 | MPLC |  | 808 | MPLC | IPM | 0 |
| Pei21 | MPLC |  | 808 | MPLC | MPLC | 1 |
| Pei22 | IPM |  | 808 | IN | IN |  |
| Pei23 | IPM |  | 808 | MPLC | IPM | 0.029 |
| Pei24 | MPLC |  | 808 | IN | IN |  |
| Pei25 | MPLC |  | 808 | IN | IN |  |
| Pei26 | MPLC |  | 808 | MPLC | MPLC | 1 |
| Pei27 | MPLC |  | 808 | MPLC | MPLC | 1 |
| Pei28 | IPM |  | 808 | IN | IN |  |
| Pei29 | MPLC |  | 808 | IPM | IPM | 0.031 |
| Pei30 | MPLC |  | 808 | IN | IN |  |
| Qu1 | IPM |  | 1 | MPLC | MPLC | 1 |
| Qu2 | IPM |  | 1 | MPLC | MPLC | 1 |
| Qu3 | IPM |  | 1 | MPLC | MPLC | 1 |
| Qu4 | MPLC |  | 1 | IN | IN |  |
| Qu5 | IPM |  | 1 | IN | IN |  |
| Qu6 | IPM |  | 1 | IN | IN |  |
| Qu7 | MPLC |  | 1 | IN | IN |  |
| Qu8 | MPLC |  | 1 | MPLC | MPLC | 1 |
| Yang1 |  |  | 410 | MPLC | IPM | 0.034 |
| Yang2 |  |  | 410 | IN | IPM | 0.034 |
| Yang3 |  |  | 410 | IPM | IPM | 0 |
| Yang4 |  |  | 410 | IPM | IPM | 0 |
| Yang5 |  |  | 410 | IN | IPM | 0.016 |
| Yang6 |  |  | 410 | IN | IPM | 0.016 |
| Yang7 |  |  | 410 | IN | IPM | 0.016 |
| Yang8 |  |  | 410 | MPLC | MPLC | 1 |
| Yang9 |  |  | 410 | IPM | IPM | 0 |
| Yang10 |  |  | 410 | IPM | IPM | 0 |
| Yang11 |  |  | 410 | IPM | IPM | 0 |
| Yang12 |  |  | 410 | IN | IPM | 0.033 |
| Yang13 |  |  | 410 | IN | IPM | 0.033 |
| Yang14 |  |  | 410 | IN | IPM | 0.033 |
| Yang15 |  |  | 410 | IN | IPM | 0.048 |
| Yang16 |  |  | 410 | IPM | IPM | 0 |
| Yang17 |  |  | 410 | IPM | IPM | 0 |
| Yang18 |  |  | 410 | IN | IPM | 0 |
| Yang19 |  |  | 410 | IN | IPM | 0.014 |
| Yang20 |  |  | 410 | IPM | IPM | 0 |
| Yang21 |  |  | 410 | IPM | IPM | 0 |
| Yang22 |  |  | 410 | IPM | IPM | 0.001 |
| Yang23 |  |  | 410 | IPM | IPM | 0 |
| Yang24 |  |  | 410 | IPM | IPM | 0 |
| Zhang1 | MPLC |  | 10 | IN | IN |  |
| Zhang2 | MPLC |  | 10 | MPLC | IPM | 0.024 |
| Zhang3 | MPLC |  | 10 | IN | IN |  |
| Zhang4 | MPLC |  | 10 | MPLC | MPLC | 1 |
| Zhang5 | MPLC |  | 10 | MPLC | MPLC | 1 |
| Zhang6 | MPLC |  | 10 | MPLC | MPLC | 1 |
| Zhang7 | MPLC |  | 10 | MPLC | MPLC | 1 |
| Zhang8 | MPLC |  | 10 | MPLC | MPLC | 1 |
| Zhang9 | MPLC |  | 10 | MPLC | MPLC | 1 |
| Zhang10 | MPLC |  | 10 | IN | IN |  |
| Zhang11 | MPLC |  | 10 | MPLC | MPLC | 1 |
| Zhang12 | MPLC |  | 10 | IN | IN |  |
| Zhang13 | MPLC |  | 10 | IN | IN |  |
| Zhang14 | MPLC |  | 10 | MPLC | MPLC | 1 |
| Zhang15 | MPLC |  | 10 | MPLC | MPLC | 1 |
| Zhang16 | MPLC |  | 10 | MPLC | IPM | 0.033 |
| Zhang17 | MPLC |  | 10 | IN | IN |  |
| Zhang18 | MPLC |  | 10 | IN | IN |  |
| Zhang19 | MPLC |  | 10 | IN | IN |  |
| Zhang20 | MPLC |  | 10 | IN | IPM | 0.017 |
| Zhang21 | MPLC |  | 10 | MPLC | MPLC | 1 |
| Zhang22 | MPLC |  | 10 | MPLC | MPLC | 1 |
| Zhang23 | MPLC |  | 10 | IN | IN |  |
| Zhang24 | MPLC |  | 10 | MPLC | MPLC | 1 |
| Zhang25 | MPLC |  | 10 | MPLC | MPLC | 1 |
| Zhang26 | MPLC |  | 10 | MPLC | MPLC | 1 |
| Zhang27 | MPLC |  | 10 | MPLC | IPM | 0.017 |
| Zhang28 | MPLC |  | 10 | MPLC | MPLC | 1 |
| Zhang29 | MPLC |  | 10 | IN | IN |  |
| Zhang30 | MPLC |  | 10 | IN | IN |  |
| Zhang31 | MPLC |  | 10 | MPLC | MPLC | 1 |
| Zhang32 | MPLC |  | 10 | IN | IN |  |
| Zhang33 | MPLC |  | 10 | IN | IN |  |
| Zhang34 | MPLC |  | 10 | IN | IN |  |
| Zhang35 | MPLC |  | 10 | IN | IN |  |
| Zhang36 | MPLC |  | 10 | IN | IN |  |
| Zhang37 | MPLC |  | 10 | MPLC | MPLC | 1 |
| Zhang38 | MPLC |  | 10 | MPLC | MPLC | 1 |
| Zhang39 | MPLC |  | 10 | IN | IN |  |
| Zhang40 | MPLC |  | 10 | MPLC | MPLC | 1 |
| Zhang41 | MPLC |  | 10 | MPLC | MPLC | 1 |
| Zhang42 | MPLC |  | 10 | MPLC | IPM | 0.034 |
| Wanga1 | MPLC |  | 605 | MPLC | MPLC | 1 |
| Wanga2 | MPLC |  | 605 | MPLC | MPLC | 1 |
| Wanga3 | MPLC |  | 605 | MPLC | MPLC | 1 |
| Wanga4 | MPLC |  | 605 | MPLC | MPLC | 1 |
| Wanga5 | MPLC |  | 605 | MPLC | MPLC | 1 |
| Wanga6 | MPLC |  | 605 | IN | IN |  |
| Wanga7 | MPLC |  | 605 | MPLC | MPLC | 1 |
| Wanga8 | MPLC |  | 605 | MPLC | MPLC | 1 |
| Wanga9 | MPLC |  | 605 | MPLC | MPLC | 1 |
| Wanga10 | MPLC |  | 605 | IN | IPM | 0.035 |
| Wanga11 | MPLC |  | 605 | IN | IPM | 0.017 |
| Wanga12 | MPLC |  | 605 | IN | IPM | 0.068 |
| Wanga13 | MPLC |  | 605 | MPLC | MPLC | 1 |
| Wanga14 | MPLC |  | 605 | MPLC | MPLC | 1 |
| Wanga15 | MPLC |  | 605 | MPLC | MPLC | 1 |
| Wanga16 | MPLC |  | 605 | MPLC | MPLC | 1 |
| Wanga17 | MPLC |  | 605 | MPLC | MPLC | 1 |
| Wanga18 | MPLC |  | 605 | MPLC | MPLC | 1 |
| Wanga19 | MPLC |  | 605 | MPLC | MPLC | 1 |
| Wanga20 | MPLC |  | 605 | MPLC | MPLC | 1 |
| Wanga21 | MPLC |  | 605 | IN | IPM | 0.04 |
| Wanga22 | MPLC |  | 605 | MPLC | MPLC | 1 |
| Wanga23 | MPLC |  | 605 | MPLC | MPLC | 1 |
| Wanga24 | MPLC |  | 605 | IPM | IPM | 0 |
| Wanga25 | MPLC |  | 605 | IN | IPM | 0.033 |
| Wanga26 | MPLC |  | 605 | IN | IPM | 0.035 |
| Wanga27 | MPLC |  | 605 | IN | IPM | 0.033 |
| Wanga28 | MPLC |  | 605 | MPLC | MPLC | 1 |
| Wanga29 | MPLC |  | 605 | IN | IPM | 0.004 |
| Wanga30 | MPLC |  | 605 | IN | IPM | 0.006 |
| Wanga31 | IPM |  | 605 | IPM | IPM | 0 |
| Wanga32 | IPM |  | 605 | IPM | IPM | 0 |
| Wanga33 | IPM |  | 605 | IPM | IPM | 0 |
| Wanga34 | IPM |  | 605 | IPM | IPM | 0 |
| Wanga35 | IPM |  | 605 | IPM | IPM | 0 |
| Wangb1 | MPLC |  | 605 | MPLC | MPLC | 1 |
| Wangb2 | MPLC |  | 605 | MPLC | MPLC | 1 |
| Wangb3 | MPLC |  | 605 | MPLC | MPLC | 1 |
| Wangb4 | MPLC |  | 605 | MPLC | MPLC | 1 |
| Wangb5 | MPLC |  | 605 | MPLC | MPLC | 1 |
| Wangb6 | MPLC |  | 605 | MPLC | MPLC | 1 |
| Wangb7 | MPLC |  | 605 | MPLC | MPLC | 1 |
| Wangb8 | MPLC |  | 605 | IN | IN |  |
| Wangb9 | MPLC |  | 605 | MPLC | MPLC | 1 |
| Wangb10 | MPLC |  | 605 | MPLC | MPLC | 1 |
| Wangb11 | MPLC |  | 605 | IN | IN |  |
| Wangb12 | MPLC |  | 605 | IN | IPM | 0.027 |
| Wangb13 | MPLC |  | 605 | MPLC | MPLC | 1 |
| Wangb14 | MPLC |  | 605 | IN | IPM | 0.003 |
| Wangb15 | IPM |  | 605 | IPM | IPM | 0 |
| Wangb16 | IPM |  | 605 | IPM | IPM | 0 |
| Chen1 | MPLC | MPLC | 168 | IN | MPLC | 1 |
| Chen2 | MPLC | MPLC | 168 | IN | IPM | 0.033 |
| Chen3 | MPLC | MPLC | 168 | IN | IPM | 0 |
| Chen4 | MPLC | MPLC | 168 | IN | MPLC | 1 |
| Chen5 | MPLC | MPLC | 168 | IN | IPM | 0.017 |
| Chen6 | MPLC | MPLC | 168 | IN | IPM | 0.017 |
| Chen7 | MPLC | MPLC | 168 | IN | MPLC | 1 |
| Chen8 | IPM | IPM | 168 | IPM | IPM | 0 |
| Chen9 | IPM | IPM | 168 | IPM | IPM | 0 |
| Chen10 | IPM | IPM | 168 | IPM | IPM | 0 |
| Chen11 | IPM | IPM | 168 | IPM | IPM | 0 |
| Chen12 | MPLC | MPLC | 168 | MPLC | MPLC | 1 |
| Chen13 | MPLC | MPLC | 168 | IN | IPM | 0.017 |
| Chen14 | MPLC | MPLC | 168 | MPLC | MPLC | 1 |
| Chen15 | MPLC | IPM | 168 | IN | IN |  |
| Chen16 | MPLC | MPLC | 168 | MPLC | MPLC | 1 |
| Chen17 | MPLC | MPLC | 168 | MPLC | MPLC | 1 |
| Donfrancesco1 | IN | MPLC | 22 | IN | IN |  |
| Donfrancesco2 | MPLC | MPLC | 22 | IN | IN |  |
| Donfrancesco3 | MPLC | MPLC | 22 | IN | IN |  |
| Donfrancesco4 | IPM | IPM | 22 | IPM | IPM | 0.048 |
| Donfrancesco5 | MPLC | IPM | 22 | IN | IN |  |
| Donfrancesco6 | IPM | IPM | 22 | IN | IPM | 0.068 |
| Donfrancesco7 | IPM | IPM | 22 | IPM | MPLC | 1 |
| Donfrancesco8 | IPM | IPM | 22 | IN | IN |  |
| Donfrancesco9 | IPM | MPLC | 22 | MPLC | MPLC | 1 |
| Donfrancesco10 | MPLC | MPLC | 22 | MPLC | MPLC | 1 |
| Donfrancesco11 | MPLC | MPLC | 22 | MPLC | MPLC | 1 |
| Donfrancesco12 | MPLC | MPLC | 22 | IN | IN |  |
| Donfrancesco13 | MPLC | MPLC | 22 | IN | IN |  |
| Donfrancesco14 | IN | MPLC | 22 | IN | IN |  |
| Donfrancesco15 | MPLC | IPM | 22 | IN | IN |  |
| Donfrancesco16 | MPLC | IPM | 22 | MPLC | MPLC | 1 |
| Donfrancesco17 | IPM | IPM | 22 | IPM | IPM | 0 |
| Donfrancesco18 | MPLC | IPM | 22 | IN | IN |  |
| Donfrancesco19 | MPLC | IPM | 22 | IN | IN |  |
| Donfrancesco20 | MPLC | MPLC | 22 | IN | IN |  |
| Donfrancesco21 | MPLC | MPLC | 22 | IN | IN |  |
| Donfrancesco22 | MPLC | MPLC | 22 | IN | IN |  |
| Donfrancesco23 | IN | IPM | 22 | IN | IN |  |
| Donfrancesco24 | MPLC | MPLC | 22 | MPLC | MPLC | 1 |
| Duan1 | MPLC |  | 520 | MPLC | MPLC | 1 |
| Duan2 | MPLC |  | 520 | MPLC | MPLC | 1 |
| Duan3 | MPLC |  | 520 | MPLC | MPLC | 1 |
| Duan4 | MPLC |  | 520 | MPLC | MPLC | 1 |
| Duan5 | MPLC |  | 520 | IPM | IPM | 0 |
| Duan6 | MPLC |  | 520 | MPLC | MPLC | 1 |
| Duan7 | MPLC |  | 520 | MPLC | MPLC | 1 |
| Duan8 | MPLC |  | 520 | MPLC | MPLC | 1 |
| Duan9 | MPLC |  | 520 | MPLC | MPLC | 1 |
| Duan10 | MPLC |  | 520 | IPM | MPLC | 1 |
| Duan11 | MPLC |  | 520 | MPLC | MPLC | 1 |
| Duan12 | MPLC |  | 520 | MPLC | MPLC | 1 |
| Duan13 | MPLC |  | 520 | MPLC | MPLC | 1 |
| Duan14 | MPLC |  | 520 | MPLC | MPLC | 1 |
| Duan15 | MPLC |  | 520 | MPLC | IPM | 0.038 |
| Duan16 | MPLC |  | 520 | IPM | MPLC | 1 |
| Higuchi1 | MPLC | MPLC | 53 | IN | IN |  |
| Higuchi2 | MPLC | MPLC | 53 | MPLC | MPLC | 1 |
| Higuchi3 | MPLC | MPLC | 53 | MPLC | MPLC | 1 |
| Higuchi4 | IPM | IPM | 53 | MPLC | MPLC | 1 |
| Higuchi5 | MPLC | MPLC | 53 | MPLC | MPLC | 1 |
| Higuchi6 | MPLC | MPLC | 53 | MPLC | MPLC | 1 |
| Higuchi7 | MPLC | MPLC | 53 | IN | IN |  |
| Higuchi8 | MPLC | MPLC | 53 | MPLC | MPLC | 1 |
| Higuchi9 | MPLC | MPLC | 53 | MPLC | MPLC | 1 |
| Higuchi10 | IPM | MPLC | 53 | IPM | IPM | 0 |
| Higuchi11 | MPLC | MPLC | 53 | MPLC | MPLC | 1 |
| Higuchi12 | IPM | MPLC | 53 | MPLC | MPLC | 1 |
| Higuchi13 | MPLC | MPLC | 53 | MPLC | MPLC | 1 |
| Higuchi14 | MPLC | MPLC | 53 | MPLC | IPM | 0 |
| Higuchi15 | MPLC | IPM | 53 | IPM | IPM | 0 |
| Higuchi16 | MPLC | MPLC | 53 | MPLC | MPLC | 1 |
| Higuchi17 | MPLC | MPLC | 53 | MPLC | MPLC | 1 |
| Higuchi18 | IPM | IPM | 53 | MPLC | MPLC | 1 |
| Higuchi19 | MPLC | MPLC | 53 | MPLC | MPLC | 1 |
| Higuchi20 | MPLC | MPLC | 53 | MPLC | MPLC | 1 |
| Higuchi21 | MPLC | MPLC | 53 | MPLC | MPLC | 1 |
| Higuchi22 | MPLC | MPLC | 53 | MPLC | MPLC | 1 |
| Higuchi23 | MPLC | IPM | 53 | IPM | IPM | 0 |
| Higuchi24 | MPLC | MPLC | 53 | MPLC | MPLC | 1 |
| Higuchi25 | MPLC | MPLC | 53 | IPM | IPM | 0 |
| Higuchi26 | MPLC | MPLC | 53 | MPLC | MPLC | 1 |
| Higuchi27 | MPLC | MPLC | 53 | MPLC | IPM | 0.077 |
| Higuchi28 | MPLC | MPLC | 53 | MPLC | MPLC | 1 |
| Higuchi29 | MPLC | MPLC | 53 | MPLC | MPLC | 1 |
| Higuchi30 | MPLC | MPLC | 53 | IPM | IPM | 0 |
| Higuchi31 | MPLC | MPLC | 53 | MPLC | MPLC | 1 |
| Higuchi32 | MPLC | IPM | 53 | IPM | IPM | 0 |
| Higuchi33 | MPLC | IPM | 53 | IPM | IPM | 0 |
| Higuchi34 | MPLC | MPLC | 53 | MPLC | MPLC | 1 |
| Higuchi35 | MPLC | MPLC | 53 | MPLC | MPLC | 1 |
| Higuchi36 | MPLC | MPLC | 53 | MPLC | MPLC | 1 |
| Higuchi37 | MPLC | MPLC | 53 | IPM | IPM | 0 |
| LiuB1 | MPLC | MPLC | 464 | IPM | MPLC | 1 |
| LiuB2 | MPLC | MPLC | 464 | MPLC | MPLC | 1 |
| LiuB3 | MPLC | MPLC | 464 | IN | IN |  |
| LiuB4 | MPLC | MPLC | 464 | IN | IN |  |
| LiuB5 | MPLC | MPLC | 464 | IN | IN |  |
| LiuB6 | MPLC | MPLC | 464 | IN | IN |  |
| LiuB7 | MPLC | MPLC | 464 | MPLC | MPLC | 1 |
| LiuB8 | MPLC | MPLC | 464 | IN | IN |  |
| LiuB9 | MPLC | MPLC | 464 | MPLC | MPLC | 1 |
| LiuB10 | MPLC | MPLC | 464 | MPLC | MPLC | 1 |
| LiuB11 | MPLC | MPLC | 464 | MPLC | MPLC | 1 |
| LiuB12 | MPLC | MPLC | 464 | MPLC | MPLC | 1 |
| LiuB13 | MPLC | MPLC | 464 | MPLC | MPLC | 1 |
| LiuB14 |  |  | 464 | IPM | IPM | 0 |
| LiuB15 | IN | MPLC | 464 | IN | MPLC | 1 |
| LiuB16 | MPLC | MPLC | 464 | IN | IN |  |
| Pagan1 |  | MPLC | 47 | IN | IN |  |
| Pagan2 |  | MPLC | 47 | IN | MPLC | 1 |
| Pagan3 |  | MPLC | 47 | IN | IN |  |
| Pagan4 |  | MPLC | 47 | IN | MPLC | 1 |
| Pagan5 |  | MPLC | 47 | IN | IN |  |
| Pagan6 |  | MPLC | 47 | MPLC | MPLC | 1 |
| Pagan7 |  | MPLC | 47 | MPLC | MPLC | 1 |
| Pagan8 |  | MPLC | 47 | IN | IN |  |
| Pagan9 |  | MPLC | 47 | MPLC | MPLC | 1 |
| Pagan10 |  | MPLC | 47 | IN | IN |  |
| Pagan11 |  | MPLC | 47 | IN | IN |  |
| Pagan12 |  | MPLC | 47 | IN | IN |  |
| Pagan13 |  | MPLC | 47 | IN | IN |  |
| Pagan14 |  | MPLC | 47 | MPLC | MPLC | 1 |
| Pagan15 |  | MPLC | 47 | IN | IN |  |
| Pagan16 |  | MPLC | 47 | MPLC | MPLC | 1 |
| Pagan17 |  | MPLC | 47 | IN | IN |  |
| Pagan18 |  | MPLC | 47 | MPLC | IPM | 0.033 |
| Pagan19 |  | MPLC | 47 | MPLC | MPLC | 1 |
| Pagan20 |  | MPLC | 47 | MPLC | IPM | 0 |
| Pagan21 |  | IPM | 47 | IPM | IPM | 0.016 |
| Pagan22 |  | IPM | 47 | IPM | IPM | 0.016 |
| Pagan23 |  | IPM | 47 | IN | IPM | 0.033 |
| Pagan24 |  | IPM | 47 | IN | IPM | 0.033 |
| Pagan25 |  | IPM | 47 | IN | IPM | 0 |
| Pagan26 |  | IPM | 47 | IN | IPM | 0.016 |
| Pagan27 |  | IPM | 47 | IN | IN |  |
| Pagan28 |  | IPM | 47 | IN | IN |  |
| Pagan29 |  | IPM | 47 | IN | IN |  |
| Pagan30 |  | IPM | 47 | IN | IN |  |
| Pagan31 |  | IPM | 47 | MPLC | IPM | 0 |
| Pagan32 |  | MPLC | 47 | IPM | IPM | 0 |
| Pagan33 |  | MPLC | 47 | IPM | IPM | 0.017 |
| Pagan34 |  | MPLC | 47 | IPM | IPM | 0.017 |
| Pagan35 |  | MPLC | 47 | IPM | IPM | 0.004 |
| Pagan36 |  | MPLC | 47 | IPM | IPM | 0 |
| Pagan37 |  | MPLC | 47 | IN | IPM | 0.017 |
| Pagan38 |  | MPLC | 47 | IPM | MPLC | 1 |
| Pagan39 |  | MPLC | 47 | IN | IN |  |
| Pagan40 |  | MPLC | 47 | IPM | IPM | 0 |
| Pagan41 |  | MPLC | 47 | IN | IN |  |
| Pagan42 |  | IPM | 47 | IN | IN |  |
| Pagan43 |  | IPM | 47 | IN | IN |  |
| Pagan44 |  | IPM | 47 | IN | IN |  |
| Pagan45 |  | MPLC | 47 | IN | IN |  |
| Pagan46 |  | MPLC | 47 | IN | IN |  |
| Pagan47 |  | MPLC | 47 | IN | IN |  |
| QiuB1 |  | IPM | 22 | IPM | IPM | 0 |
| QiuB2 |  | IPM | 22 | IN | MPLC | 1 |
| QiuB3 |  | IPM | 22 | IN | IPM | 0 |
| QiuB4 |  | IPM | 22 | IPM | IPM | 0 |
| QiuB5 |  | IPM | 22 | IPM | IPM | 0 |
| QiuB6 |  | IPM | 22 | IPM | IPM | 0 |
| QiuB7 |  | IPM | 22 | IN | IPM | 0.01 |
| QiuB8 |  | IPM | 22 | IPM | IPM | 0 |
| QiuB9 |  | IPM | 22 | IN | IN |  |
| QiuB10 |  | IPM | 22 | IN | IN |  |
| QiuB11 |  | IPM | 22 | IPM | IPM | 0 |
| QiuB12 |  | IPM | 22 | IN | IN |  |
| QiuB13 |  | IPM | 22 | IN | IN |  |
| QiuB14 |  | IPM | 22 | IPM | IPM | 0 |
| QiuB15 |  | IPM | 22 | IN | IPM | 0.017 |
| QiuB16 |  | IPM | 22 | IPM | IPM | 0 |
| QiuB17 |  | IPM | 22 | IN | IPM | 0.01 |
| QiuB18 |  | IPM | 22 | IN | MPLC | 1 |
| QiuB19 |  | IPM | 22 | IN | IN |  |
| QiuB20 |  | IPM | 22 | IN | IPM | 0.01 |
| QiuB21 |  | IPM | 22 | IN | IN |  |
| QiuB22 |  | IPM | 22 | IN | IPM | 0.017 |
| QiuB23 |  | IPM | 22 | IN | IPM | 0 |
| QiuB24 |  | IPM | 22 | IN | IPM | 0 |
| QiuB25 |  | IPM | 22 | IN | IPM | 0 |
| QiuB26 |  | IPM | 22 | IN | IPM | 0 |
| QiuB27 |  | IPM | 22 | IN | IN |  |
| QiuB28 |  | IPM | 22 | IN | IN |  |
| QiuB29 |  | IPM | 22 | IN | IPM | 0 |
| QiuB30 |  | IPM | 22 | IPM | IPM | 0 |
| QiuB31 |  | MPLC | 22 | MPLC | MPLC | 1 |
| QiuB32 |  | MPLC | 22 | IN | IN |  |
| QiuB33 |  | MPLC | 22 | MPLC | MPLC | 1 |
| QiuB34 |  | MPLC | 22 | IN | IPM | 0.017 |
| QiuB35 |  | MPLC | 22 | IN | IPM | 0.01 |
| QiuB36 |  | MPLC | 22 | MPLC | MPLC | 1 |
| QiuB37 |  | MPLC | 22 | MPLC | MPLC | 1 |
| QiuB38 |  | MPLC | 22 | IN | IPM | 0.01 |
| QiuB39 |  | MPLC | 22 | IN | IN |  |
| QiuB40 |  | MPLC | 22 | MPLC | MPLC | 1 |
| QiuB41 |  | MPLC | 22 | MPLC | IPM | 0.001 |
| QiuB42 |  | MPLC | 22 | IN | IN |  |
| QiuB43 |  | MPLC | 22 | IN | IN |  |
| QiuB44 |  | MPLC | 22 | MPLC | IPM | 0.011 |
| Rodriguez1 |  |  | 8 | MPLC | MPLC | 1 |
| Rodriguez2 |  |  | 8 | IN | IN |  |
| Rodriguez3 |  |  | 8 | MPLC | MPLC | 1 |
| Rodriguez4 |  |  | 8 | MPLC | MPLC | 1 |
| Rodriguez5 |  |  | 8 | MPLC | MPLC | 1 |
| Rodriguez6 |  |  | 8 | MPLC | MPLC | 1 |
| Rodriguez7 |  |  | 8 | MPLC | MPLC | 1 |
| Rodriguez8 |  |  | 8 | MPLC | MPLC | 1 |
| Rodriguez9 |  |  | 8 | MPLC | MPLC | 1 |
| Rodriguez10 |  |  | 8 | MPLC | MPLC | 1 |
| Rodriguez11 |  |  | 8 | IN | IN |  |
| Rodriguez12 |  |  | 8 | MPLC | MPLC | 1 |
| Rodriguez13 |  |  | 8 | IN | IN |  |
| Rodriguez14 |  |  | 8 | IN | IN |  |
| Rodriguez15 |  |  | 8 | MPLC | MPLC | 1 |
| Rodriguez16 |  |  | 8 | MPLC | MPLC | 1 |
| Rodriguez17 |  |  | 8 | MPLC | MPLC | 1 |
| Rodriguez18 |  |  | 8 | MPLC | MPLC | 1 |
| Rodriguez19 |  |  | 8 | IN | IN |  |
| Rodriguez20 |  |  | 8 | IPM | IPM | 0 |
| Rodriguez21 |  |  | 8 | IN | IPM | 0.033 |
| Rodriguez22 |  |  | 8 | IN | IPM | 0 |
| Rodriguez23 |  |  | 8 | IN | IPM | 0.033 |
| Rodriguez24 |  |  | 8 | IN | IPM | 0 |
| Rodriguez25 |  |  | 8 | IN | IPM | 0 |
| Rodriguez26 |  |  | 8 | IN | IPM | 0.003 |
| Rodriguez27 |  |  | 8 | IN | IPM | 0.052 |
| Rodriguez28 |  |  | 8 | IPM | IPM | 0 |
| Rodriguez29 |  |  | 8 | IN | MPLC | 1 |
| Rodriguez30 |  |  | 8 | IN | IN |  |
| Zheng1 | IPM |  | 48 | MPLC | MPLC | 1 |
| Zheng2 | MPLC | MPLC | 48 | IN | IN |  |
| Zheng3 | IPM | IPM | 48 | IPM | IPM | 0.068 |
| Zheng4 | IPM | MPLC | 48 | IN | IN |  |
| Zheng5 | IPM | IPM | 48 | IPM | IPM | 0.017 |
| Zheng6 | IPM | IPM | 48 | MPLC | MPLC | 1 |
| Zheng7 | IPM | MPLC | 48 | IN | IN |  |
| Zheng8 | IPM | IPM | 48 | MPLC | MPLC | 1 |
| Zheng9 | IPM | MPLC | 48 | MPLC | MPLC | 1 |
| Zheng10 | IPM | IPM | 48 | IPM | IPM | 0.068 |
| Zheng11 | IPM | IPM | 48 | IN | IN |  |
| Zheng12 | IPM | IPM | 48 | IN | IN |  |
| Zheng13 | IPM | IPM | 48 | IN | IN |  |
| Zheng14 | IPM | IPM | 48 | IPM | IPM | 0.068 |
| Zheng15 | IPM | IPM | 48 | MPLC | MPLC | 1 |
| Zheng16 | IPM | MPLC | 48 | IN | IN |  |
| Zheng17 | IPM | IPM | 48 | IN | IN |  |
| Zheng18 | IPM | MPLC | 48 | MPLC | MPLC | 1 |
| Mansuet-Lupo1 | MPLC | MPLC | 22 | IN | IPM | 0 |
| Mansuet-Lupo2 | MPLC | MPLC | 22 | IN | IPM | 0.017 |
| Mansuet-Lupo3 | MPLC | MPLC | 22 | IN | IPM | 0.001 |
| Mansuet-Lupo4 | MPLC | MPLC | 22 | IN | IPM | 0.034 |
| Mansuet-Lupo5 | MPLC | MPLC | 22 | IN | IPM | 0 |
| Mansuet-Lupo6 | IPM | MPLC | 22 | IN | IPM | 0 |
| Mansuet-Lupo7 | IPM | MPLC | 22 | IN | IPM | 0 |
| Mansuet-Lupo8 | IPM | MPLC | 22 | IPM | IPM | 0 |
| Mansuet-Lupo9 | IPM | MPLC | 22 | IPM | IPM | 0 |
| Mansuet-Lupo10 | IPM | MPLC | 22 | IPM | IPM | 0 |
| Mansuet-Lupo11 | MPLC | IPM | 22 | MPLC | MPLC | 0 |
| Mansuet-Lupo12 | MPLC | IPM | 22 | MPLC | MPLC | 0 |
| Mansuet-Lupo13 | MPLC | IPM | 22 | MPLC | MPLC | 1 |
| Mansuet-Lupo14 | MPLC | IPM | 22 | MPLC | MPLC | 1 |
| Mansuet-Lupo15 | MPLC | IPM | 22 | MPLC | MPLC | 1 |
| Mansuet-Lupo16 | MPLC | IPM | 22 | MPLC | MPLC | 1 |
| Mansuet-Lupo17 | MPLC | IPM | 22 | MPLC | MPLC | 1 |
| Mansuet-Lupo18 | MPLC | IPM | 22 | MPLC | MPLC | 1 |
| Mansuet-Lupo20 | MPLC | IPM | 22 | MPLC | MPLC | 1 |
| Mansuet-Lupo21 | MPLC | IPM | 22 | MPLC | MPLC | 1 |
| Mansuet-Lupo22 | IPM | IPM | 22 | MPLC | MPLC | 1 |
| Mansuet-Lupo23 | IPM | IPM | 22 | MPLC | MPLC | 1 |
| Mansuet-Lupo24 | IPM | IPM | 22 | MPLC | MPLC | 1 |
| Mansuet-Lupo25 | IPM | IPM | 22 | MPLC | MPLC | 1 |
| Mansuet-Lupo26 | IPM | IPM | 22 | MPLC | MPLC | 1 |
| Mansuet-Lupo27 | IPM | IPM | 22 | MPLC | MPLC | 1 |
| Mansuet-Lupo28 | IPM | IPM | 22 | MPLC | MPLC | 1 |
| Mansuet-Lupo29 | IPM | IPM | 22 | MPLC | MPLC | 1 |
| Mansuet-Lupo30 | IPM | IPM | 22 | MPLC | MPLC | 1 |
| Vincenten1 | MPLC | IPM | 2 | MPLC | IN |  |
| Vincenten2 | IPM | IPM | 2 | IN | IPM | 0.016 |
| Vincenten3 | IPM | IPM | 2 | IN | IPM | 0 |
| Vincenten4 | MPLC | IPM | 2 | IPM | IPM | 0 |
| Vincenten5 | IPM | IPM | 2 | IN | IPM | 0 |
| Vincenten6 | MPLC | IPM | 2 | IPM | IPM | 0 |
| Roepmann1 |  | MPLC | 50 | MPLC | MPLC | 1 |
| Roepmann2 |  | MPLC | 50 | MPLC | MPLC | 1 |
| Roepmann3 |  | MPLC | 50 | IN | IN |  |
| Roepmann4 |  | MPLC | 50 | MPLC | MPLC | 1 |
| Roepmann5 |  | MPLC | 50 | IN | IN |  |
| Roepmann6 |  | IPM | 50 | MPLC | MPLC | 1 |
| Roepmann7 |  | IPM | 50 | IPM | IPM | 0 |
| Roepmann8 |  | IPM | 50 | IN | IPM | 0 |
| Roepmann9 |  | IPM | 50 | IPM | IPM | 0 |
| Roepmann10 |  | IPM | 50 | IPM | IPM | 0 |
| Roepmann11 |  | IPM | 50 | MPLC | MPLC | 1 |
| Roepmann12 |  | MPLC | 50 | MPLC | MPLC | 1 |
| Roepmann13 |  | IPM | 50 | MPLC | MPLC | 1 |
| Roepmann14 |  | IPM | 50 | IPM | IPM | 0 |
| Roepmann15 |  | IPM | 50 | MPLC | MPLC | 1 |
| Roepmann16 |  | IPM | 50 | IN | IPM | 0 |
| Roepmann17 |  | IPM | 50 | MPLC | MPLC | 1 |
| Roepmann18 |  | IPM | 50 | IN | IN |  |
| Roepmann19 |  | IPM | 50 | MPLC | MPLC | 1 |
| Roepmann20 |  | MPLC | 50 | MPLC | MPLC | 1 |
| Roepmann21 |  | MPLC | 50 | IPM | MPLC | 1 |
| Roepmann22 |  | IPM | 50 | IN | IPM | 0 |
| Roepmann23 |  | IPM | 50 | MPLC | IPM | 0.048 |
| Roepmann24 |  | IPM | 50 | MPLC | MPLC | 1 |
| Roepmann25 |  | MPLC | 50 | MPLC | MPLC | 1 |
| Roepmann26 |  | MPLC | 50 | IN | IPM | 0.033 |
| Roepmann27 |  | IPM | 50 | IPM | IPM | 0 |
| Roepmann28 |  | MPLC | 50 | IN | IN |  |
| Roepmann29 |  | MPLC | 50 | IN | IN |  |
| Roepmann30 |  | IPM | 50 | MPLC | MPLC | 1 |
| Roepmann31 |  | MPLC | 50 | IN | IN |  |
| Roepmann32 |  | MPLC | 50 | MPLC | MPLC | 1 |
| Roepmann33 |  | MPLC | 50 | MPLC | MPLC | 1 |
| Roepmann34 |  | MPLC | 50 | MPLC | MPLC | 1 |
| Roepmann35 |  | MPLC | 50 | MPLC | MPLC | 1 |
| Roepmann36 |  | MPLC | 50 | MPLC | IPM | 0.073 |
| Roepmann37 |  | MPLC | 50 | MPLC | MPLC | 1 |
| Roepmann38 |  | MPLC | 50 | MPLC | MPLC | 1 |
| Roepmann39 |  | MPLC | 50 | MPLC | IPM | 0.076 |
| Roepmann40 |  | MPLC | 50 | MPLC | MPLC | 1 |
| Roepmann41 |  | IPM | 50 | IN | IN |  |
| Roepmann42 |  | MPLC | 50 | IN | IN |  |
| Roepmann43 |  | MPLC | 50 | MPLC | MPLC | 1 |
| Roepmann44 |  | MPLC | 50 | IN | IN |  |
| Roepmann45 |  | IPM | 50 | IPM | MPLC | 1 |
| Roepmann46 |  | IPM | 50 | IPM | MPLC | 1 |
| Roepmann47 |  | MPLC | 50 | MPLC | MPLC | 1 |
| Roepmann48 |  | MPLC | 50 | IN | IN |  |
| Roepmann49 |  | MPLC | 50 | MPLC | MPLC | 1 |
| Roepmann50 |  | MPLC | 50 | MPLC | MPLC | 1 |
| Girard1 | MPLC | MPLC | 2 | MPLC | MPLC | 1 |
| Girard2 | MPLC | MPLC | 2 | MPLC | MPLC | 1 |
| Girard3 | IPM | MPLC | 2 | MPLC | MPLC | 1 |
| Girard4 | MPLC | MPLC | 2 | MPLC | MPLC | 1 |
| Girard5 | IPM | MPLC | 2 | IN | IN |  |
| Girard6 | IPM | MPLC | 2 | MPLC | MPLC | 1 |
| Girard7 | MPLC | MPLC | 2 | MPLC | MPLC | 1 |
| Arai1 | IPM | IPM | 1 | IN | IPM | 0 |
| Arai2 | IPM | IPM | 1 | IN | IPM | 0 |
| Arai3 | IPM | IPM | 1 | IN | IN |  |
| Arai4 | IPM | IPM | 1 | IN | IPM | 0.017 |
| Arai5 | MPLC | IPM | 1 | IN | IPM | 0.017 |
| Arai6 | MPLC | IPM | 1 | IN | IN |  |
| Arai7 | MPLC | MPLC | 1 | IN | IN |  |
| Arai8 | MPLC | MPLC | 1 | IN | IN |  |
| Arai9 | MPLC | MPLC | 1 | IN | IN |  |
| Arai10 | MPLC | MPLC | 1 | IN | IN |  |
| Arai11 | MPLC | MPLC | 1 | IN | IN |  |
| Arai12 | MPLC | MPLC | 1 | MPLC | MPLC | 1 |
| Takamochi1 | MPLC |  | 2 | IN | IPM | 0.037 |
| Takamochi2 | MPLC |  | 2 | IN | IN |  |
| Takamochi3 | MPLC |  | 2 | IN | IN |  |
| Takamochi4 | MPLC |  | 2 | IN | IN |  |
| Takamochi5 | MPLC |  | 2 | IN | IN |  |
| Takamochi6 | MPLC |  | 2 | IN | IN |  |
| Takamochi7 | MPLC |  | 2 | IN | IPM | 0.017 |
| Takamochi8 | MPLC |  | 2 | IPM | MPLC | 1 |
| Takamochi9 | MPLC |  | 2 | MPLC | MPLC | 1 |
| Takamochi10 | MPLC |  | 2 | IPM | IPM | 0 |
| Takamochi11 | MPLC |  | 2 | IN | IN |  |
| Takamochi12 | MPLC |  | 2 | IN | IN |  |
| Takamochi13 | MPLC |  | 2 | IN | IN |  |
| Takamochi14 | MPLC |  | 2 | IN | IN |  |
| Takamochi15 | MPLC |  | 2 | MPLC | MPLC | 1 |
| Takamochi16 | MPLC |  | 2 | MPLC | MPLC | 1 |
| Takamochi17 | MPLC |  | 2 | MPLC | MPLC | 1 |
| Takamochi18 | MPLC |  | 2 | MPLC | MPLC | 1 |
| Takamochi19 | MPLC |  | 2 | MPLC | MPLC | 1 |
| Takamochi20 | MPLC |  | 2 | IN | IN |  |
| Takamochi21 | IPM |  | 2 | IN | IN |  |
| Takamochi22 | IPM |  | 2 | IN | IN |  |
| Takamochi23 | IPM |  | 2 | IN | IN |  |
| Takamochi24 | IPM |  | 2 | IN | IN |  |
| Takamochi25 | IPM |  | 2 | IN | IN |  |
| Takamochi26 | MPLC |  | 2 | IN | IN |  |
| Takamochi27 | MPLC |  | 2 | IPM | IPM | 0.035 |
| Takamochi28 | MPLC |  | 2 | IN | IN |  |
| Takamochi29 | MPLC |  | 2 | IN | IN |  |
| Takamochi30 | MPLC |  | 2 | IN | IN |  |
| LiuA1 | MPLC |  | 10000 | MPLC | MPLC | 1 |
| LiuA2 | MPLC |  | 10000 | MPLC | MPLC | 1 |
| LiuA3 | MPLC |  | 10000 | MPLC | MPLC | 1 |
| LiuA4 | MPLC |  | 10000 | MPLC | MPLC | 1 |
| LiuA5 | MPLC |  | 10000 | MPLC | IPM | 0.072 |
| LiuA6 | MPLC |  | 10000 | MPLC | IPM | 0.054 |
| Liu1 |  | MPLC | 1 | IN | IN |  |
| Liu2 |  | MPLC | 1 | MPLC | MPLC | 1 |
| Liu3 |  | MPLC | 1 | MPLC | MPLC | 1 |
| Liu4 |  | MPLC | 1 | MPLC | MPLC | 1 |
| Liu5 |  | MPLC | 1 | MPLC | MPLC | 1 |
| Liu6 |  | MPLC | 1 | MPLC | MPLC | 1 |
| Liu7 |  | MPLC | 1 | MPLC | MPLC | 1 |
| Liu8 |  | MPLC | 1 | MPLC | MPLC | 1 |
| Liu9 |  | MPLC | 1 | MPLC | MPLC | 1 |
| Liu10 |  | MPLC | 1 | IN | IPM | 0 |
| Liu11 |  | MPLC | 1 | IN | IPM | 0.017 |
| Liu12 |  | MPLC | 1 | MPLC | MPLC | 1 |
| Liu13 |  | MPLC | 1 | MPLC | MPLC | 1 |
| Liu14 |  | MPLC | 1 | MPLC | MPLC | 1 |
| Liu15 |  | MPLC | 1 | MPLC | MPLC | 1 |
| Liu16 |  | MPLC | 1 | MPLC | MPLC | 1 |
| Liu17 |  | MPLC | 1 | MPLC | MPLC | 1 |
| Liu18 |  | MPLC | 1 | MPLC | MPLC | 1 |
| Liu19 |  | MPLC | 1 | MPLC | MPLC | 1 |
| Liu20 |  | MPLC | 1 | MPLC | MPLC | 1 |
| Liu21 |  | MPLC | 1 | MPLC | MPLC | 1 |
| Liu22 |  | MPLC | 1 | MPLC | MPLC | 1 |
| Liu23 |  | MPLC | 1 | MPLC | MPLC | 1 |
| Liu24 |  | MPLC | 1 | MPLC | MPLC | 1 |
| Liu25 |  | MPLC | 1 | MPLC | MPLC | 1 |
| Liu26 |  | MPLC | 1 | MPLC | MPLC | 1 |
| Liu27 |  | MPLC | 1 | MPLC | MPLC | 1 |
| Liu28 |  | MPLC | 1 | IN | IPM | 0 |
| Liu29 |  | MPLC | 1 | MPLC | MPLC | 1 |
| Liu30 |  | MPLC | 1 | MPLC | MPLC | 1 |
| Liu31 |  | MPLC | 1 | MPLC | MPLC | 1 |
| Liu32 |  | MPLC | 1 | MPLC | MPLC | 1 |
| Liu33 |  | MPLC | 1 | MPLC | MPLC | 1 |
| Liu34 |  | MPLC | 1 | MPLC | MPLC | 1 |
| Liu35 |  | MPLC | 1 | IN | IN |  |
| Liu36 |  | MPLC | 1 | MPLC | MPLC | 1 |
| Liu37 |  | MPLC | 1 | MPLC | MPLC | 1 |
| Liu38 |  | MPLC | 1 | IN | IPM | 0.017 |
| Xiao1 |  |  | 50 | MPLC | Unknown |  |
| Xiao2 |  |  | 50 | MPLC | Unknown |  |
| Xiao3 |  |  | 50 | MPLC | Unknown |  |
| Xiao4 |  |  | 50 | MPLC | Unknown |  |
| Xiao5 |  |  | 50 | IPM | Unknown |  |
| Xiao6 |  |  | 50 | IPM | Unknown |  |
| Saab1 |  | MPLC | 50 | MPLC | MPLC | 1 |
| Saab2 |  | MPLC | 50 | MPLC | IN |  |
| Saab3 |  | MPLC | 50 | MPLC | MPLC | 1 |
| Saab4 |  | MPLC | 50 | MPLC | IN |  |
| Saab5 |  | MPLC | 50 | MPLC | MPLC | 1 |
| Saab6 |  | MPLC | 50 | MPLC | MPLC | 1 |
| Saab7 |  | MPLC | 50 | MPLC | MPLC | 1 |
| Saab8 |  | MPLC | 50 | MPLC | MPLC | 1 |
| Saab9 |  | MPLC | 50 | MPLC | MPLC | 1 |
| Saab10 |  | IN | 50 | MPLC | MPLC | 1 |
| Saab11 |  | MPLC | 50 | MPLC | MPLC | 1 |
| Saab12 |  |  | 50 |  | IPM | 0 |
| Saab13 |  |  | 50 |  | IPM | 0.017 |
| Saab14 |  |  | 50 |  | MPLC | 1 |
| Saab15 |  |  | 50 |  | MPLC | 1 |
| Saab16 |  |  | 50 |  | MPLC | 1 |
| Saab17 |  | MPLC | 50 | IN | MPLC | 1 |
| Saab18 |  | IPM | 50 | MPLC | MPLC | 1 |
| Patel1 | IN | MPLC | 50 | IPM | IPM | 0 |
| Patel2 | IN | MPLC | 50 | IN | IPM | 0.003 |
| Patel3 | IN | IPM | 50 | IPM | IPM | 0.003 |
| Patel4 | MPLC | MPLC | 50 | MPLC | IPM | 0.001 |
| Patel5 | MPLC | MPLC | 50 | MPLC | MPLC | 1 |
| Patel6 | MPLC | MPLC | 50 | MPLC | MPLC | 1 |
| Patel7 | MPLC | MPLC | 50 | MPLC | MPLC | 1 |
| Patel8 | MPLC | MPLC | 50 | IN | IN |  |
| Patel9 | MPLC | MPLC | 50 | MPLC | MPLC | 1 |
| Patel10 | MPLC | MPLC | 50 | IN | IN |  |
| Patel11 | MPLC | MPLC | 50 | IN | IN |  |
| Goto1 |  |  | 53 | MPLC | MPLC | 1 |
| Goto2 |  |  | 53 | MPLC | MPLC | 1 |
| Goto3 |  |  | 53 | IPM | IPM | 0 |
| Goto4 |  |  | 53 | MPLC | MPLC | 1 |
| Goto5 |  |  | 53 | MPLC | MPLC | 1 |
| Goto6 |  |  | 53 | MPLC | MPLC | 1 |
| Goto7 |  |  | 53 | MPLC | MPLC | 1 |
| Goto8 |  |  | 53 | MPLC | MPLC | 1 |
| Goto9 |  |  | 53 | MPLC | MPLC | 1 |
| Goto10 |  |  | 53 | MPLC | MPLC | 1 |
| Goto11 |  |  | 53 | MPLC | MPLC | 1 |
| Goto12 |  |  | 53 | MPLC | MPLC | 1 |
| Asmar1 |  | IPM | 2 | IN | MPLC | 1 |
| Asmar2 |  | MPLC | 2 | IN | IN |  |
| Asmar3 |  | MPLC | 2 | IN | IN |  |
| Asmar4 |  | IPM | 2 | IN | IN |  |
| Asmar5 |  | MPLC | 2 | IN | IN |  |
| Asmar6 |  | IPM | 2 | IN | IPM | 0 |
| Asmar7 |  | MPLC | 2 | IN | IN |  |
| Asmar8 |  | MPLC | 2 | IPM | IPM | 0 |
| Asmar9 |  | IPM | 2 | IN | IN |  |
| Asmar10 |  | MPLC | 2 | IN | IN |  |
| Asmar11 |  | MPLC | 2 | MPLC | MPLC | 1 |
| Asmar12 |  | MPLC | 2 | IN | IN |  |
| Asmar13 |  | MPLC | 2 | MPLC | MPLC | 1 |
| Asmar14 |  | IPM | 2 | MPLC | MPLC | 1 |
| Asmar15 |  | IPM | 2 | IN | IN |  |
| Asmar16 |  | MPLC | 2 | IN | MPLC | 1 |
| Asmar17 |  | MPLC | 2 | MPLC | MPLC | 1 |
| Asmar18 |  | MPLC | 2 | IN | IN |  |
| Asmar19 |  | MPLC | 2 | IN | IN |  |
| Asmar20 |  | IPM | 2 | IN | IPM | 0.017 |
| Asmar21 |  | MPLC | 2 | IN | IN |  |
| Asmar22 |  | MPLC | 2 | IN | IPM | 0.017 |
| Asmar23 |  | MPLC | 2 | IN | IN |  |
| Asmar24 |  | IPM | 2 | MPLC | MPLC | 1 |
| Asmar25 |  | MPLC | 2 | IN | IN |  |
| Asmar26 |  | IPM | 2 | IN | IN |  |
| Asmar27 |  | IPM | 2 | IN | IPM | 0 |
| Asmar28 |  | IPM | 2 | IN | IN |  |
| Asmar29 |  | MPLC | 2 | MPLC | MPLC | 1 |
| Asmar30 |  | MPLC | 2 | IN | IN |  |
| Asmar31 |  | MPLC | 2 | IN | IPM | 0 |
| Asmar32 |  | MPLC | 2 | IN | IPM | 0 |
| Asmar33 |  | IPM | 2 | IN | IN |  |
| Asmar34 |  | MPLC | 2 | IN | IPM | 0 |
| Asmar35 |  | MPLC | 2 | IN | IN |  |
| Asmar36 |  | MPLC | 2 | IN | IN |  |
| Asmar37 |  | MPLC | 2 | IN | IPM | 0 |
| Asmar38 |  | MPLC | 2 | IN | IPM | 0 |
| Asmar39 |  | IPM | 2 | MPLC | MPLC | 1 |
| Asmar40 |  | MPLC | 2 | IN | IN |  |
| Asmar41 |  | MPLC | 2 | MPLC | MPLC | 1 |
| Asmar42 |  | IPM | 2 | IN | IN |  |
| Asmar43 |  | MPLC | 2 | IN | IN |  |
| Asmar44 |  | IPM | 2 | IN | IN |  |
| Asmar45 |  | IPM | 2 | IN | IPM | 0 |
| Asmar46 |  | MPLC | 2 | IN | IN |  |
| Asmar47 |  | IPM | 2 | MPLC | MPLC | 1 |
| Asmar48 |  | MPLC | 2 | IN | IN |  |
| Asmar49 |  | MPLC | 2 | IN | IN |  |
| Asmar50 |  | IPM | 2 | IN | IN |  |
| Asmar51 |  | MPLC | 2 | IN | IN |  |
| Asmar52 |  | IPM | 2 | IN | IN |  |
| Asmar53 |  | MPLC | 2 | IN | IPM | 0.016 |
| Asmar54 |  | IPM | 2 | IN | IN |  |
| Asmar55 |  | IPM | 2 | IN | IPM | 0.016 |
| Asmar56 |  | IPM | 2 | IN | IN |  |
| Asmar57 |  | MPLC | 2 | IN | IN |  |
| Asmar58 |  | MPLC | 2 | MPLC | MPLC | 1 |
| Asmar59 |  | MPLC | 2 | IN | IN |  |
| Asmar60 |  | MPLC | 2 | MPLC | MPLC | 1 |
| Asmar61 |  | MPLC | 2 | IN | IN |  |
| Asmar62 |  | MPLC | 2 | IN | IN |  |
| Asmar63 |  | MPLC | 2 | IN | IN |  |
| Asmar64 |  | IPM | 2 | MPLC | MPLC | 1 |
| Asmar65 |  | IPM | 2 | IN | IPM | 0 |
| Asmar66 |  | MPLC | 2 | IN | IN |  |
| Asmar67 |  | IPM | 2 | IN | IPM | 0.004 |
| Asmar68 |  | MPLC | 2 | IN | IN |  |
| Asmar69 |  | IPM | 2 | IN | IPM | 0.033 |

# Reference

1. Martini N, Melamed MR. Multiple primary lung cancers. J Thorac Cardiovasc Surg. 1975;70(4):606-12.

2. Kozower BD, Larner JM, Detterbeck FC, Jones DR. Special treatment issues in non-small cell lung cancer: Diagnosis and management of lung cancer, 3rd ed: American College of Chest Physicians evidence-based clinical practice guidelines. Chest. 2013;143(5 Suppl):e369S-e99S.

3. Detterbeck FC, Bolejack V, Arenberg DA, Crowley J, Donington JS, Franklin WA, et al. The IASLC Lung Cancer Staging Project: Background Data and Proposals for the Classification of Lung Cancer with Separate Tumor Nodules in the Forthcoming Eighth Edition of the TNM Classification for Lung Cancer. Journal of thoracic oncology : official publication of the International Association for the Study of Lung Cancer. 2016;11(5):681-92.

4. Girard N, Deshpande C, Lau C, Finley D, Rusch V, Pao W, et al. Comprehensive histologic assessment helps to differentiate multiple lung primary nonsmall cell carcinomas from metastases. Am J Surg Pathol. 2009;33(12):1752-64.

5. Detterbeck FC, Franklin WA, Nicholson AG, Girard N, Arenberg DA, Travis WD, et al. The IASLC Lung Cancer Staging Project: Background Data and Proposed Criteria to Distinguish Separate Primary Lung Cancers from Metastatic Foci in Patients with Two Lung Tumors in the Forthcoming Eighth Edition of the TNM Classification for Lung Cancer. Journal of thoracic oncology : official publication of the International Association for the Study of Lung Cancer. 2016;11(5):651-65.

6. Detterbeck FC, Bolejack V, Arenberg DA, Crowley J, Donington JS, Franklin WA, et al. The IASLC Lung Cancer Staging Project: Background Data and Proposals for the Classification of Lung Cancer with Separate Tumor Nodules in the Forthcoming Eighth Edition of the TNM Classification for Lung Cancer. J Thorac Oncol. 2016;11(5):681-92.

7. Travis WD, Brambilla E, Nicholson AG, Yatabe Y, Austin JHM, Beasley MB, et al. The 2015 World Health Organization Classification of Lung Tumors: Impact of Genetic, Clinical and Radiologic Advances Since the 2004 Classification. Journal of thoracic oncology : official publication of the International Association for the Study of Lung Cancer. 2015;10(9):1243-60.

8. Mauguen A, Seshan VE, Begg CB, Ostrovnaya I. Testing clonal relatedness of two tumors from the same patient based on their mutational profiles: update of the Clonality R package. Bioinformatics. 2019;35(22):4776-8.

9. Zhou W, Chen T, Chong Z, Rohrdanz MA, Melott JM, Wakefield C, et al. TransVar: a multilevel variant annotator for precision genomics. Nat Methods. 2015;12(11):1002-3.

10. Knobloch K, Yoon U, Vogt PM. Preferred reporting items for systematic reviews and meta-analyses (PRISMA) statement and publication bias. J Craniomaxillofac Surg. 2011;39(2):91-2.

11. Dendukuri N, Schiller I, Joseph L, Pai M. Bayesian meta-analysis of the accuracy of a test for tuberculous pleuritis in the absence of a gold standard reference. Biometrics. 2012;68(4):1285-93.

12. Dendukuri N, Hadgu A, Wang L. Modeling conditional dependence between diagnostic tests: a multiple latent variable model. Stat Med. 2009;28(3):441-61.

13. Chen K, Yang A, Carbone DP, Kanu N, Liu K, Wang R, et al. Spatiotemporal genomic analysis reveals distinct molecular features in recurrent stage I non-small cell lung cancers. Cell Rep. 2022;40(2):111047.

14. Bernatsky S, Joseph L, Bélisle P, Boivin JF, Rajan R, Moore A, et al. Bayesian modelling of imperfect ascertainment methods in cancer studies. Stat Med. 2005;24(15):2365-79.

15. Tierney JF, Stewart LA, Ghersi D, Burdett S, Sydes MR. Practical methods for incorporating summary time-to-event data into meta-analysis. Trials. 2007;8:16.
